# Decisions with Uncertain Consequences—A Total Ordering on Loss-Distributions

**DOI:** 10.1371/journal.pone.0168583

**Published:** 2016-12-28

**Authors:** Stefan Rass, Sandra König, Stefan Schauer

**Affiliations:** 1 Universität Klagenfurt, Institute of Applied Informatics, Klagenfurt, Austria; 2 Austrian Institute of Technology, Safety & Security Department, Klagenfurt, Austria; National Institute of Environmental Health Sciences, UNITED STATES

## Abstract

Decisions are often based on imprecise, uncertain or vague information. Likewise, the consequences of an action are often equally unpredictable, thus putting the decision maker into a twofold jeopardy. Assuming that the effects of an action can be modeled by a random variable, then the decision problem boils down to comparing different effects (random variables) by comparing their distribution functions. Although the full space of probability distributions cannot be ordered, a properly restricted subset of distributions can be totally ordered in a practically meaningful way. We call these *loss-distributions*, since they provide a substitute for the concept of loss-functions in decision theory. This article introduces the theory behind the necessary restrictions and the hereby constructible total ordering on random loss variables, which enables decisions under uncertainty of consequences. Using data obtained from simulations, we demonstrate the practical applicability of our approach.

## 1 Introduction

In many practical situations, decision making is a matter of urgent and important choices being based on vague, fuzzy and mostly empirical information. While reasoning under uncertainty in the sense of making decision with known consequences under uncertain preconditions is a well-researched field (cf. [1–7] to name only a few), taking decisions with *uncertain consequences* has received substantially less attention. This work presents a decision framework to take the best choice from a set of options, whose consequences or benefit for the decision maker are available only in terms of a random variable. More formally, we describe a method to choose the best among two possible random variables *R*_1_, *R*_2_ by constructing a novel stochastic order on a suitably restricted subset of probability distributions. Our ordering will be total, so that the preference between two actions with random consequences *R*_1_, *R*_2_ is always well-defined and a decision can be made. As it has been shown in [8, 9], there exist several applications where such a framework of decision making on abstract spaces of random variables is needed.

To illustrate our method, we will use a couple of example data sets, the majority of which comes from the risk management context. In risk management, decisions typically have uncertain consequences that cannot be measured by a conventional von Neumann-Morgenstern utility function. For example, a security incident in a large company can either be made public, or kept secret. The uncertainty in this case is either coming from the public community’s response, if the incident is made public (as analyzed by, e.g., [10]), or the residual risk of information leakage (e.g., by whistleblowing). The question here is: Which is the better choice, given that the outcomes can be described by random variables? For such a scenario, suitable methods to determine the consequence distributions using simulations are available [10], but those methods don’t support the decision making process directly.

Typically, risk management is concerned with extreme events, since small distortions may be covered by the natural resilience of the analyzed system (e.g., by an organization’s infrastructure or the enterprise itself, etc.). For this reason, decisions normally depend on the distribution’s tails. Indeed, heavy- and fat-tailed distributions are common choices to model rare but severe incidents in general risk management [11, 12]. We build our construction with this requirement of risk management in mind, but originate from the recognized importance that the moments of a distribution play for decision making (cf. [13]). In section 3, we illustrate a simple use of the first moment in this regard that is common in IT risk management, to motivate the need to include more information in a decision. Interestingly, the ordering that we define here is based on the full moment sequence (cf. Definition 2), but implies similar conditions as other stochastic orders, only with an explicit focus on the probability mass located in the distribution’s tails (cf. Theorem 2). Further, we pick some example data sets from risk management applications in Section 5.2, and demonstrate how a decision can be made based on empirical data.

The main contribution of this work is twofold: while any stochastic order could be used for decision making on actions with random variables describing their outcome, not all of them are equally suitable in a risk management context. The ordering we present in this article is specifically designed to fit into this area. Second, the technique of constructing the ordering is new and perhaps of independent scientific interest having applications beyond our context. In the theoretical parts, this work is a condensed version of [14, 15] (provided as supporting information S1 File), whereas it extends this preliminary research by practical examples and concrete algorithms to efficiently choose best actions despite random consequences and with a sound practical meaning.

## 2 Preliminaries and Notation

Sets, random variables and probability distribution functions are denoted as upper-case letters like *X* or *F*. Matrices and vectors are denoted as bold-face upper- and lower-case letters, respectively. The symbols |*X*|, |*x*| denote the cardinality of the finite set *X* or the absolute value of the scalar x∈IR. The *k*-fold cartesian product (with *k* = ∞ permitted) is *X*^*k*^, and *X*^∞^ is the set of all infinite sequences ${\left(a_{n}\right)}_{n\in \text{I}\;\text{N}}=\left(a_{1},a_{2},a_{3},\ldots \right)$ over *X*. Calligraphic letters like F denote families (sets) of sets or functions. The symbol ${}^{*}\text{I}\;\text{R}$ denotes the space of hyperreal numbers, being a certain quotient space constructed as ${}^{*}\text{I}\;\text{R}={\text{I}\;\text{R}}^{\infty }U$, where U is a free ultrafilter. We refer to [16, 17] for details, as ${}^{*}\text{I}\;\text{R}$ is only a technical vehicle whose detailed structure is less important than the fact that it is a totally ordered field. Our construction of a total ordering on loss distributions will crucially hinge on an embedding of random variables into ${}^{*}\text{I}\;\text{R}$, where a natural ordering and full fledged arithmetic are already available without any further efforts.

The symbol *X* ∼ *F*_*X*_ means the random variable (RV) *X* having distribution *F*_*X*_, where the subscript is omitted if things are clear from the context. The density function of *F*_*X*_ is denoted by its respective lower-case letter *f*_*X*_. We call an RV *continuous*, if it takes values in IR, and *discrete*, if it takes values on a countably infinite set *X*. A *categorical* RV is one with only finitely many, say *n*, distinct outcomes. In that case, the density function can be treated as a vector $f_{X}\in {\text{I}\;\text{R}}^{n}$.

## 3 The Decision Framework

Our decision problems will concern choosing actions of minimal *loss*. Formally, if *A* is a set of actions, from which we ought to choose the best one, then a *loss-function* is usually some mapping $L:A\to {\text{I}\;\text{R}}^{+}$, so that an optimal choice from *A* is one with minimal loss under *L* (see [18] for a full-fledged treatment and theory in the context of Bayesian decision theory). In IT risk management (being used to illustrate our methods later in Section 5.2), risk is often quantified by

$$\begin{array}{c} \;\text{risk}\;=\text{damage}\times \;\text{likelihood}, \end{array}$$ (1)

which roughly resembles the idea of understanding risk as the expectation of damage. In this quantitative approach, the damage is captured by the aforementioned loss function *L*, whereas the likelihood is obtained from the distribution of the random event causing the damage.

However, losses can not always be measured precisely. For the introductory example, consider the two actions *a*_1_ = “publish the incident” and *a*_2_ = “keep the incident secret”. Either choice has unpredictable consequences so we replace the deterministic loss-function by a random variable. That is, let *a*_1_, *a*_2_ ∈ *A* be two arbitrary actions, and write *X* := *L*(*a*_1_) and *Y* := *L*(*a*_2_), respectively, for the *random* losses implied by taking these actions. The challenge now is to make a decision that minimizes the risk when losses are random.

Obviously, comparing *X* and *Y* in the way suggested by Eq (1) has some shortcomings, as it is easy to construct random variables with equal mean but highly different variance (the same issue would also exist in game theory [19], where the utility of mixed strategies is exactly the expectation of outcomes but normally disregards further moments). For the example of two Gaussian variables X∼N(5,1),Y∼N(5,10), the expectations are equal, but actions resulting in losses measured by *Y* are undesirable relative to *X*, since the fluctuation around the mean for *Y* is considerably larger than for actions with consequences described by *X*. An apparent quick fix is to take the variance into account for a decision. However, the previous issue is still not mitigated, since it is equally easy (yet only slightly more involved) to construct two random variables with equal first and second moment, but with different third moments (Example 5 will give two such distributions explicitly). Indeed, the third moment can be taken into account in the straightforward way, which has been discussed in the literature on risk attitudes; see [13, 20, 21] for a few starting references. Towards a more sophisticated approach, we will in the following use the whole object (the random variable) rather that a few representative values thereof to make a decision.

### 3.1 The Usual Stochastic Order ≤_*st*_

Choosing a best action among {*a*_1_, *a*_2_}, we ought to compare the random variables *X*, *Y* in some meaningful way. Without any further restrictions on the support or distribution, we may take the *usual stochastic order* [22] ≤_*st*_ for that purpose, which calls *X* ≤_*st*_ *Y* if and only if

$$\begin{array}{c} \text{Pr}(X>x)\leq \text{Pr}(Y>x)\;\;\text{for}\;\text{all}\;x\in (-\infty ,\infty ). \end{array}$$ (2)

Condition (2) can be stated equivalently by demanding *E*(*ϕ*(*X*)) ≤ *E*(*ϕ*(*Y*)) for all increasing functions *ϕ* for which the expectations exist (so-called test-functions). In the latter formulation, it is easy to see that, for example, the ≤_*st*_-ordering in particular entails *E*(*X*) ≤ *E*(*Y*), so that a comparison based on Eq (1) comes out the same under ≤_*st*_. Moreover, in restricting *X* and *Y* to take on only positive values, as our above definition of $L:A\to {\text{I}\;\text{R}}^{+}$ implies, *X* ≤_*st*_ *Y* implies that all moments are in pairwise ≤-order, since the respective functions *ϕ*(*x*) = *x*^*k*^ delivering them are all increasing on ${\text{I}\;\text{R}}^{+}$. Under this restriction, comparisons based on the second and third moment [20] are also covered under ≤_*st*_.

### 3.2 Generalizing ≤_*st*_: The ⪯-Ordering

In cases where it is sufficient to lower risk under an acceptance threshold, rather than truly minimizing them, we may indeed relax the ≤_*st*_-ordering in several ways: we can require Eq (2) only for large damages in (*x*_0_, ∞) for a threshold *x*_0_ that may be different for various application domains, or we may not use all increasing functions, but only a few selected ones (our construction will use the latter and entail the former relaxation). Given that moments are being used to analyze risks and are related to risk attitudes [20], let us take the functions *ϕ*(*x*) = *x*^*k*^ for k∈IN, which are all increasing on ${\text{I}\;\text{R}}^{+}$. To assure the existence of all moments *E*(*ϕ*(*X*)) < ∞ and the monotony of all members in our restricted set of test-functions, we impose the following assumptions on a general random variable *R*, which we hereafter use to quantitatively model “risk”:

**Definition 1**. Let F be the set of all random variables *R*, who satisfy the following conditions:

- *R* has a known distribution *F* with compact support (note that this implies that *R* is upper-bounded).
- *R* ≥ 1 (w.l.o.g., since as *R* is bounded, we can shift it into the region [1, ∞)).
- The probability measure induced by *F* is either discrete or continuous and has a density function *f*. For continuous random variables, the density function is assumed to be continuous.

Requirement 1 assures that all moments exist. Requirements 2 and 3 serve technical reasons that will be made clear in Lemma 2. In brief, these two assure that the ordering obtained will be total, and simplifies proofs by defining the order as equal to the natural ordering of hyperreal numbers. This will be made rigorous in Theorem 1 below. The permission to restrict our attention to moments rather than the whole random variable is given by the following well known fact:

**Lemma 1**. *Let two random variables* *X*, *Y* *have their moment generating functions* *μ*_*X*_(*s*), *μ*_*Y*_(*s*) *exist within a neighborhood* *U*_*ε*_(0). *Assume that* *m*_*X*_(*k*) := *E*(*X*^*k*^) = *E*(*Y*^*k*^) =: *m*_*Y*_(*k*) *for all* k∈IN. *Then X and Y have the same distribution*.

*Proof (Sketch)*. The proof is a simple matter of combining well-known facts about power-series and moment-generating functions (see [15] for a description).

In the following, let us write *m*_*X*_(*k*) to mean the *k*-th moment of a random variable *X*. Our next lemma establishes a total relation (so far not an ordering) between two random variables from F, on which our ordering will be based:

**Lemma 2**. *For any two probability distributions* *F*_1_, *F*_2_ *and associated random variables* *R*_1_ ∼ *F*_1_, *R*_2_ ∼ *F*_2_ *according to Definition 1, there is a* K∈IN *so that either* [∀*k* ≥ *K* : *m*_*R*_1__(*k*) ≤ *m*_*R*_2__(*k*)] *or* [∀*k* ≥ *K* : *m*_*R*_1__(*k*) ≥ *m*_*R*_2__(*k*)].

The proof of lemma 2 is given as supporting information S1 Proofs. The important fact stated here is that between any two random variables *R*_1_, *R*_2_, either a ≤ or a ≥ ordering holds *asymptotically* on the moment sequence. Hence, we can take Lemma 2 to justify the following relaxation of the usual stochastic order:

**Definition 2** (⪯-Preference Relation over Probability Distributions). Let *R*_1_, *R*_2_ ∈ F be two random variables with distribution functions *F*_1_, *F*_2_. We *prefer* *R*_1_ *over* *R*_2_, respectively the distribution *F*_1_ over *F*_2_, written as

$$\begin{array}{c} R_{1}⪯R_{2}⟺F_{1}⪯F_{2}:⟺\exists K\in \text{I}\;\text{N}\;\text{s.t.}\;\forall k\geq K:m_{R_{1}}\left(k\right)\leq m_{R_{2}}\left(k\right) \end{array}$$ (3)

*Strict preference* is denoted and defined as

$$\begin{array}{c} R_{1}≺R_{2}⟺F_{1}≺F_{2}:⟺\exists K\in \text{I}\;\text{N}\;\text{s.t.}\;\forall k\geq K:m_{R_{1}}\left(k\right)<m_{R_{2}}\left(k\right) \end{array}$$

For this definition to be a meaningful ordering, we need to show that ⪯ behaves like other orderings, say ≤ on the real numbers. We get all useful properties almost for free, by establishing an isomorphism between ⪯ and another well known ordering, namely the natural ≤ order on the hyperreal space ${}^{*}\text{I}\;\text{R}$:

**Theorem 1**. *Let* F *be according to definition 1. Assume every element* *X* ∈ F *to be represented by hyperreal number* $x={\left(E\left(X^{k}\right)\right)}_{k\in \text{I}\;\text{N}}\in {\text{I}\;\text{R}}^{\infty }/U$, *where* U *is any free ultrafilter. Let* *X*, *Y* ∈ F *be arbitrary. Then*, *X* ⪯ *Y* *if* **x** ≤ **y** *in* ${}^{*}\text{I}\;\text{R}$, *irrespectively of* U.

*Proof*. (cf. [14]) Let *F*_1_, *F*_2_ be two probability distributions, and let *R*_1_ ∼ *F*_1_, *R*_2_ ∼ *F*_2_. Lemma 2 assures the existence of some K∈IN so that *F*_1_ ⪯ *F*_2_ iff *m*_*R*_1__(*k*) ≤ *m*_*R*_2__(*k*) whenever *k* ≥ *K*. Let *L* be the set of indices where *m*_*R*_1__(*k*) ≤ *m*_*R*_2__(*k*), then complement set IN∖L is finite (it has at most *K* − 1 elements). Let U be an arbitrary free ultrafilter. Since IN∖L is finite, it cannot be contained in U as U is free. And since U is an ultrafilter, it must contain the complement a set, unless it contains the set itself. Hence, L∈U, which implies the claim.

Theorem 1 has quite some useful implications: first, the asserted independence of the ultrafilter U spares us the need to explicitly construct U (note that the general question of whether or not non-isomorphic hyperreal fields would arise from different choices of ultrafilters is still unanswered by the time of writing this article). Second, the ⪯-ordering on F inherits all properties (e.g., transitivity) of the natural ordering ≤ on ${}^{*}\text{I}\;\text{R}$, which by the transfer principle [16], hold in the same way as for ≤ on IR. More interestingly for further applications, topological properties of the hyperreals can also be transferred to F. This allows the definition of a whole game theory on top of ⪯, as was started in [17]. It must be noted, however, that the ⪯-ordering still behaves different to ≤ on IR, since, for example, the equivalence-relation induced by ⪯ does not entail an identity between distributions (since a finite number of moments is allowed to mismatch in any case).

Interestingly, although not demanded in first place, the use of moments to compare a distribution entails a similar fact as inequality Eq (2) upon which the usual stochastic order was defined:

**Theorem 2**. *Let* *X*, *Y* ∈ F *have the distributions* *F*_1_, *F*_2_. *If* *X* ⪯ *Y*, *then there exists a threshold* *x*_0_ ∈ *supp*(*F*_1_) ∪ *supp*(*F*_2_) *so that for every* *x* ≥ *x*_0_, *we have* Pr(*X* > *x*) ≤ Pr(*Y* > *x*).

The proof of this appears in the supporting information S1 Proofs. Intuitively, Theorem 2 can be rephrased into saying that:

If *F*_1_ ⪯ *F*_2_, then “extreme events” are less likely to occur under *F*_1_ than under *F*_2_.

Summarizing the results obtained, we can say that the ⪯-ordering somewhat resembles the initial definition of the usual stochastic order ≤_*st*_, up to the change of restricting the range from (−∞, ∞) to a subset of [1, ∞) and in allowing a finite number of moments to behave arbitrarily. Although this allows for an explicit disregard of the first few moments, the overall effect of choosing a ⪯-minimal distribution is shifting all the probability mass towards regions of lower damages, which is a consequence of Theorem 2. As such, this result could by itself be taken as a justification to define this ordering in first place. However, in the way developed here, the construction roots in moments and their recognized relation to risk attitudes [13, 20, 21], and in the end aligns itself to both, the intuition behind ≤_*st*_ and the focus of risk management on extreme events, without ever having stated this as a requirement to begin with. Still, by converting Theorem 2 into a definition, we could technically drop the assumption of losses being ≥1. We leave this as an aisle for future research. As a justification of the restrictions as stated, note that most risk management in the IT domain is based on categorical terms (see [23–27]), which naturally map into integer ranks ≥1. Thus, our assumption seems mild, at least for IT risk management applications (applications in other contexts like insurance [28] are not discussed here and constitute a possible reason for dropping the lower bound in future work).

### 3.3 Distributions with Unbounded Tails

Theorem 2 tells that distributions with thin tails would be preferred over those with fat tails. However, catastrophic events are usually modeled by distributions with fat, heavy or long tails. The boundedness condition in definition 1 rules out many such distributions relevant to risk management (e.g., financial risk management [29]). Thus, our next step is extending the ordering by relaxing some of the assumptions that characterize F.

The ⪯-relation cannot be extended to cover distributions with heavy tails, as those typically do not have finite moments or moment generating functions. For example, Lévi’s *α*-stable distributions [30] are not analytically expressible as densities or distribution functions, so the expression *E*(*ϕ*(*X*)) could be quite difficult to work out for the usual stochastic order. Conversely, resorting to moments, we can work with characteristic functions, which can be much more feasible in practice.

Nevertheless, such distributions are important tools in risk management. Things are, however, not drastically restricted, for at least two reasons:

1. Compactness of the support is not necessary for all moments to exist, as the Gaussian distribution has moments of all orders and is supported on the entire real line (thus violating even two of the three conditions of assumption 1). Still, it is characterized entirely by its first two moments, and thus can easily be compared in terms of the ⪯-relation.
2. Any distribution with infinite support can be approximated by a truncated distribution. Given a random variable *X* with distribution function *F*, then *truncated distribution* $\hat{F}$ is the conditional likelihood $\hat{F}\left(x\right)=\text{Pr}\left(X\leq x|a\leq X\leq b\right)$.
  By construction, the truncated distribution has the compact support [*a*, *b*]. More importantly, for a loss distribution with unbounded support [1, ∞) and given any *ε* > 0, it is easy to choose a compact interval [*a*, *b*] large enough inside [1, ∞) so that $|F\left(x\right)-\hat{F}\left(x\right)|<\varepsilon$ for all *x*. Hence, restricting ourselves to distributions with compact support, i.e., adopting assumption 1, causes no more than a numerical error that can be made as small as we wish.

More interestingly, we could attempt to play the same trick as before, and characterize a distribution with fat, heavy or long tails by a sequence of approximations to it, arising from better and better accuracy *ε* → 0. In that sense, we could hope to compare approximations rather than the true density in an attempt to extend the preference and equivalence relations ⪯ and ≡ to distributions with fat, heavy or long tails.

Unfortunately, such hope is an illusion, as a distribution is not uniquely characterized by a general sequence of approximations (i.e., we cannot formulate an equivalent to lemma 1), and the outcome of a comparison of approximations is not invariant to how the approximations are chosen (i.e., there is also no alike for lemma 2). To see the latter, take the quantile function *F*^−1^(*α*) for a distribution *F*, and consider the tail quantiles ${\bar{F}}^{-1}\left(\alpha \right)=F^{-1}\left(1-\alpha \right)$. Pick any sequence (*α*_*n*_)_*n* → ∞_ with *α*_*n*_ → 0. Since lim_*x* → ∞_ *F*(*x*) = 1, the tail quantile sequence behaves like ${\bar{F}}^{-1}\left(\alpha_{n}\right)\to \infty$, where the limit is independent of the particular sequence (*α*_*n*_)_*n* → ∞_, but only the speed of divergence is different for distinct sequences.

Now, let two distributions *F*_1_, *F*_2_ with infinite support be given. Fix two sequences *α*_*n*_ and *ω*_*n*_, both vanishing as *n* → ∞, and set

$$\begin{array}{c} a_{n}:={\bar{F}}_{1}^{-1}\left(\alpha_{n}\right)\leq b_{n}:={\bar{F}}_{2}^{-1}\left(\omega_{n}\right). \end{array}$$ (4)

Let us approximate *F*_1_ by a sequences of truncated distributions ${\hat{f}}_{1,n}$ with supports [1, *a*_*n*_] and let the sequence ${\hat{f}}_{2,n}$ approximate *f*_2_ on [1, *b*_*n*_]. Since *a*_*n*_ < *b*_*n*_ for all *n*, it is easily verified that the sequence of moments of the distributions truncated to [1, *a*_*n*_] and [1, *b*_*n*_] implies that the respective moment sequences diverge so that ${\hat{f}}_{1,n}⪯{\hat{f}}_{2,n}$ ultimately. However, by replacing the “<” by a “>” in Eq (4), we can construct approximations to *F*_1_, *F*_2_ whose truncated supports overlap one another in the reverse way, so that the approximations would always satisfy ${\hat{f}}_{1,n}⪰{\hat{f}}_{2,n}$. It follows that the sequence of approximations *cannot* be used to unambiguously compare distributions with infinite support, unless we impose some constraints on the tails of the distributions and the approximations. The next lemma (see the supporting information S1 Proofs for a proof) assumes this situation to simply not occur, which allows to give a *sufficient* condition to unambiguously extend strict preference in the way we wish.

**Lemma 3**. *Let* *F*_1_, *F*_2_ *be two distributions supported on* [1, ∞) *with continuous densities* *f*_1_, *f*_2_. *Let* ${\left(a_{n}\right)}_{n\in \text{I}\;\text{N}}$ *be an arbitrary sequence with* *a*_*n*_ → ∞ *as* *n* → ∞, *and let* ${\hat{f}}_{i,n}$ *for* *i* = 1,2 *be the truncated distribution* *f*_*i*_ *supported on* [1, *a*_*n*_].

*If there is a constant* *c* < 1 *and a value* $x_{0}\in \text{I}\;\text{R}$ *such that* *f*_1_(*x*)<*c* ⋅ *f*_2_(*x*) *for all* *x* ≥ *x*_0_, *then there is a number* *N* *such that all approximations* ${\hat{f}}_{1,n},{\hat{f}}_{2,n}$ *satisfy* ${\hat{f}}_{1,n}≺{\hat{f}}_{2,n}$ *whenever* *n* ≥ *N*.

By virtue of lemma 3, we can extend the strict preference relation to distributions that satisfy the hypothesis of the lemma but need not have compact support anymore. Precisely, we would strictly prefer one distribution over the other, if all truncated approximations are ultimately preferable over one another.

**Definition 3** (Extended Preference Relation ≺). Let *F*_1_, *F*_2_ be distribution functions of nonnegative random variables that have infinite support and continuous density functions *f*_1_, *f*_2_. We *(strictly) prefer* *F*_1_ *over* *F*_2_, denoted as *F*_1_ ≺ *F*_2_, if for every sequence *a*_*n*_ → ∞ there is an index *N* so that the approximations ${\hat{F}}_{i,n}$ for *i* = 1,2 satisfy ${\hat{F}}_{1,n}≺{\hat{F}}_{2,n}$ whenever *n* ≥ *N*.

The ≻-relation is defined alike, i.e., the ultimate preference of *F*_2_ over *F*_1_ on any sequence of approximations.

Definition 3 is motivated by the above arguments on comparability on common supports, and lemma 3 provides us with a handy criterion to decide the extended strict preference relation.

**Example 1**. It is a matter of simple algebra to verify that any two out of the three kinds of extreme value distributions (Gumbel, Frechet, Weibull) satisfy the above condition, thus are strictly preferable over one another, depending on their particular parametrization.

Definition 3 can, however, not applied to every pair of distributions, as the following example shows.

**Example 2**. Take the “Poisson-like” distributions with parameter λ > 0,

$$\begin{array}{c} f_{1}\left(k\right)\propto \{\begin{array}{cc} \frac{\lambda^{k/2}}{(k/2)!}e^{-\lambda }, & \text{when}\;k\;\text{is}\;\text{even;}\\ 0, & \text{otherwise.} \end{array},\;f_{2}\left(k\right)\propto \{\begin{array}{cc} 0, & \text{when}\;k\;\text{is}\;\text{even};\\ \frac{\lambda^{(k-1)/2}}{((k-1)/2)!}e^{-\lambda }, & \text{otherwise} \end{array} \end{array}$$

It is easy to see that no constant *c* < 1 can ever make *f*_1_ < *c* ⋅ *f*_2_ and that all moments exist. However, neither distribution is preferable over the other, since finite truncations to [1, *a*_*n*_] based on the sequence *a*_*n*_ := *n* will yield alternatingly preferable results.

An occasionally simpler condition that implies the hypothesis of definition 3 is

$$\begin{array}{c} \lim_{x\to \infty }\frac{f_{1}\left(x\right)}{f_{2}\left(x\right)}=0. \end{array}$$ (5)

The reason is simple: if the condition of definition 3 were violated, then there is an infinite sequence ${\left(x_{n}\right)}_{n\in \text{I}\;\text{N}}$ for which *f*_1_(*x*_*n*_) ≥ *c* ⋅ *f*_2_(*x*_*n*_) for all *c* < 1. In that case, there is a subsequence ${\left(x_{n_{k}}\right)}_{k\in \text{I}\;\text{N}}$ for which lim_*k* → ∞_ *f*_1_(*x*_*n*_*k*__)/*f*_2_(*x*_*n*_*k*__) ≥ *c*. Letting *c* → 1, we can construct a further subsequence of ${\left(x_{n_{k}}\right)}_{k\in \text{I}\;\text{N}}$ to exhibit that limsup_*n* → ∞_(*f*_1_(*x*_*n*_)/*f*_2_(*x*_*n*_)) = 1, so that Condition (5) would be refuted. Observe that Eq (5) is similar to the definition of a likelihood ratio order [22] in the sense that it implies both, a likelihood ratio and ≺-ordering. Note that, however, a likelihood ratio order does not necessarily imply a ≺-order, since the former only demands *f*(*t*)/*g*(*t*) to be increasing, but not a <-relation among the densities.

**Remark 1**. It must be emphasized that the above line of arguments does not provide us with a mean to extend the ⪯- or ≡-relations accordingly. For example, an attempt to define ⪯ and ≡ as above is obviously doomed to failure, as asking for two densities *f*_1_, *f*_2_ to satisfy *f*_1_(*x*) ≤ *c*_1_ ⋅ *f*_2_(*x*) ultimately (note the intentional relaxation of < towards ≤), and *f*_2_(*x*) ≤ *c*_2_ ⋅ *f*_1_(*x*) ultimately for two constants *c*_1_, *c*_2_ < 1 is nonsense.

A straightforward extension of ⪯ can be derived from (based on) the conclusion of lemma 3:

**Definition 4**. Let *F*_1_, *F*_2_ be two distributions supported on the entire nonnegative real half-line ${\text{I}\;\text{R}}^{+}$ with continuous densities *f*_1_, *f*_2_. Let ${\left(a_{n}\right)}_{n\in \text{I}\;\text{N}}$ be a diverging sequence towards ∞, and let ${\hat{F}}_{i,n}$ for *i* = 1, 2 denote the density *F*_*i*_ truncated to have support [1, *a*_*n*_]. We define *F*_1_ ⪯ *F*_2_ if and only if for every sequence ${\left(a_{n}\right)}_{n\in \text{I}\;\text{N}}$ there is some index *N* so that ${\hat{F}}_{1,n}⪯{\hat{F}}_{2,n}$ for every *n* ≥ *N*.

More compactly and informally spoken, definition 4 demands preference on all approximations with finite support except for at most finitely many exceptions near the origin.

Obviously, preference among distributions with finite support implies the extended preference relation to hold in exactly the same way (since the sequence of approximations will ultimately become constant when *a*_*n*_ overshoots the bound of the support), so definition 4 extends the ⪯-relation in this sense.

### 3.4 Comparing Distributions of Mixed Type

The representation of a distribution by the sequence of its moments is of the same form, for discrete, categorical and continuous random variables. Hence, working with sequence representations (hyperreal numbers) admits to compare continuous to discrete and categorical variables, as long as there is a meaningful common support. The framework itself, up to the results stated so far, remains unchanged and is applied to the category’s ranks instead. The ranking is then made in ascending order of loss severity, i.e., the category with lowest rank (index) should be the one with the smallest damage magnitude (examples are found in IT risk management standards like ISO 27005 [31] or the more generic ISO 31000 [32] as well as related standards).

A comparison of mixed types is, obviously, only meaningful if the respective random variables live in the same (metric) space. For example, it would be meaningless to compare ordinal to numeric data. Some applications in natural risk management define categories as numeric ranges (such as [23–26]), which *could* make a comparison of categories and numbers meaningful (but not necessarily so).

## 4 Practicalities

It must be noted that Definition 2 demands only the existence of some index after which the sequence of moment diverges, without giving any condition to assure this. Likewise, Theorem 2 is non-constructive in asserting the existence of a region onto which the ⪯-smaller distribution puts more mass than the other. Hence, practical matters of deciding and interpreting the ⪯-ordering are necessary and discussed in the following.

In general, if the two distributions are supported on the sets [1, *a*] for *F*_1_ and [1, *b*] for *F*_2_ with *b* > *a*, then the mass that *F*_2_ puts on the set (*a*, *b*] will cause the moments of *F*_2_ to grow faster than those of *F*_1_. In that case, we can thus immediately conclude *F*_1_ ⪯ *F*_2_, and we get *x*_0_ = *a* in Theorem 2. Thus, the more interesting situation arises when the supports are identical, which is assumed throughout the following subsections. Observe that it is herein not necessary to look at overlaps at the lower end of the supports, since the mass assigned near the “right end” of the support is what determines the growth of the moment sequence; the proof of Lemma 2 in the supporting information S1 Proofs more rigorously shows this.

### 4.1 Deciding ⪯ between Categorical Variables

Let *F*_1_, *F*_2_ be two distributions over a common support, i.e., a common finite set of categories, hereafter denoted in *descending* order as *c*_1_ > *c*_2_ > … > *c*_*n*_. Let ${\hat{f}}_{1}=\left(p_{1},\ldots ,p_{n}\right),{\hat{f}}_{2}=\left(q_{1},\ldots ,q_{n}\right)$ be the corresponding probability mass functions. For example, these can be normalized histograms (empirical density functions) computed from the available data to approximate the unknown distributions *F*_1_, *F*_2_ of the random variables *X*, *Y*.

Letting the category *c*_*i*_ correspond to its rank *n* − *i* + 1 within the support, it is easy to check that the expectation of *X* ∼ *F*_1_, *Y* ∼ *F*_2_ by definition is a sequence whose growth is determined by whichever distribution puts more mass on categories of high loss. Formally, if *p*_1_ > *q*_1_, then $E\left(X^{k}\right)=\sum_{j=1}^{n}p_{j}c_{j}^{k}>\sum_{j=1}^{n}q_{j}c_{j}^{k}=E\left(Y^{k}\right)$, since the growth of either sum is determined by the largest term (here being $c_{1}^{k}$). Upon the equality *p*_1_ = *q*_1_, we can retract the respective terms from both sums (as they are equal), to see whether the second-largest term $c_{2}^{k}$ tips the scale, and so on.

Overall, we end up observing that ⪯-comparing distributions is quite simple, and a special case of another common ordering relation:

**Definition 5** (lexicographic ordering). For two real-valued vectors **x** = (*x*_1_, *x*_2_, …) and **y** = (*y*_1_, *y*_2_, …) of not necessarily the same length, we define **x**<_*lex*_ **y** if and only if there is an index *i*_0_ so that *x*_*i*_0__ < *y*_*i*_0__ and *x*_*i*_ = *y*_*i*_ whenever *i* < *i*_0_.

Our discussion from above is then the mere insight that the following is true:

**Theorem 3**. *Let* *F*_1_, *F*_2_ *be two categorical random variables with a common ordered support* Ω = {*c*_1_ > *c*_2_ > … > *c*_*n*_}, *and let* **f**_1_,**f**_2_ *be the respective (empirical) density functions. Then* *F*_1_ ⪯ *F*_2_ ⇔ **f**_1_<_*lex*_ **f**_2_, *where* $f_{i}=\left(f_{i}\left(c_{1}\right),f_{i}\left(c_{2}\right),\ldots ,f_{i}\left(c_{n}\right)\right)\in {\text{I}\;\text{R}}^{n}$.

For illustration, we will apply Theorem 3 to two concrete example data sets #1 and #2 in section 5.

### 4.2 Deciding ⪯ between Continuous Variables

Let us assume that the two random variables *R*_1_ ∼ *F*_1_, *R*_2_ ∼ *F*_2_ have smooth densities *f*_1_, *f*_2_ ∈ *C*^∞^([1, *a*]) for some *a* > 1. Under this assumption, we can switch to yet another useful sequence representation:

$$\begin{array}{c} f\mapsto f={\left({\left(-1\right)}^{k}{\hat{f}}^{\left(k\right)}\left(a\right)\right)}_{k\in \text{I}\;\text{N}}. \end{array}$$ (6)

Given two distributions *f*_1_, *f*_2_ ∈ *C*^∞^, e.g., constructed from a Gaussian kernel (cf. remark 2 below), let the respective representations according to Eq (6) be **f**_1_,**f**_2_. Then, it turns out that the lexicographic ordering of **f**_1_,**f**_2_ implies the same ordering w.r.t. ⪯, or formally:

**Lemma 4** ([15]). *Let* *f*, *g* ∈ *C*^∞^([1, *a*]) *for a real value* *a* > 1 *be probability density functions. If*

$$\begin{array}{c} {\left({\left(-1\right)}^{k}\cdot f^{\left(k\right)}\left(a\right)\right)}_{k\in \text{I}\;\text{N}}{<}_{lex}{\left({\left(-1\right)}^{k}\cdot g^{\left(k\right)}\left(a\right)\right)}_{k\in \text{I}\;\text{N}}, \end{array}$$

*then* *f* ⪯ *g*.

Lemma 4 will be demonstrated on our example data set #3, in connection with a kernel density estimate, in section 5. Practically, we can thus decide the ⪯-relation by numerically computing derivatives of increasing order, until the decision is made by the lexicographic ordering (which, for our experiments, happened already at zeroth order in many cases).

**Remark 2**. The assumption on differentiability is indeed mild, as we can cast any integrable density function into a *C*^∞^-function by convolution with a Gaussian density *k*_*h*_ with zero mean and variance *h*. Clearly, *f* ∗ *k*_*h*_ ∈ *C*^∞^ by the differentiation theorem of convolution. Moreover, letting *h* → 0, we even have *L*^1^-convergence of *f* ∗ *k*_*h*_ → *f*, so that the approximation can be made arbitrarily accurate by choosing the parameter *h* > 0 sufficiently small. Practically, when the distributions are constructed from empirical data, the convolution corresponds to a kernel density estimation (i.e., a standard nonparametric distribution model). Using a Gaussian kernel then has the additional appeal of admitting a closed form of the *k*-th derivatives (*f* ∗ *k*_*h*_)^(*k*)^, involving Hermite-polynomials.

#### Observation – “⪯ ≈ <_*lex*_”

As an intermediate résumé, the following can be said:

Under a “proper” representation of the distribution (histogram or continuous kernel density estimate), the ⪯-order can be decided as a humble lexicographic order.

This greatly simplifies matters of practically working with ⪯-preferences, and also fits into the intuitive understanding of risk and its formal capture by theorem 2: *whichever distribution puts more mass on far-out regions is less favourable under ⪯.*

### 4.3 Comparing Deterministic to Random Effects

In certain occasions, the consequence of an action may result in perfectly foreseeable effects, such as fines or similar. Such deterministic outcomes can be modeled as degenerate distributions (point- or Dirac-masses). These are singular and thus outside F by Definition 1. Note that the canonic embedding of the reals within the hyperreals represents a number a∈IR by the constant sequence (*a*, *a*, …). Picking up this idea would be critically flawed in our setting, as any such constant sequence would be preferred over any probability distribution (whose moment sequence diverges and thus overshoots *a* inevitably and ultimately).

However, it is easy to work out the moment sequence of the constant *X* = *a* as *E*(*X*^*k*^) = *E*(*a*^*k*^) = *a*^*k*^ for all k∈IN. In this form, the ⪯-relation between the number *a* and the continuous random variable *Y* supported on Ω = [1, *b*] can be decided as follows:

1. If *a* < *b*, then *a* ⪯ *Y*: to see this, choose *ε* < (*b* − *a*)/3 so that *f* is strictly positive on a compact set [*b* − *ε*, *b* − 2*ε*] (note that such a set must exist as *f* is continuous and the support ranges until *b*). We can lower-bound the *k*-th moment of *Y* as
  $$\begin{array}{ccc} \int_{1}^{b}y^{k}f\left(y\right)dy & \geq & (\underset{\left[b-2\varepsilon ,b-\varepsilon \right]}{\inf}f)\cdot \int_{b-2\varepsilon }^{b-\varepsilon }y^{k}dy\\ & = & \frac{1}{k+1}[{\left(b-\varepsilon \right)}^{k+1}-{\left(b-2\varepsilon \right)}^{k+1}]. \end{array}$$
  Note that the infimum is positive as *f* is strictly positive on the compact set [*b* − 2*ε*, *b* − *ε*]. The lower bound is essentially an exponential function to a base larger than *a*, since *b* − 2*ε* > *a*, and thus (ultimately) grows faster than *a*^*k*^.
2. If *a* > *b*, then *Y* ⪯ *a*, since *Y* – in any possible realization – leads to strictly less damage than *a*. The formal argument is now based on an upper bound to the moments, which can be derived as follows:
  $$\begin{array}{cc} \int_{1}^{b}y^{k}f\left(y\right)dy & \leq \left(\underset{\left[1,b\right]}{\sup}f\right)\cdot \int_{1}^{b}y^{k}dy=\left(\underset{\left[1,b\right]}{\sup}f\right)\frac{1}{k+1}b^{k+1}. \end{array}$$
  It is easy to see that for *k* → ∞, this function grows slower than *a*^*k*^ as *a* > *b*, which leads to the claimed ⪯-relation.
3. If *a* = *b*, then we apply the mean-value theorem to the integral occurring in $E\left(Y^{k}\right)=\int_{1}^{a}y^{k}f\left(y\right)dy$ to obtain an *ξ* ∈ [1, *a*] for which
  $$\begin{array}{c} E\left(Y^{k}\right)=\xi^{k}\underset{=1}{\underset{︸}{\int_{1}^{a}f\left(y\right)dy}}=\xi^{k}\leq a^{k} \end{array}$$
  for all *k*. Hence, *Y* ⪯ *a* in that case. An intuitive explanation stems from the fact that *Y* may assign positive likelihood to events with less damage as *a*, whereas a deterministic outcome is always larger or equal to anything that *Y* can deliver.

### 4.4 On the Interpretation of ⪯ and Inference

The practical meaning of the ⪯-preference is more involved than just a matter of comparing the first few moments. Indeed, unlike for IT risk preferences based on Eq (1), the first moment can be left unconstrained while ⪯ may still hold in either direction.

For general inference, the comparison of two distributions provides a necessary basis (i.e., to define optimality, etc.). For example, (Bayesian) decision theory or game theoretic models can be defined upon ⪯, via a much deeper exploration of the embedding of F into the hyperreals (by mapping a distribution to its moment sequence), such as the induced topology and calculus based on it. In any case, however, we note that the previous results may help in handling practical matters of ⪯ inside a more sophisticated statistical decision or general inference process. For practical decisions, some information can be obtained from the value *x*_0_ that Theorem 2 speaks about. This helps assessing the meaning of the order, although the practical consequences implied by ≤_*st*_ or ⪯ are somewhat similar. The main difference is Eq (2) holding only for values ≥*x*_0_ in case of ⪯. The threshold can hence be found by numerically searching for the largest (“right-most”) intersection point of the respective survival functions; that is, for two distributions *F*_1_ ⪯ *F*_2_, a valid *x*_0_ in Theorem 2 is any value for which 1 − *F*_1_(*x*) ≤ 1 − *F*_2_(*x*) for all *x* ≥ *x*_0_. An approximation of *x*_0_, e.g., computed by a bisective search in common support of both distributions, then more accurately describes the “statistically best” among the available actions, since losses >*x*_0_ are more likely for all other options. A practical decision, or more general inference based on ⪯, should therefore be made upon computing *x*_0_ as an explicit auxiliary information, in order to assign a quantitative meaning to “extreme events” in the interpretation underneath Theorem 2. Further issues of practical decision making in the context of IT risk management are discussed along the first empirical example found in section 5.2.

Section 5 will not discuss (statistical) inference since the details are beyond the scope of this work (we leave this to follow up work). Instead, the following section will be dedicated to numerical illustrations of ⪯ only, without assigning any decisional meaning to the ⪯-preferred distributions. For each example, we will also give an approximation (not the optimal) value of *x*_0_.

## 5 Numerical Examples

Let us now apply the proposed framework to the problem of comparing effects that are empirically measurable, when the precise action/response dynamics is unknown. We start by looking at some concrete parametric models of extreme value distributions first, to exemplify cases of numerical comparisons of distributions with unbounded tails in Section 5.1.

In Section 5.3, we will describe a step-by-step evaluation of our ⪯-ordering on empirical distributions. The sources and context of the underlying empirical data sets are described in section 5.2. From the data, we will compile non-parametric distribution models, which are either normalized histograms or kernel density estimators. On these, we will show how to decide the ⪯-relation using the results from section 4.

### 5.1 Comparing Parametric Models

We skip the messy algebra tied to the verification of the criteria in Section 3.3, and instead compute the moments numerically to illustrate the growth/divergence of moment sequences as implied by Lemma 2.

**Example 3** (different mean, same variance). Consider two Gumbel-distributions *X* ∼ *F*_1_ = *Gumbel*(31.0063, 1.74346) and *Y* ∼ *F*_2_ = *Gumbel*(32.0063, 1.74346), where a density for *Gumbel*(*a*, *b*) is given by

$$\begin{array}{c} f\left(x|a,b\right)=\frac{1}{b}e^{\frac{x-a}{b}-e^{\frac{x-a}{b}}}, \end{array}$$

where a∈IR and *b* > 0 are the location and scale parameter.

**Fig 1:**
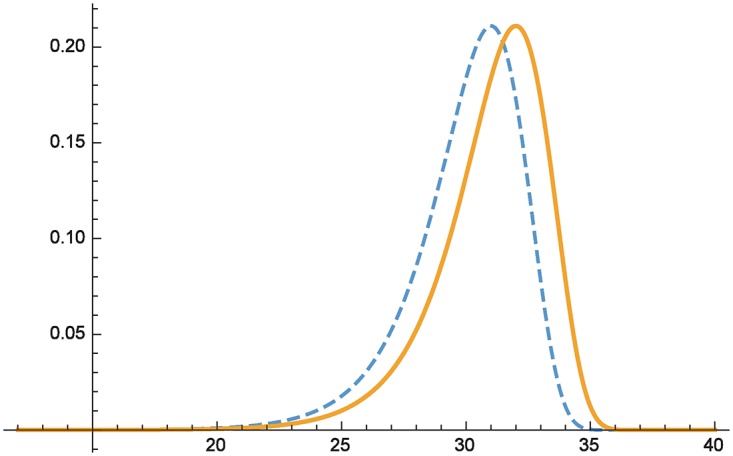
Comparing distributions with different means.

Computations reveal that under the given parameters, the means are *E*(*X*) = 30, *E*(*Y*) = 31 and *Var*(*X*) = *Var*(*Y*) = 5. Fig 1 plots the respective densities of *F*_1_ (dashed) and *F*_2_ (solid line). The respective moment sequences evaluate to

$$\begin{array}{ccc} E(X^{k}) & = & (30,905,27437.3,835606,2.55545\times 10^{7},\ldots ),\\ E(Y^{k}) & = & (31,966,30243.3,950906,3.00162\times 10^{7},\ldots ), \end{array}$$

thus illustrating that *F*_1_ ⪯ *F*_2_. This is consistent with the intuition that the preferred distribution gives *less expected damage*. The concrete region about which Theorem 2 speaks is at least for damages >*x*_0_ = 25 (cf. Theorem 2).

**Example 4** (same mean, different variance). Let us now consider two Gumbel-distributions *X* ∼ *F*_1_ = *Gumbel*(6.27294, 2.20532) and *Y* ∼ *F*_2_ = *Gumbel*(6.19073, 2.06288), for which *E*(*X*) = *E*(*Y*) = 5 but *Var*(*X*) = 8 > *Var*(*Y*) = 7.

**Fig 2:**
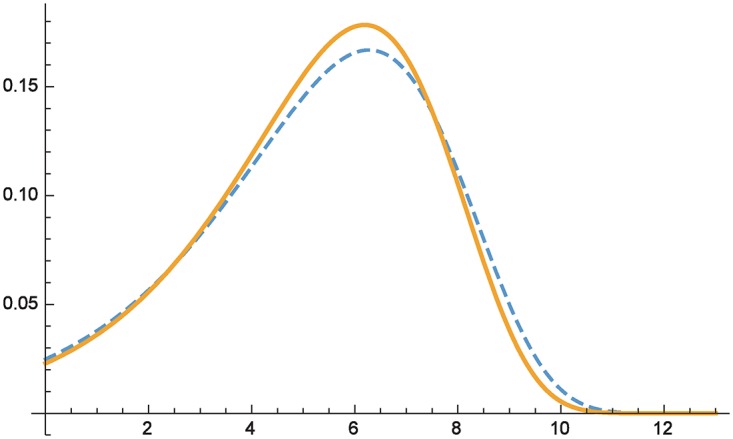
Comparing distributions with equal means but different variance.

Fig 2 plots the respective densities of *F*_1_ (dashed) and *F*_2_ (solid line). The respective moment sequences evaluate to

$$\begin{array}{ccc} E(X^{k}) & = & (5,33,219.215,1654.9,11957.8,\ldots ),\\ E(Y^{k}) & = & (5,32,208.895,1517.51,10806.8,\ldots ), \end{array}$$

thus illustrating that *F*_2_ ⪯ *F*_1_. This is consistent with the intuition that among two actions leading to the same expected loss, the preferred one would be one for which the variation around the mean is smaller; thus the loss prediction is “more stable”. The range on which damages under *F*_2_ are less likely than under *F*_1_ begins at *x* > *x*_0_ ≈ 5.5 (cf. Theorem 2).

**Example 5** (different distributions, same mean and variance). Let us now consider a situation in which the expected loss (first moment) and variation around the mean (second moment) are equal, but the distributions are different in terms of their shape. Specifically, let *X* ∼ *F*_1_ = *Gamma*(260.345, 0.0373929) and *Y* ∼ *Weibull*(20, 10), with densities as follows:

$$\begin{array}{c} f_{\text{Gamma}}\left(x|a,b\right)=\{\begin{array}{cc} \frac{b^{-a}x^{a-1}e^{-\frac{x}{b}}}{\Gamma (a)}, & x>0;\\ 0, & \text{otherwise} \end{array} \end{array}$$

$$\begin{array}{c} f_{\text{Weibull}}\left(x|a,b\right)=\{\begin{array}{cc} \frac{ae^{-{\left(\frac{x}{b}\right)}^{a}}{\left(\frac{x}{b}\right)}^{a-1}}{b}, & x>0;\\ 0, & \text{otherwise} \end{array} \end{array}$$

**Fig 3:**
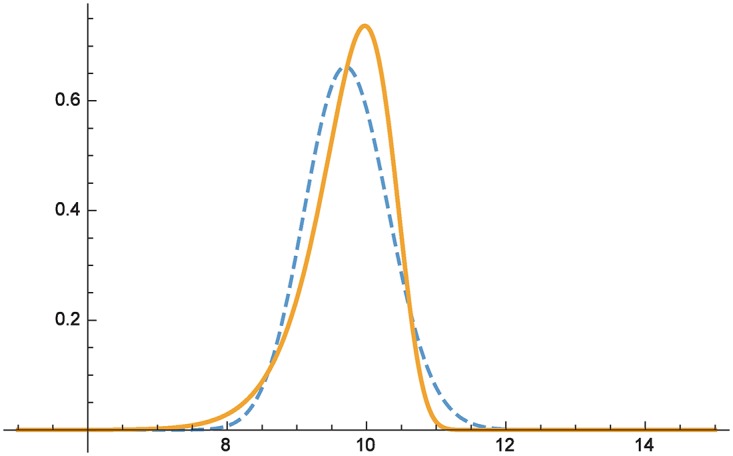
Comparing distributions with matching first two moments but different shapes.

Fig 3 plots the respective densities of *F*_1_ (dashed) and *F*_2_ (solid line). The respective moment sequences evaluate to

$$\begin{array}{ccc} E(X^{k}) & = & (9.73504,95.1351,933.259,9190.01,90839.7,\ldots ),\\ E(Y^{k}) & = & (9.73504,95.1351,933.041,9181.69,90640.2,\ldots ), \end{array}$$

thus illustrating that *F*_2_ ⪯ *F*_1_. In this case, going with the distribution that visually “leans more towards lower damages” would be flawed, since *F*_1_ nonetheless assigns larger likelihood to larger damages. The moment sequence, on the contrary, unambiguously points out *F*_2_ as the preferred distribution (the third moment tips the scale here; cf. [13, 20, 21]). The statistical assurance entailed by Theorem 2 about an interval in which high damage incidents are less likely (at least) includes losses >*x*_0_ ≈ 10.3.

### 5.2 Empirical Test Data and Methodology

To demonstrate how the practical matters of comparing distributions work, we will use three sets of empirical data, based on qualitative data from risk estimation, and based on simulating a malware outbreak using percolation.

#### Test Data Set #1 – IT Risk Assessments

The common quantitative understanding of risk by the Formula (1) is easily recognized as the *expectation* (i.e., first moment) of a loss distribution. Although being standard in quantitative IT risk management, its use is discouraged by the German Federal Office of Information Security (BSI) [33] for several reasons besides the shortcomings that we discussed here (for example, statistical data may be unavailable at the desired precision and an exact formula like Eq (1) may create the illusion of accuracy where there is none [33]).

Best practices in risk management (ranging up to norms like the ISO27005 [31], the ISO31000 [32] or the OCTAVE Allegro framework [34]) usually recommend the use of *qualitative* risk scales. That is, the expert is only asked to utter an opinion about the risk being “low/medium/high” or perhaps using a slightly more fine-grained but in any case ordinal scale. In a slight abuse of formalism, these categories are then still carried into an evaluation of Eq (1) (cf. [35]) towards finding the decision with the “least” risk in qualitative terms as Eq (1) gives.

Categorical risk assessments are heavily used in the IT domain due to their good systematization and tool support. Our first test data set is thus a risk assessment made in terms of the Common Vulnerability Scoring System (CVSS) [27]. The CVSS ranks risks on a scale from 0 to 10, as a decimal rounded up to one place behind the comma. Usually, these CVSS values come from domain experts, so there is an intrinsic ambiguity in the opinions on grounds of which a decision shall be made. Table 1, taken from [36] (by kind permission of the author A. Beck), shows an example of such expert data for two security system installments being assessed by experts in the left and right part of the table (separated by the double vertical line). The ⪯-ordering shall now help to choose the better of the two options, based on the ambiguous and even inconsistent domain expert inputs. For simplicity of the example, we did not work with the fine-grained CVSS scores, but coarsened them into three categories, i.e., intervals of scores *low* = [0, 3) (L), *medium* = [4, 8) (M) and *high* = [8, 10] (H). We remark that the categorial assessment was added in this work, and is not from the source literature.

**Table 1:** Example CVSS Risk Assessment [36].

Expert (anonymized)	CN-863	ER-881	ÖL-968	BA-576	RC-813	RR-745	EN-720	EF-375	UE-941	RI-740	UM-330	TR-790	EE-677	ER-640	EE-489
Scenario	1	1	1	1	1	1	2	2	2	2	2	2	2	2	2
CVSS	10	6.4	9	7.9	7.1	9	10	7.9	8.2	7.4	10	8.5	9	9	8.7
Risk	H	M	H	M	M	H	H	M	H	M	H	H	H	H	H

#### Test Data Set #2 – Malware Outbreaks

Computer malware infections are continuously reported in the news, with an early and prominent example having been the Stuxnet worm in 2008 [37], which infected the Iranian uranium enrichment facilities. Ever since, the control and supervision of cyber-physical systems has gained much importance in risk management, since attacks on the computer infrastructure may have wide effects ranging up to critical supply infrastructures such as water supply, power supply, and many others (e.g., oil, gas or food supply networks, etc.).

The general stealthiness of such infections makes an exact assessment of risk difficult. A good approach to estimate that risk is to apply outbreak simulation models, such as, for example, using percolation theory [38, 39]. These simulations provide us with possible infection scenarios, in which the number of infected nodes (after a fixed period of time), can be averaged into a probability distribution describing the outcome of an infection. Repeating the simulation with different system configurations yields various outcome distributions. An example for a network with 20 nodes and 1000 repetitions per simulation is displayed in Table 2. The ⪯-relation shall then help deciding which configuration is better in minimizing the risk of a large outbreak.

**Table 2:** Simulated malware infection.

size *n* of the outbreak	config. 1	config. 2
1	0	0
2	1	0
3	0	0
4	3	0
5	5	3
6	1	4
7	4	6
8	5	5
9	9	8
10	6	25
11	13	33
12	22	39
13	29	85
14	44	131
15	86	160
16	135	164
17	182	150
18	245	113
19	173	64
20	37	10

Data: number of occurrences of an outbreak of size *n* under two configurations, after a fixed time period [39]

#### Test Data Set #3 – Nile Water Level

As a third data set, we use one that ships with the statistical software suite R. Concretely, we will look at the dataset Nile that consists of the measurements of the annual flow of the Nile river between 1871 and 1970. For comparisons, we will divide the data into two groups of 50 observations each (corresponding to years). The decision problem associated with it is the question of which period was more severe in terms of water level. Extending the decision problem to more than two periods would then mean searching for a *trend* within the data. Unlike a numerical trend, such as a sliding mean, we would here have a “sliding empirical distribution” to determine the trend in terms of randomness.

### 5.3 Comparing Empirical Distributions

With the three data sets as described, let us now look into how decisions based on the empirical data can be made.

#### Categorical Data – Comparing Normalized Histograms

**Fig 4:**
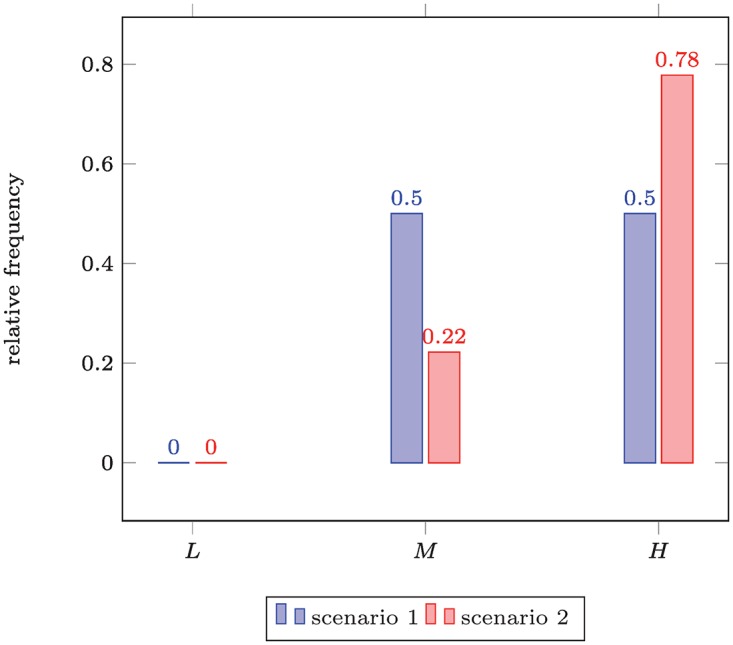
Comparing Empirical Distributions – Test Data #1 in Nominal Scale. Outcome: “scenario 1” ≺ “scenario 2” (i.e., scenario 1 has lower security risk, based on the coarsened data), for medium and high categories (*x*_0_ = ‘*M*’).

Compiling an empirical distribution from the example CVSS data in Table 1 gives the histograms shown in Fig 4. Clearly, scenario 1 is preferable here, as it is less frequently rated with high damage than scenario 2. On the contrary, the decision is much less informed than in the case where the full numeric data would have been used. We will thus revisit this example later again.

**Fig 5:**
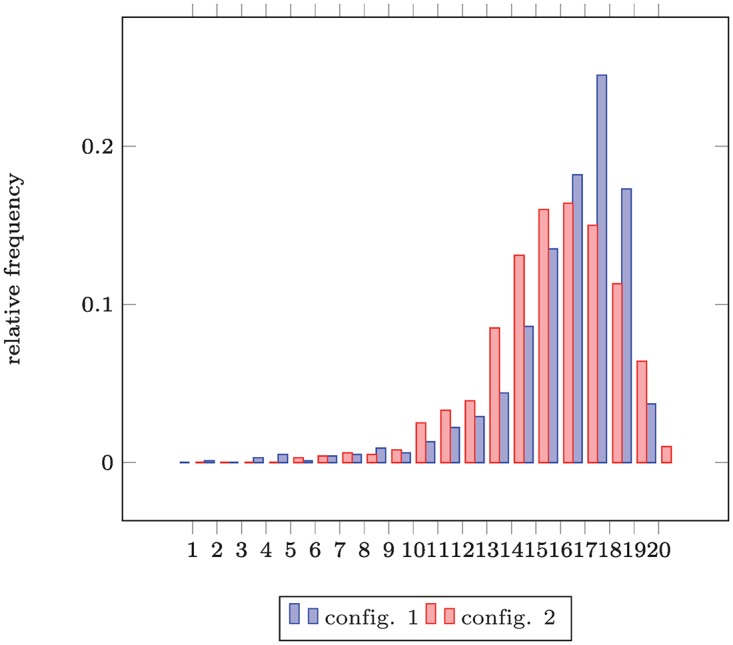
Test Data #2 – (Simulated) Empirical Distribution of Malware Outbreak Sizes under two Configurations. Outcome: “config. 2” ≺ “config. 1” (i.e., the second configuration is more secure w.r.t. extreme outcomes, i.e., infections of >*x*_0_ = 9 nodes).

For the simulated malware infection data in Table 2, the empirical distribution of the number of affected nodes as shown in Fig 5 is obtained by normalization of the corresponding histograms. In this case, configuration 2 is preferable as the maximal damage of 20 node has occurred less often than in configuration 1.

#### Continuous Data – Comparing Kernel Density Estimates

If the data itself is known to be continuous, then a nonparametric distribution estimate can be used to approximate the unknown distribution.

In the following, let us write $\hat{f}$ to mean a general kernel density estimate based on the data (observations) *x*_1_, …, *x*_*n*_, of the form

$$\begin{array}{c} {\hat{f}}_{n}\left(x\right)=\frac{1}{n\cdot h}\sum_{i=1}^{n}K(\frac{x-x_{i}}{h}), \end{array}$$ (7)

where *K*(*x*) is the chosen kernel function, and *h* > 0 is a bandwidth parameter, whose choice is up to any (of many existing) heuristics (see [40, 41] among others). Computing a kernel density estimate from data is most conveniently done by invoking the density command within the R statistical computing software [42].

**Fig 6:**
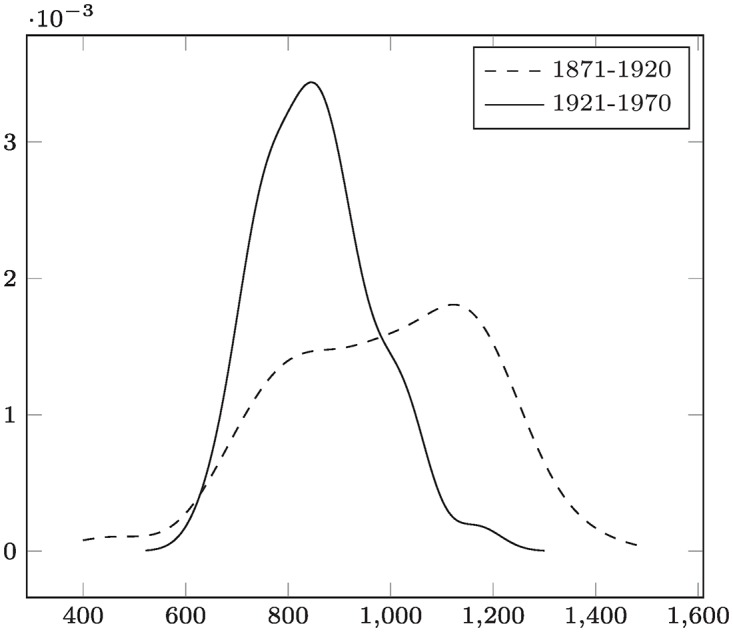
Comparison of two kernel density estimates – Test Data #3 (Nile Water Level). Outcome: “1921-1970” ≺ “1871-1920” (indicating that floodings at a water level >*x*_0_ ≈ 220 (Theorem 2) have been more likely in the years before 1921).

This comparison of two kernel density estimates (KDE) is illustrated in Fig 6. For that purpose, we divided the test dataset #3 (data(Nile) in R) into observations covering the years 1871-1920 and 1921-1970. For both sets the density is estimated with a Gaussian kernel and the default bandwidth choice nrd0 (Silverman’s rule [41]) yielding a KDE ${\hat{f}}_{1}$ with bandwidth *h*_1_ = 79.32 for the years 1871-1920 and a KDE ${\hat{f}}_{2}$ with bandwidth *h*_2_ = 45.28 for the years 1921-1970. Further we have the maximal observed values *x*_*n*_1__ = 1370 and *y*_*n*_2__ = 1170, and see that

$$\begin{array}{c} x_{n_{1}}+h_{1}=1449.32>y_{n_{2}}+h_{2}=1215.28. \end{array}$$

Therefore (and also by visual inspection of Fig 6), the density for the period from 1871 until 1920 has had higher likelihoods for a high water level, which became less in the period from 1921-1970, thus indicating a “down-trend” by ${\hat{f}}_{2}≺{\hat{f}}_{1}$.

#### Using Gaussian Kernels

Commonly, the kernel density approximation is constructed using Gaussian kernels per default, in which case *K* takes the form $K\left(x\right)=\frac{1}{\sqrt{2\pi }}\exp\left(-\frac{1}{2}x^{2}\right)$. Definition 1 is clearly not met, but there is also no immediate need to resort to the extended version of ⪯ as given by Definition 4. Indeed, if we simply truncate the KDE at any point *a* > 1 into the distribution $\hat{f}$ and remember that *K* ∈ *C*^∞^, the truncated kernel density estimate is again a *C*^∞^-density, as required by Lemma 4.

**Fig 7:**
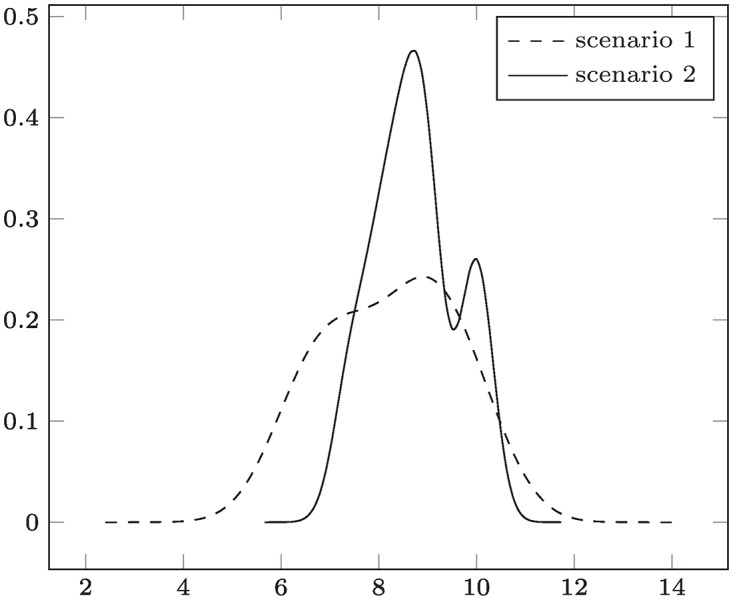
Comparing Nonparametric Distribution Models – Test Data #1 (directly used). Outcome: “scenario 2” ≺ “scenario 1” (given a more refined view that in Fig 4, scenario 1 has less security).

Returning to the CVSS example data in Table 1, we constructed Gaussian kernel density estimates *f*_1_, *f*_2_, with bandwidths *h*_1_ ≈ 0.798 and *h*_2_ ≈ 0.346 (using the default Silverman’s rule in R); plots of which are given in Fig 7. Using the criterion of Lemma 4 in connection with the lexicographic ordering, we end up finding that *f*_2_ ⪯ *f*_1_, in contrast to our previous finding. This is, however, only an inconsistency at first glance, and nevertheless intuitively meaningful if we consider the context of the decision and the effect of the nonparametric estimation more closely:

- Since scenario 2 is based on more data than scenario 1, the bandwidth *h*_2_ is less than *h*_1_. This has the effect of the distribution being “more condensed” around higher categories, as opposed to the distribution for scenario 1, whose tail is much thicker. Consequently, the decision is to prefer scenario 2 is implicitly based on the larger data set, and considers the higher uncertainty in the information about scenario 1.
- The Gaussian kernel has tails reaching out to +∞, which also assigns positive mass to values outside the natural range of the input data (1…10 in case of CVSS). In many contexts, observations may not be exhaustive for the possible range (e.g., monetary loss up to the theoretical maximum may – hopefully – not have occurred in a risk management process in the past). By construction, the KDE puts more mass on the tails the more data in this region is available. From a security perspective, this mass corresponds to *zero-day exploit events*. Thus, such incidents are automatically accounted for by ⪯.

## 6 Discussion

Our proposed preference relation is designed for IT risk management. In this context, decision makers often rely on scarce and purely subjective data coming from different experts. Bayesian techniques that need large amounts of data are therefore hard to apply (and somewhat ironically, a primary goal of IT risk management is exactly minimizing the lot of incidents that could deliver the data). Since the available information may not only be vague but possibly also inconsistent, consensus finding by data aggregation is often necessary. There exist various non-probabilistic methods to do this (such as fuzzy logic, Dempster-Shafer theory, neural networks, etc.) and perform extremely well in practice, but the interpretation of the underlying concepts is intricate and the relation to values and business assets is not trivial.

To retain interpretability, data aggregation often means averaging (or taking the median of) the available risk figures, in order to single out an optimal action. This clearly comes at the cost of losing some information. Stochastic orders elegantly tackle the above issues by letting the entire data go into a probability distribution (and thus preserving all information), and defining a meaningful ranking on the resulting objects. However, not all stochastic orders are equally meaningful for the peculiarities of IT risk management. For example, low damages are normally disregarded as being covered by the system’s natural resilience, i.e., no additional efforts are put on lowering a risk that is considered as low already. The relevance of risks depends on whether or not a certain acceptable damage threshold is exceeded. IT risk managers typically care about significant (extreme) distortions and events with high potential of damage but with only a limited lot of reported evidence so far, such as zero day exploits or advanced persistent threats.

Consequently, a suitable ordering may reasonably ignore damages of low magnitude, and focus on extreme outcomes, i.e., the tails of the respective loss distributions. This is a major reason for our transition from the usual stochastic order that takes into account the entire loss range (in fact all IR, according to Eq (2)) to one that explicitly focuses on a left neighborhood of the loss maximum. In a converse approach to the same problem, this could as well be used as a starting point to define an order, but starting from moments instead and finishing with an ordering that is about the heaviness of tails is an interesting lesson learned from our proposed technique of using ${}^{*}\text{I}\;\text{R}$ to construct the ordering here. More importantly, the rich structure of ${}^{*}\text{I}\;\text{R}$, being available without additional labor, makes our ordering useable with optimization and game theory, so that important matters of security economics can be covered as a by-product. This non-standard technique of constructing an ordering is an independent contribution of this work.

Summarizing our point, decision making based on a stochastic ordering has the appeal of a statistical fundament that is easy to communicate and, more importantly, fits well into existing risk management standards (ISO 27000, ISO 31000, etc.).

**Outlook**: The well defined arithmetic over ${}^{*}\text{I}\;\text{R}$, into which Theorem 1 embeds the (risk) distribution models in F, lets us technically work with distributions like as if we were in a topological field. This embedding offers an interesting unexplored (and nontrivial) route of future research: though the operations on random variables (say, addition or quotients) do not correspond to the same operations in ${}^{*}\text{I}\;\text{R}$ (which is immediately evident from the definition), many other operations and even functions of random variables can be studied in the space ${}^{*}\text{I}\;\text{R}$ rather than on the set of distributions. So we can, for example, do optimization theory over distributions but equipped with the full armory of calculus known from the reals (that analogously holds in the space ${}^{*}\text{I}\;\text{R}$ by virtue of Łos’ theorem or the transfer principle [16]).

Our ordering relation on the set of probability distributions can be extended towards a theory of games on these spaces (this extension is based on the topology that the order induces, upon which Nash’s result on the existence of equilibria can be re-established on our space of probability distributions). First steps into applying the framework to competitive decision-situations have been taken in [17], and will be further detailed in follow up research articles.

## Supporting Information

S1 Proofs Proof of Lemma 2, Theorem 2 and Lemma 3. The formal arguments provided here first appeared in [14], and are repeated for the sake of completeness and convenience of the reader.
(PDF)

S1 File For an extended discussion of the mathematical background, see the preliminary research reports [14, 15]. Both are available as supporting information to this article.
(ZIP)
