# Correction: Decisions with Uncertain Consequences—A Total Ordering on Loss-Distributions

**DOI:** 10.1371/journal.pone.0333014

**Published:** 2025-09-22

**Authors:** Stefan Rass, Sandra König, Stefan Schauer

In the ‘Generalizing ≤_st_: The ≼-Ordering’ subsection of ‘The Decision Framework’, there is an error in Lemma 2, Theorem 1 and Theorem 2, and additional hypotheses are included.

For Lemma 2, the correct hypothesis is: *For any two probability distributions F*_1_, *F*_2_ *and associated random variables R*_1_ ∼ *F*_1_, *R*_2_ ∼ *F*_2_*, according to Definition 1. Let F*_*1*_*, F*_*2*_ **be jointly supported on a compact set* Ω=*[0,a] and let the respective density functions f**_*1*_*, f*_*2*_ *satisfy f*_*1*_*(a)≠f*_*2*_*(a). Then, there exists a number* K∈N **so that either* [∀*k* *≥* *K*:* m_R*1*_*(*k*) ≤* m_R*2*_*(*k**)] or* [∀*k* *≥* *K*:* m_R*1*_*(*k*) ≥* m_R*2*_*(*k*)].*

For Theorem 1, the correct hypothesis is: *Let* F *be according to definition 1. Assume every element X* ∈ F *to be represented by hyperreal number* $x={\left(E\left(X^{i}\right)\right)}_{i\in N},x\in R^{\infty }/U$*, where* U *is any free ultrafilter. Let X*, *Y* ∈ F *be arbitrary, and satisfying the hypothesis of Lemma 2 with* $\Omega =\text{supp}\left(F_{\text{1}}\right)\cup \text{supp}\left(F_{\text{2}}\right)$*. Then*, *X* ≼ *Y if* **x** ≤ **y** *in* $\begin{array}{c} {}*\;R \end{array}$, *irrespectively of* U.

For Theorem 2, the correct hypothesis is: *Let X*, *Y* ∈ F *have the distributions F*_*1*_*, F*_*2*_ *satisfying the hypothesis of Lemma 2. If X *≼ *Y, then there exists a threshold* $x_{0}\in \text{supp}\left(F_{1}\right)\cup \text{supp}\left(F_{2}\right)$ *so that for every* $x\geq x_{0}$*, we have* Pr(X>x)≤Pr(Y>x)*.*

S1 File is uploaded incorrectly. Please view the correct S1 File below.

## Supporting information

S1 File (ZIP)
