# Supplementary material for: Decisions with Uncertain Consequences—A Total Ordering on Loss-Distributions
Source: PLoS One. 2016 Dec 28;11(12):e0168583. doi: 10.1371/journal.pone.0168583 (PMC5193423; doi:10.1371/journal.pone.0168583)
Supplement: S1 Proofs — The formal arguments provided here first appeared in [14], and are repeated for the sake of completeness and convenience of the reader. (PDF) [file pone.0168583.s001.pdf]

S1 Proofs

S1.1 Proof of Lemma 2

We first discuss the continuous case, which illustrates the basic idea that can be applied alike to categorical and discrete distributions.

Let f_1, f_2 denote the densities of the distributions F_1, F_2. Fix the smallest $b^* > 1$ so that $\Omega := [1, b^*]$ covers both the supports of F_1 and F_2. Consider the difference of the k-th moments, given by

$$\begin{aligned}\Delta(k) := \mathrm{E}\big(R_1^k\big) - \mathrm{E}\big(R_2^k\big) &= \int_\Omega x^k f_1(x)dx - \int_\Omega x^k f_2(x)dx \\ &= \int_\Omega x^k (f_1 - f_2)(x)dx. \qquad (1)\end{aligned}$$

Towards a lower bound to (1), we distinguish two cases:

1. If $f_1(x) > f_2(x)$ for all $x \in \Omega$, then $(f_1 - f_2)(x) > 0$ and because f_1, f_2 are continuous, their difference attains a minimum $\lambda_2 > 0$ on the compact set Ω. So, we can lower-bound (1) as $\Delta(k) \geq \lambda_2 \int_\Omega x^k dx \to +\infty$, as $k \to \infty$.

2. Otherwise, we look at the right end of the interval Ω, and define

   $$a^* := \inf\{x \geq 1 : f_1(x) > f_2(x)\}.$$

   Without loss of generality, we may assume $a^* < b^*$. To see this, note that if $f_1(b^*) \neq f_2(b^*)$, then the continuity of $f_1 - f_2$ implies $f_1(x) \neq f_2(x)$ within a range $(b^* - \varepsilon, b^*]$ for some $\varepsilon > 0$, and a^* is the supremum of all these ε. Otherwise, if $f_1(x) = f_2(x)$ on an entire interval $[b^* - \varepsilon, b^*]$ for some $\varepsilon > 0$, then $f_1 \not> f_2$ on Ω (the opposite of the previous case) implies the existence of some $\xi < b^*$ so that $f_1(x) < f_2(x)$, and a^* is the supremum of all these ξ (see Fig A for an illustration). In case that $\xi = 0$, we would have $f_1 \geq f_2$ on Ω, which is either trivial (as $\Delta(k) = 0$ for all k if $f_1 = f_2$) or otherwise covered by the previous case.

   In either situation, we can fix a compact interval $[a, b] \subset (a^*, b^*) \subset [1, b^*] = \Omega$ and two constants $\lambda_1, \lambda_2 > 0$ (which exist because f_1, f_2 are bounded as being continuous on the compact set Ω), so that the function

   $$\ell(k, x) := \begin{cases} -\lambda_1 x^k, & \text{if } 1 \leq x < a; \\ \lambda_2 x^k, & \text{if } a \leq x \leq b. \end{cases}$$

   lower-bounds the difference of densities in (1) (see Fig A), and

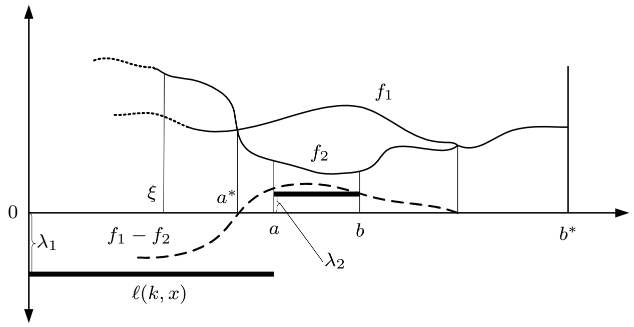

Figure A. Lower-bounding the difference of densities

$$\Delta(k) = \int_1^{b^*} x^k (f_1 - f_2)(x)dx \geq \int_1^b \ell(x,k)dx$$
$$= -\lambda_1 \int_1^a x^k dx + \lambda_2 \int_a^b x^k dx$$
$$= -\frac{a^{k+1}}{k+1}(\lambda_1 + \lambda_2) + \lambda_2 \frac{b^{k+1}}{k+1} \to +\infty,$$

as $k \to \infty$ due to $a < b$ and because λ_1, λ_2 are constants that depend only on f_1, f_2.

In both cases, we conclude that, unless $f_1 = f_2$, $\Delta(k) > 0$ for sufficiently large $k \geq K$ where K is finite. This establishes the lemma for continuous distributions.

In the discrete or categorical case, the argument remains the same, only adapted to looking at the finite set of values on which $f_1 \geq f_2$. The largest value less than a above which equality holds until the end of the support then determines the growth of the difference sequence in the same way as was argued in Section 4.1. □

S1.2 Proof of Theorem 2

Let f_1, f_2 be the density functions of F_1, F_2. Call $\Omega = \text{supp}(F_1) \cup \text{supp}(F_2) = [0, a]$ the common support of both densities, and take $\xi = \inf\{x \in \Omega : f_1(x) = f_2(x) = 0\}$. Suppose there were an $\varepsilon > 0$ so that $f_1 > f_2$ on every interval $[\xi - \delta, \xi]$ whenever $\delta < \varepsilon$, i.e., f_1 would be larger than f_2 until both densities vanish (notice that $f_1 = f_2 = 0$ on the right of ξ). Then the proof of lemma 2 delivers the argument by which we would find a $K \in \mathbb{N}$ so that $\mathrm{E}(X_1^k) > \mathrm{E}(X_2^k)$ for every $k \geq K$, which would contradict $F_1 \preceq F_2$. Therefore, there must be a neighborhood $[\xi - \delta, \xi]$ on which $f_1(x) \leq f_2(x)$ for all $x \in [\xi - \delta, \xi]$. The claim follows immediately by setting $x_0 = \xi - \delta$, since taking $x \geq x_0$, we end up with $\int_x^\xi f_1(t)dt \leq \int_x^\xi f_2(t)dt$, and for $i = 1, 2$ we have $\int_x^\xi f_i(t)dt = \int_x^a f_i(t)dt = \Pr\{X_i > x\}$. □

S1.3 Proof of Lemma 3

Throughout the proof, let $i \in \{1, 2\}$. The truncated distribution density that approximates f_i is $f_i(x)/(F_i(a_n) - F_i(0))$, where $[0, a_n]$ is the common support of n-th approximation to f_1, f_2. By construction, $a_{n,i} \to \infty$ as $n \to \infty$, and therefore $F_i(a_n) - F_i(0) \to 1$ for $i = 1, 2$. Consequently,

$$Q_n = \frac{F_1(a_n) - F_1(0)}{F_2(a_n) - F_2(0)} \to 1, \quad \text{as } n \to \infty,$$

and there is an index N such that $Q_n > c$ for all $n \geq N$. In turn,

$$f_2(x) \cdot Q_n > f_2(x) \cdot c > f_1(x),$$

and by rearranging terms,

$$\frac{f_1(x)}{F_1(a_n) - F_1(0)} < \frac{f_2(x)}{F_2(a_n) - F_2(0)}, \tag{2}$$

for all $x \geq x_0$ and all $n \geq N$. The last inequality (2) lets us compare the two approximations easily by the same arguments as have been used in the proof of lemma 2, and the claim follows.
