# Supplementary material for: Decisions with Uncertain Consequences—A Total Ordering on Loss-Distributions
Source: PLoS One. 2016 Dec 28;11(12):e0168583. doi: 10.1371/journal.pone.0168583 (PMC5193423; doi:10.1371/journal.pone.0168583)
Supplement: S1 File — Both are available as supporting information to this article. (ZIP) [file pone.0168583.s002.zip › 1506.07368v4.pdf]

arXiv:1506.07368v4 [q-fin.EC] 17 Sep 2015

On Game-Theoretic Risk Management (Part One)

Towards a Theory of Games with Payoffs that are Probability-Distributions

Stefan Rass *
stefan.rass@aau.at

September 18, 2015

Abstract

Optimal behavior in (competitive) situation is traditionally determined with the help of utility functions that measure the payoff of different actions. Given an ordering on the space of revenues (payoffs), the classical axiomatic approach of von Neumann and Morgenstern establishes the existence of suitable utility functions, and yields to game-theory as the most prominent materialization of a theory to determine optimal behavior. Although this appears to be a most natural approach to risk management too, applications in critical infrastructures often violate the implicit assumption of actions leading to deterministic consequences. In that sense, the gameplay in a critical infrastructure risk control competition is intrinsically random in the sense of actions having uncertain consequences. Mathematically, this takes us to utility functions that are probability-distribution-valued, in which case we loose the canonic (in fact every possible) ordering on the space of payoffs, and the original techniques of von Neumann and Morgenstern no longer apply.

This work introduces a new kind of game in which uncertainty applies to the payoff functions rather than the player's actions (a setting that has been widely studied in the literature, yielding to celebrated notions like the trembling hands equilibrium or the purification theorem). In detail, we show how to fix the non-existence of a (canonic) ordering on the space of probability distributions by only mildly restricting the full set to a subset that can be totally ordered. Our vehicle to define the ordering and establish basic game-theory is non-standard analysis and hyperreal numbers.

*Universität Klagenfurt, Institute of Applied Informatics, System Security Group, Universitätsstrasse 65-67, 9020 Klagenfurt, Austria. This work has been done in the course of consultancy for the EU Project HyRiM (Hybrid Risk Management for Utility Networks; see https://hyrim.net), led by the *Austrian Institute of Technology* (AIT; www.ait.ac.at). See the acknowledgement section.

Contents

1 Introduction 3
1.1 Symbols and Notation . . . 5

2 Optimal Decisions under Uncertainty 10
2.1 Choosing Actions (In)dependently . . . 12
2.2 Comparing Payoff Distributions . . . 13
2.3 Comparing Discrete and Continuous Distributions . . . 17
2.4 Comparing Deterministic to Random . . . 18
2.5 Extensions: Relaxing Assumption 1.3 . . . 18
2.6 Interpretation and Implications of Preferences . . . 22

3 Games with Uncertain Payoffs 26
3.1 Arithmetic in $\mathfrak{F} \subset \mathbb{R}^\infty/\mathcal{U}$ . . . 26
3.2 Continuity of $F(\boldsymbol{p}, \boldsymbol{q})(r)$ in $(\boldsymbol{p}, \boldsymbol{q})$ . . . 27
3.3 Security Strategies and Zero-Sum Games . . . 28

4 Optimizing Multiple Security Goals 30
4.1 Characterization and Existence of Security Strategies . . . 30
4.2 Relation to Bayesian Decisions . . . 33

5 Compiling Quantitative Risk Measures 33

6 Outlook 35

1 Introduction

Security risk management is a continuous cycle of action and reaction to the changing working conditions of an infrastructure. This cycle is detailed in relevant standards like ISO 2700x, where phases designated to *planning*, *doing*, *checking* and *acting* are rigorously defined and respective measures are given.

Our concern in this report is an investigation of a hidden assumption underneath this recommendation, namely the hypothesis that some wanted impact can be achieved by taking the proper action. If so, then security risk management would degenerate to a highly complex but nevertheless deterministic control problem, to which optimal solutions and strategies could be found (at least in theory).

Unfortunately, however, reality is intrinsically random to some extent, and the outcome of an action is almost never fully certain. Illustrative examples relate to how public opinion and trust are dependent on the public relation strategies of an institution. While there are surely ways to influence the public opinion, it will always be ultimately out of one's full and exclusive control. Regardless of this, we ought to find optimal ways to influence the situation in the way we like. This can – in theory – again be boiled down to a (not so simple) optimization problem, however, one that works on optimizing partially random outcomes. This is where things start to get nontrivial.

Difficulties in the defense against threats root in the nature of relevant attacks, since not all of them are immediately observable or induce instantly noticeable or measurable consequences. Indeed, the best we can do is finding an optimal protection against an a-priori identified set of attack scenarios, so as to gain the assurance of security against the known list of threat scenarios. Optimizing this protection is often, but not necessarily, tied to some kind of *adversary modelling*, in an attempt to sharpen our expectations about what may happen to us. Such adversary modeling is inevitably error prone, as the motives and incentives of an attacker may deviate from our imagination to an arbitrary extent.

Approaching the problem mathematically, there are two major lines of decision making: one works with an a-priori hypothesis of the current situation, and incorporates current information into an a-posteriori model that tells us how things will evolve, and specifically, which events are more likely than others, given the full information that we have. Decision making in that case means that we seek the optimal behavior so as to master a *specifically* expected setting (described by the a-posteriori distribution). This is the *Bayesian* approach to decision making (see [10] for a fully comprehensive detailed). The second way of decision making explicitly avoids any hypothesis about the current situation, and seeks an optimal behavior against *any* possible setting. Unlike the Bayesian perspective, we would thus intentionally and completely ignore all available data and choose our actions to master the worst-case scenario. While this so-called *minimax decision making* is obviously a more pessimistic and cautious approach, it appears better suited for risk management in situations where data is either not available, not trustworthy or inaccurate.

For this reason, we will hereafter pursue the minimax-approach and dedicate section 4.2 to a discussion how this fits into the Bayesian framework as a special case.

We assume that the risk manager can repeatedly take actions and that the possible actions are finitely many. Furthermore, we assume that the adversary against which we do our risk control, also has a finite number of possible ways to cause trouble. In terms of an ISO 2700k risk management process, the risk manager's actions would instantiate *controls*, while the adversary's actions would correspond to identified threat scenarios. The assumption of finiteness does stringently constrain us here, as an infinite number of actions to choose from may in any case overstrain a human decision-maker.

The crucial point in all that follows is that any action (as taken by the decision maker) in any situation (action taken by the adversary) may have an intended but in any case random outcome. To properly formalize this and fit it into a mathematical, in fact game-theoretic, framework, we hereafter associate the risk manager with *player 1* in our game, who competes with *player 2*, who is the adversary. Actions of either players are in game-theoretic literature referred to as *pure strategies*; the entirety of which will be abbreviated as PS_1 and PS_2 for either player, so PS_1 comprises all actions, hereafter called *strategies* available for risk management, while PS_2 comprises all trouble scenarios. For our treatment, it is not required to be specific on how the elements in both action sets look like, as it is sufficient for them to "be available".

Let PS_1, PS_2 denote finite sets of strategies for two players, where player 1 is the honest defender (e.g., utility infrastructure provider), and player 2 is the adversary. We assume player 1 to be unaware of its opponents incentives, so that an optimal strategy is sought against any possible behavior within the *known* action space PS_2 of the opponent (rational or irrational, e.g., nature),.

In this sense, PS_2 can be the set of all known possible security incidents, whose particular incarnations can become reality by the adversary's action. To guard its assets, player 1 can choose from a finite set of actions PS_1 to minimize the costs of a recovery from any incident, or equivalently, keep its risk under control.

Upon these assumptions, the situation can be described by an $(n \times m)$-matrix of scenarios, where $n = |PS_1|, m = |PS_2|$, each of which is associated with some cost R_{ij} to recover the system from a malfunctioning state back to normal operation from scenario $(i, j) \in PS_1 \times PS_2$. We use the variable R_{ij} henceforth to denote the cost of a *repair* made necessary by an incident $j \in PS_2$ happening when the system is currently in configuration $i \in PS_1$.

The process of risk management will be associated with player 1 putting the system into different configurations over time in order to minimize the risk R_{ij}.

Remark 1.1 *We leave the exact understanding of "risk" or "damage" intentionally undefined here, as this will be quite different between various utility infrastructures or general fields of application.*

Remark 1.2 *Neither the set PS_1 nor the set PS_2 is here specified in any detail further than declaring it as an "action space". The reason is, again, the expected diversity of actions and incidents among various fields of application (or utility infrastructures). Therefore and to keep this report as general and not limiting the applicability of the results to follow, we will leave the precise elements of PS_1, PS_2 up to definitions that are tailored to the intended application.*

Examples of strategies may include:

- *random spot checks in the system to locate and fix problems (ultimately, to keep the system running),*
- *random surveillance checks and certain locations,*
- *certain efforts or decisions about whether or not, and which, risks or countermeasures shall be communicated to the society or user community,*
- *etc.*

In real life settings, it can be expected that an action (regardless of who takes it), always has some intrinsic randomness. That is, the effect of a particular scenario $(i, j) \in PS_1 \times PS_2$ is actually a random variable R_{ij}, having only some "expected" outcome that may be different between any two occurrences of the same situation (i, j) over time.

To be able to properly handle the arising random variables, let us think of those modeling not the benefits but rather the *damage* that a security incident may cause. In this view, we can go for minimization of an expectedly positive value that measures the cost of a recovery. Formally, we introduce the following assumption that will greatly ease theoretical technicalities throughout this work, while not limiting the practicability too much.

The family $\{R_{ij} : i \in PS_1, j \in PS_2\}$ of random damage distributions in our game will be assumed with all members satisfying the following assumption:

Assumption 1.3 *Let R_{ij} be a real-valued random variable. On R_{ij}, we impose the following assumptions:*

- $R_{ij} \geq 1$ *(w.l.o.g.[1]).*
- *R_{ij} has a known distribution F_{ij} with compact support (note that this implies that all R_{ij} is upper-bounded).*
- *The probability measure induced by F_{ij} is either discrete or continuous and has a density function f_{ij}. For continuous random variables, the density function is assumed to be continuous.*

1.1 Symbols and Notation

This section is mostly intended to refresh the reader's memory about some basic but necessary concepts from calculus and probability theory that we will use in the following to develop the theoretical groundwork. This subsection can thus be safely skipped and may be consulted whenever necessary to clarify details.

[1]It is common to assume losses to be ≥ 0; our modification has technical reasons, but causes no semantic difference in the comparisons between two loss densities, since both loss variables are just shifted by the same amount. Also, the loss can (w.l.o.g.) be scaled until losses in the range $[0, 1)$ become practically negligible.

General Symbols: Sets, random variables and probability distribution functions are denoted as upper-case letters like X or F. Matrices and vectors are denoted as bold-face upper- and lower-case letters, respectively. For finite sets, we write $|X|$ for the number of elements (cardinality). For real values $|a|$ denotes the absolute value of $a \in \mathbb{R}$. For arbitrary sets, the symbol X^k is the k-fold cartesian product of X; the set X^∞ thus represents the collection of all infinite sequences $(a_1, a_2, a_3, \ldots)$ with elements from X. We denote such a sequence as $(a_n)_{n \in \mathbb{N}}$.

If X is a random variable, then its probability distribution F_X is told by the notation $X \sim F_X$. Whenever this is clear from the context, we omit the subscript to F and write $X \sim F$ only. If X lives on a discrete set, then we call X a *discrete random variable*. Otherwise, if X takes values in some infinite and uncountable set, such as $\mathbb{R}$, then we call X a *continuous random variable*. For discrete distributions, we may also use the vector $\boldsymbol{p}$ of probabilities of each event to denote the distribution of the discrete variable X as $X \sim \boldsymbol{p}$.

Calligraphic letters denote families (sets) of sets or probability distributions, e.g., ultrafilters (defined below) are denoted as $\mathcal{U}$, or the family of all probability distributions being denoted as $\mathfrak{F}$. The family of subsets of a set A is written as $\mathcal{P}(A)$ (the *power-set* of A). If $F \in \mathfrak{F}$ is a probability distribution, then its density – provided it exists – is denoted by the respective lower-case letter f.

Topology and Norms: As our considerations in section 3 will heavily rely on concepts of continuity and compactness or openness of sets, we briefly review the necessary concepts now.

A set A is called *open*, if for every $x \in A$ there is another open set $B \subset A$ that contains x. The family $\mathcal{T}$ of all open sets is characterized by the property of being closed under infinite union and finite intersection. Such a set is called a *topology*, and the set X together with a topology $\mathcal{T} \subset \mathcal{P}(X)$ is called a *topological space*. An interval A is called *closed*, if its complement (w.r.t. the space X) is open.

In $\mathbb{R}$, it can be shown that the open intervals are all of the form $\{x : a < x < b\}$ for $a, b \in \mathbb{R}$ and $a < b$. We denote these intervals by (a, b) and the topology on $\mathbb{R}$ is the set containing all of them. Note the existence of a total ordering $\leq$ on a space always induces the so-called *order-topology*, whose open sets are defined exactly the aforementioned way. Closed intervals are denoted by square brackets, $[a, b] = \{x : a \leq x \leq b\}$. An set $X \subset \mathbb{R}$ is called *bounded*, if there are two constants $a, b < \infty$ so that all $x \in X$ satisfy $a < x < b$. An subset of $\mathbb{R}$ is called *compact*, if and only if it is closed and bounded.

For $(X, d_X), (Y, d_Y)$ being two metric spaces, we call a function $f : X \to Y$ *continuous*, if for every $x_0 \in X$ and every $\varepsilon > 0$ there is some $\delta > 0$ for which $d_X(x_0, y) < \delta$ implies $d_Y(f(x_0), f(y)) < \varepsilon$. If the condition holds with the same ε, δ for every $x_0 \in A \subseteq X$, then we call f *uniformly continuous* on the set A. It can be shown that if a function f is continuous on a compact set A, then it is also uniformly continuous on A (in general, however, continuity does not imply uniform continuity). In the following, we will need this result only on functions mapping compact subsets of $\mathbb{R}$ to probability distributions (the space that we consider there will be the set of hyperreal numbers, which has a

topology but – unfortunately – neither a metric nor a norm).

On a space X, we write $\|\boldsymbol{x}\|$ to denote the norm of a vector $\boldsymbol{x}$. One example is the ∞-norm on $\mathbb{R}^n$, which is $\|\boldsymbol{x}\|_\infty = \|(x_1, \ldots, x_n)\|_\infty = \max\{|x_1|, \ldots, |x_n|\}$ for every $\boldsymbol{x} \in \mathbb{R}^n$. This induces the metric $d_\infty(\boldsymbol{x}, \boldsymbol{y}) = \|\boldsymbol{x} - \boldsymbol{y}\|_\infty$.

It can be shown that every metric space is also a topological space, but the converse is not true in general. However, the above definition of continuity is (on metric spaces) equivalent to saying that a function $f : X \to Y$ is continuous, if and only if every open set in $Y \in \mathcal{T}_\mathcal{Y}$ has an open preimage $f^{-1}(B) \in \mathcal{T}_\mathcal{X}$, when $\mathcal{T}_X, \mathcal{T}_Y$ denote the topologies on X and Y, respectively. This characterization works without metrics and will be used later to prove continuity of payoff functions (see lemma 3.1 and proposition 3.2).

Probabilities and Moments: Let $A \subset \Omega$ be a subset of some measurable[2] space Ω and F be a probability distribution function. The *probability measure* $\Pr_F(A)$ is the Lebesgue-Stieltjes integral $\Pr_F(A) = \int_A dF$ (note that this general formulation covers both, discrete and continuous random variables on the same formal ground). Whenever the distribution is obvious from the context, we will omit the subscript to the probability measure, and simply write $\Pr(A)$ as a shorthand of $\Pr_F(A)$.

All probability distribution functions F that we consider in this report will have a density function f associated with them. If so, then we call the closure of the set $\{x : f(x) > 0\}$ the *support* of F, denoted as $\text{supp}(F)$. A *degenerate distribution* on $\mathbb{R}$ is one that assigns probability mass 1 to a finite number (or more generally, a null-set) of points in $\mathbb{R}$. If $\Pr(A) = 1$ for a singleton set $A = \{a\}$ and $a \in \mathbb{R}$, then we call this degenerate distribution a *point-mass* or a *Dirac-mass*. We stress that such distributions do not have a density function associated with them in general[3].

Many commonly used distributions have infinite support, such as the Gaussian distribution. The density function can, however, be cut off outside a bounded range $[a, b]$ and re-scaled to normalize to a probability distribution again. This technique lets us approximate any probability distribution by one with compact support (a technique that will come handy in section 2.5).

The *expectation* of a random variable is (by the law of large numbers) the long-run average of realizations, or more rigorously, defined as $\mathrm{E}(X) = \int_\Omega x dF(x) dx$. The k-th moment of a distribution is the expectation of X^k, which we is denoted and defined as $m_X(k) := \mathrm{E}(X^k) = \int_\Omega x^k dF(x)$, or also $\mathrm{E}(X^k) = \int_\Omega x^k f(x) dx$, if F has a density function f. Special roles play the first four moments or values derived thereof. One prominent example is the *variance* $\mathrm{Var}(X) = \mathrm{E}(X - \mathrm{E}(X))^2 = \mathrm{E}(X^2) - (\mathrm{E}(X)^2)$ (this formula is known as Steiner's theorem). Of particular importance is the so-called *moment-generating function* $\mu_X(s) = \mathrm{E}(\exp(s \cdot X))$, from which the k-th moment can be computed by taking the k-th order derivative evaluated at the origin, i.e., we have $\mathrm{E}(X^k) = \frac{d^k}{ds^k} \mu_X(s)\Big|_{s=0}$. Moments do not necessarily exist for all distributions (an

[2]We will not require any further details on measurability or σ-algebras in this report, so we spare details or an intuitive explanation of the necessary concepts here.

[3]at least not within the space of normal functions; the Dirac-mass is, however, an irregular generalized function (a concept that we will not need here).

example is the Cauchy-distribution, for which all moments are infinite), but exist for all distributions with compact support (that can be used to approximate every other distribution up to arbitrary precision).

Multivariate distributions model vector-valued random variables. Their distribution is denoted as $F_{X,Y}$, or shorthanded as F. For an n-dimensional distribution, the respective density function is then of the form $f(x_1, \ldots, x_n)$, having the integral $\int_{\mathbb{R}^n} f(x_1, \ldots, x_n) d(x_1, \ldots, x_n) = 1$. This joint distribution in particular models the interplay between the (perhaps mutually dependent) random variables $X_1, \ldots, X_n$. The *marginal distribution* of any of the variables X_i (where $1 \leq i \leq n$) is the unconditional distribution of X_i no matter what the other variables do. Its density function is obtained by "integrating out" the other variables, i.e.,

$$f_{X_i}(x_i) = \int_{\mathbb{R}^{n-1}} f(x_1, \ldots, x_{i-1}, x_{i+1}, \ldots, x_n) d(x_1, \ldots, x_{i-1}, x_{i+1}, \ldots, x_n).$$

A (marginal) distribution is called *uniform*, if its support is bounded and its density is a constant. The joint probability of a multivariate event, i.e., multidimensional set A w.r.t. to a multivariate distribution $F_{X,Y}$, is denoted as $\Pr(F_{X,Y})(A)$. That is, the distribution w.r.t. which the probabilities are taken are given in the subscript, whenever this is useful or necessary to make things clear.

A particular important class of distributions are *copulas*. These are multivariate probability distribution functions on the n-dimensional hypercube $[0,1]^n$, for which all marginal distributions are uniform. The importance of copula functions is due to Sklårs theorem, which tells that the joint distribution F of the random vector $(X_1, \ldots, X_n)$ can be expressed in terms of marginal distribution functions and a copula function C as $F(x_1, \ldots, x_n) = C(F_1(x_1), \ldots, F_n(x_n))$. So, for example, independence of events can be modeled by the simple product copula $C(x_1, \ldots, x_n) = x_1 \cdot x_2 \cdots x_n$. Many other classes of copula functions and a comprehensive discussion of the topic as such can be found in [8].

Convexity and Concavity: Let V be a vector-space. We call a set $A \subset V$ *convex*, if for any two points $x, y \in A$, the entire line connecting x to y is also contained in A. Let $f : \mathbb{R} \to \mathbb{R}$ be a function and take two values $a < b$. The function f is called *convex*, if for every two values x, y, the line between $f(a)$ and $f(b)$ upper-bounds f between a and b. More formally, let $L_{a,b}(x)$ be the straight line from $f(a)$ to $f(b)$, then f is convex if $f(x) \leq L_{a,b}(x)$ for all $a \leq x \leq b$. A function f is called *concave* if $(-f)$ is convex.

Hyperreal Numbers and Ultrafilters: Take the set $\mathbb{R}^\infty$ of infinite sequences over the real numbers $\mathbb{R}$. On this set, we can define the arithmetic operations $+$ and $\cdot$ elementwise on two sequences $a = (a_1, a_2, a_3, \ldots) = (a_n)_{n \in \mathbb{N}} \in \mathbb{R}^\infty$ and $b = (b_1, b_2, b_3, \ldots) = (b_n)_{n \in \mathbb{N}} \in \mathbb{R}^\infty$ by setting $a + b = (a_1 + b_1, a_2 + b_2, a_3 + b_3, \ldots)$ and $a \cdot b = (a \cdot b_1, a \cdot b_2, a_3 \cdot b_3, \ldots)$. The ordering of the reals, however, cannot be carried over in this way, as the sequences $a = (1, 4, 2, \ldots)$ and $b = (2, 1, 4, \ldots)$ would satisfy $\leq$ on some components and $\geq$ on some others. To fix this, we need to be specific on which indices matter for the

comparison, and which do not. The resulting family of index-sets can be characterized to be a so-called *free ultrafilter*, which is defined as follows: a family $\mathcal{U} \subseteq \mathcal{P}(\mathbb{N})$ is called a *filter*, if the following three properties are satisfied:

- $\emptyset \notin \mathcal{U}$
- closed under supersets: $A \subseteq B$ and $A \in \mathcal{U}$ implies $B \in \mathcal{U}$
- closed under intersection: $A, B \in \mathcal{U}$ implies $A \cap B \in \mathcal{U}$

If, in addition, $A \notin \mathcal{U}$ implies that $\mathcal{U}$ contains the complement set of A, then $\mathcal{U}$ is called an *ultrafilter.* A simple example of a filter is the *Fréchet*-filter, which is the family $\{A : \text{the complement of } A \text{ is finite}\}$. A filter is called *free*, if it contains no finite sets, or equivalently, if any filter that contains $\mathcal{U}$ is equal to $\mathcal{U}$, i.e., $\mathcal{U}$ is maximal w.r.t. the $\supseteq$-relation. An application of Zorn's lemma to the semi-ordering induced by $\supseteq$ shows the existence of free ultrafilter as being $\supseteq$-maximal elements, extending the Fréchet-filter.

An ultrafilter naturally induces an equivalence relation on $\mathbb{R}^\infty$ by virtue of calling two sequences $a = (a_n)_{n\in\mathbb{N}}, b = (b_n)_{n\in\mathbb{N}}$ $\equiv_{\mathcal{U}}$-equivalent, if and only if $\{i : a_i = b_i\} \in \mathcal{U}$, i.e., the set of indices on which a and b coincide belongs to $\mathcal{U}$. The $\leq$- and $\geq$-relations can be defined in exactly the same fashion. The family of equivalence classes modulo $\mathcal{U}$ makes up the set of *hyperreal numbers*, i.e., ${}^*\mathbb{R} = \{[a]_{\mathcal{U}} : a \in \mathbb{R}^\infty\} = \mathbb{R}^\infty/\mathcal{U}$, where $[a]_{\mathcal{U}} = \{b \in \mathbb{R}^\infty : a \equiv_{\mathcal{U}} b\}$. In lack of an exact model of ${}^*\mathbb{R}$ due to the non-constructive existence assurance of the necessary free ultrafilter, unfortunately, we are unable to practically do arbitrary arithmetic in ${}^*\mathbb{R}$. It will be shown (later and in part two of this report) that everything that needs to be computed practically works without $\mathcal{U}$ being explicitly known.

Elements of Game Theory: Let $N = \{1, 2, \ldots, n\}$ be a finite set. Let PS_i be a finite set of actions, and denote by PS_{-i} the cartesian product $PS_{-i} = PS_1 \times PS_2 \times \cdots \times PS_{i-1} \times PS_{i+1} \times \cdots \times PS_n$, i.e., the product of all PS_j *excluding* PS_i.

A finite non-cooperative n-person game is a triple (N, H, S), where the set $H = \{u_i : PS_i \times PS_{-i} \to \mathbb{R} : i \in N\}$ contains all player's payoff functions, and the family $S = \{PS_1, \ldots, PS_n\}$ comprises the strategy sets of all players. The attribute *finite* is given to the game if and only if all PS_i are finite. An *equilibrium strategy* is an element $x^* = (x_1^*, \ldots, x_n^*) \in \prod_{i=1}^n PS_i$, so that all $i \in N$ have

$$u_i(x_i^*, x_{-i}^*) \geq u_i(x_i, x_{-i}^*). \qquad (1)$$

That is, action x_i^* gives the maximal outcome for the i-th player, provided that all other players follow their individual equilibrium strategies. Otherwise said, no player has an incentive to solely deviate from x_i^*, as this would only worsen the revenue from the gameplay[4]. It is easy to construct examples in which no such equilibrium strategy

[4]It should be mentioned that this not necessarily rules out benefits for *coalitions* of players upon jointly deviating from the equilibrium strategy. This, however, is subject of cooperative game-theory, which we do not discuss here any further.

exists. To fix this, one usually considers *repetitions* of the gameplay, and defines the revenue for a player as the *long-run average* of all payoffs in each round. Technically, this assures the existence of equilibrium strategies in all finite games (Nash's theorem). We will implicitly rely on this possibility here too, while explicitly looking at the outcome of the game in a single round. As this is – by our fundamental hypotheses in this report – a random variable itself, condition (1) can no longer be soundly defined, as random variables are not canonically ordered. The core of this work will therefore be on finding a substitute for the $\geq$-relation, so as to properly restate (1) when random variables appear on both sides of the inequality.

2 Optimal Decisions under Uncertainty

Under the above setting, we can collect all scenarios of actions that player 1 (defender) and player 2 (attacker) may take in a tabular (matrix) fashion. Our goal in this first step, is to soundly define what "a best action" would be in light of uncertain, indeed random, effects that actions on either side cause, especially in lack of control about the other's actions. For that matter, we will consider the scenario matrix $\mathbf{A}$ as given below, as the payoff structure of some matrix-game, whose mathematical underpinning is the standard setup of game-theory (see [3] for example), with differences and necessary changes to classical theory of games, being discussed in sections 3 and later.

Let the following tableau be a collection of all scenarios of actions taken by the defender (row-player) and attacker (column-player),

$$\mathbf{A} = \begin{pmatrix} R_{11} & \cdots & R_{1j} & \cdots & R_{1m} \\ \vdots & \ddots & \vdots & \ddots & \vdots \\ R_{i1} & \cdots & R_{ij} & \cdots & R_{im} \\ \vdots & \ddots & \vdots & \ddots & \vdots \\ R_{n1} & \cdots & R_{nj} & \cdots & R_{nm} \end{pmatrix},$$

where the rows of the matrix $\mathbf{A}$ are labeled by the actions in PS_1, and the columns of $\mathbf{A}$ carry the labels of actions from PS_2.

A *security strategy* for player 1 is an optimal choice i^* of a row so that the risk, expressed by the random variable R_{i^*j} is "optimized" over all possible actions $j \in PS_2$ of the opponent. Here, we run into trouble already, as there is no canonical ordering on the set of probability distributions.

To the end of resolving this issue, let us consider repetitions of the gameplay in which each player can choose his actions repeatedly and differently, in an attempt to minimize risk (or damage). This corresponds to situations in which "the best" configuration simply does not exist, and we are forced to repeatedly change or reconsider the configuration of the system in order to remain protected.

In a classical game-theoretic approach, this takes us to the concept of *mixed strategies*, which are discrete probability distributions over the action spaces of the players. Making this rigorous, let $S(PS_i)$ for $i = 1, 2$ denote the simplex over PS_i, i.e., the space of all

discrete probability distributions supported on PS_i. More formally, given the support X, the set $S(X)$ is

$$S(X) := \left\{ (p_1, \ldots, p_k) \in \mathbb{R}^k : k = |X|, \sum_{i=1}^{k} p_i = 1, p_i \geq 0 \,\forall i \right\}.$$

A randomized decision is thus a rule $\boldsymbol{p} = (p_1, \ldots, p_k) \in S(PS)$ to choose from the available actions $\{1, 2, \ldots, k\}$ from the action set PS (which is PS_1 or PS_2 hereafter) with corresponding probabilities p_i. We assume the ordering of the actions to be arbitrary but fixed (for obvious reasons).

Now, we return to the problem of what effect to expect when the current configuration of the system is randomly drawn from PS_1, and the adversary's action is another random choice from PS_2. For that matter, let us simplify notation by putting $S_1 := S(PS_1), S_2 := S(PS_2)$ and let the two mixed strategies be $\boldsymbol{p} \in S_1$ for player 1, and $\boldsymbol{q} \in S_2$ for player 2.

Since the choice from the matrix $\mathbf{A}$ is random, where the row is drawn with likelihoods as specified by $\boldsymbol{p}$, and the column is drawn from $\boldsymbol{q}$, the law of total probability yields for the outcome R,

$$\Pr(R \leq r) = \sum_{i,j} \Pr(R_{ij} \leq r | i, j) \Pr(i, j), \tag{2}$$

where $\Pr(R_{ij} \leq r|i, j)$ is the conditional probability of R_{ij} given a particular choice (i, j), and $\Pr(i, j)$ is the (unconditional) probability for this choice to occur. Section 2.1 gives some more details on how $\Pr(i, j)$ can be modeled and expressed.

Denote by $F(\boldsymbol{p}, \boldsymbol{q})$ the distribution of the game's outcome under strategies $(\boldsymbol{p}, \boldsymbol{q}) \in S_1 \times S_2$, then $\Pr(R \leq r) = F(r)$ depends on $(\boldsymbol{p}, \boldsymbol{q})$, and (2) can be rewritten as

$$\Pr(R \leq r) = (F(\boldsymbol{p}, \boldsymbol{q}))(r) = \sum_{i,j} F_{ij}(r) C_{\boldsymbol{p},\boldsymbol{q}}(i, j), \tag{3}$$

where $C_{\boldsymbol{p},\boldsymbol{q}}(i, j) = \Pr(i, j)$ will be assumed as continuous in $\boldsymbol{p}$ and $\boldsymbol{q}$ for technical reasons that will become evident later (during the proof of proposition 3.2). Note that the distribution F via the function C explicitly depends on the choices $\boldsymbol{p}, \boldsymbol{q}$, and is to be "optimally shaped" w.r.t. to these two variables. The argument $r \in \mathbb{R}$ to the function $F(\boldsymbol{p}, \boldsymbol{q})(\cdot)$ is the (random) "revenue", whose uncertainty is outside any of the two player's influence (besides shaping F by proper choices of $\boldsymbol{p}$ and $\boldsymbol{q}$).

The "revenue" R in the game can be of manifold nature, such as

- Risk response of society; a quantitative measure that could rate people's opinions and confidence in the utility infrastructure
- Repair cost to recover from an incident's implied damage,
- Reliability, if the game is about whether or not a particular quality of service can be kept up,
- etc.

Remark 2.1 *In the simplest case of independent actions, we would set* $C_{\boldsymbol{p},\boldsymbol{q}}(i,j) = p_i \cdot q_j$ *when* $\boldsymbol{p} = (p_1, \ldots, p_n), \boldsymbol{q} = (q_1, \ldots, q_m)$. *This choice, along with assuming* R_{ij} *to be constants rather than random variables, recreates the familiar matrix-game payoff functional* $\boldsymbol{p}^T \mathbf{A} \boldsymbol{q}$ *from* (3). *Hence,* (3) *is a first generalization of matrix games to games with uncertain outcome, which for the sake of flexibility and generality, is "distribution-valued".*

Remark 2.2 *It may be in reality the case that actions of the two players are* not *chosen independently, for example, if both of the players possess some common knowledge or access to a common source of information. In game-theoretic terms, this would lead to so-called* correlated equilibria *(see [3]), in which the players share two correlated random variables that influence their choices. Things here are nevertheless different, as no bidirectional flow of information can be assumed like for correlated equilibria (the attacker won't inform the utility infrastructure provider about anything in advance, while information from the provider may somehow leak out to the adversary).*

2.1 Choosing Actions (In)dependently

The concrete choice of the function $C_{\boldsymbol{p},\boldsymbol{q}}$ is only subject to continuity in $\boldsymbol{p}, \boldsymbol{q}$ for technical reasons that will receive a closer look now. The general joint probability of the scenario (i, j) w.r.t. the marginal discrete distribution vectors $\boldsymbol{p}, \boldsymbol{q}$ is $\Pr_{\boldsymbol{p},\boldsymbol{q}}\{i, j\} = \Pr_{\boldsymbol{p},\boldsymbol{q}}\{X = i, Y = j\} = C_{\boldsymbol{p},\boldsymbol{q}}(i, j)$ in (2). Under independence of the random choices $X \sim \boldsymbol{p}, Y \sim \boldsymbol{q}$ can be written as $\Pr(i, j) = \Pr(X = i)\Pr(Y = j) = p_i q_j$.

Now, let us consider cases where the choices are *not* independent, say, if one player observes the other player's actions and can react on them (or if both players have access to common source of information).

Sklar's theorem implies the existence of a copula-function C so that the joint distribution $F_{(X,Y)}$ can be written in terms of the copula C and the marginal distributions F_X, corresponding to the vector $\boldsymbol{p}$, and F_Y, corresponding to the vector $\boldsymbol{q}$,

$$F_{(X,Y)}(i, j) = \Pr(X \leq i, Y \leq j) = C(F_X(i), F_Y(j)).$$

$$\begin{aligned}
\Pr(i, j) = \Pr(X = i, Y = i) &= \Pr(X \leq i, Y \leq j) - \Pr(X \leq i-1, Y \leq j) \\
&\quad - \Pr(X \leq i, Y \leq j-1) + \Pr(X \leq i-1, Y \leq j-1) \\
&= C(F_X(i), F_Y(j)) - C(F_X(i-1), F_Y(j)) \\
&\quad - C(F_X(i), F_Y(j-1)) + C(F_X(i-1), F_Y(j-1)) \\
&= C(p_i, q_j) - C(p_{i-1}, q_j) - C(p_i, q_{j-1}) + C(p_{i-1}, q_{j-1}). \qquad (4)
\end{aligned}$$

Thus, the function $C_{\boldsymbol{p},\boldsymbol{q}}$ can be constructed from (4) based on the copula C (which must exist). Continuity of $C_{\boldsymbol{p},\boldsymbol{q}}$ thus hinges on the continuity of the copula function. At least two situations admit a choice of C that makes $C_{\boldsymbol{p},\boldsymbol{q}}$ continuous:

- Independence of actions: $C(x, y) := x \cdot y$

- Complete lack of knowledge about the interplay between the action choices, in which case we can set $C(x, y) := \min\{x, y\}$.

  This choice is justified upon the well-known Fréchet-Hoeffding bound, which says that *every* n-dimensional copula function C satisfies

  $$C(u_1, u_2, \ldots, u_n) \leq \min\{u_1, \ldots, u_n\}.$$

  Since the min-function is itself a copula, it can be chosen if a dependency is known to exist, but with no details on the particular nature of the interplay. Observe that this corresponds to the well-known *maximum-principle of system security*, where the overall system risk is determined from the maximum risk among its components (alternatively, you may think of a chain to be as strong as its weakest element; which corresponds to the min-function among all indicators $u_1, \ldots, u_n$).

2.2 Comparing Payoff Distributions

There appears to be no canonical way to compare payoff distributions, as $F(\boldsymbol{p}, \boldsymbol{q})$ can be determined by an arbitrary number of parameters, thus introducing ambiguity in how to compare them. To see this, simply consider the set of normal distributions $\mathcal{N}(\mu, \sigma^2)$ being determined by two parameters μ and $\sigma > 0$. Since the pair (μ, σ) uniquely determines the distribution function, a comparison between two members $F_1, F_2 \in \mathcal{N}$ amounts to a criterion to compare two-dimensional vectors $(\mu, \sigma) \in \mathbb{R} \times \mathbb{R}^+ \subset \mathbb{R}^2$. It is well-known that $\mathbb{R}^2$ is not ordered (as being isomorphic to $\mathbb{C}$, on which provably no order exists; see [1] for a proof), and hence there is no natural ordering on the set of probability distributions either.

Despite this sounding like bad news, we can actually construct an alternative characterization of probability distributions on a new space, in which the distributions of interest, in our case $F(\boldsymbol{p}, \boldsymbol{q})$ will all be members of a totally ordered subset.

To this end, we will rely on a characterization of a probability distribution of the random variable $R \sim F(\boldsymbol{p}, \boldsymbol{q})$ via the sequence $(m_R(k))_{k \in \mathbb{N}}$ of its moments. The k-th such moment is from (3) and by assumption 1.3 found to be

$$\begin{aligned}[\mathrm{E}\left(R^k\right)](\boldsymbol{p}, \boldsymbol{q}) &= \int_{-\infty}^{\infty} x^k dF(\boldsymbol{p}, \boldsymbol{q}) = \int_{-\infty}^{\infty} x^k \sum_{i,j} f_{ij}(x) C_{\boldsymbol{p},\boldsymbol{q}}(i,j) dx \\ &= \sum_{i,j} C_{\boldsymbol{p},\boldsymbol{q}}(i,j) \int_{-\infty}^{\infty} x^k f_{ij}(x) dx = \sum_{i,j} C_{\boldsymbol{p},\boldsymbol{q}}(i,j) \mathrm{E}\left(R_{ij}^k\right), \qquad (5)\end{aligned}$$

where the sum runs over $i = 1, 2, \ldots, n$ and $j = 1, 2, \ldots, m$, and f_{ij} is the probability density of R_{ij} for all i, j. Notice that the boundedness condition in assumption 1.3 assures existence and finiteness of all these moments. However, assumption 1.3 yields even more: since $R \sim F(\boldsymbol{p}, \boldsymbol{q})$ is a random variable within $[0, \infty)$ (nonnegativity) and has finite moments by the boundedness assumption, the distribution $F(\boldsymbol{p}, \boldsymbol{q})$ is *uniquely* determined by the sequence of moments. This is made rigorous by the following lemma:

Lemma 2.3 *Let two random variables X, Y have their moment generating functions $\mu_X(s), \mu_Y(s)$ exist within a neighborhood $U_\varepsilon(0)$. Assume that $\mathrm{E}\big(X^k\big) = \mathrm{E}\big(Y^k\big)$ for all $k \in \mathbb{N}$. Then X and Y have the same distribution.*

The proof is merely a collection of well-known facts about moment generating functions and the identity of their power-series expansions. For convenience and completeness, we nevertheless give the proof in (almost) full detail.

Proof (of lemma 2.3). Let Z be a general random variable. The finiteness of the moment-generating function μ_Z within some open set $(-s_0, s_0)$ with $s_0 > 0$ yields $\mathrm{E}\big(Z^k\big) = \mu_z^{(k)}(0)$ via the k-th order derivative of μ_Z [2, Theorem 3.4.3]. Furthermore, if the moment generating function exists within $(-s_0, s_0)$, then it has a Taylor-series expansion (cf. [5, Sec.11.6.1]).

$$\mu_Z(s) = \sum_{k=0}^{\infty} \frac{\mu_Z^{(k)}(0)}{k!} s^k, \qquad \forall s \in (-s_0, s_0). \tag{6}$$

Identity of moments between X and Y (the lemma's hypothesis) thus implies the identity of the Taylor-series expansions of μ_X and μ_Y and in turn the identity $\mu_X(s) = \mu_Y(s)$ on $(-s_0, s_0)$. This equation finally implies that X and Y have the same distribution by the uniqueness theorem of moment-generating functions [2, Theorem 3.4.6]. □

Lemma 2.3 is the permission to characterize random variables only by their moment-sequence to uniquely pin-down the probability distribution, i.e., we will hereafter write $m_R(k) := \mathrm{E}\big(R^k\big)$, and use

$$(m_R(k))_{k\in\mathbb{N}}, \quad \text{to represent the random variable} \quad R \sim F(\boldsymbol{p}, \boldsymbol{q}). \tag{7}$$

Let $\mathbb{R}^\infty$ denote the set of all sequences, on which we define a partial ordering by virtue of the above characterization as follows: let $F_1 = F(\boldsymbol{p}_1, \boldsymbol{q}_1), F_2 = F(\boldsymbol{p}_2, \boldsymbol{q}_2)$ be two distributions defined by (3). As a first try, we could define a preference relation between two distributions F_1, F_2 by comparing their moment sequences element-wise, i.e., we would prefer F_1 over F_2 if the respective moments satisfy $m_{R_1}(k) \leq m_{R_2}(k)$ for all k whenever $R_1 \sim F_1$ and $R_2 \sim F_2$.

It must be stressed that without extra conditions, this ordering is at most a partial one, since we could allow infinitely alternating values for the moments in both sequences. To make the ordering total, we have to be specific on which indices matter and which don't. The result will be a standard ultrapower construction, so let $\mathcal{U}$ denote an arbitrary ultrafilter. Fortunately, the preference ordering by comparing moments elementwise is ultimately independent of the particular ultrafilter in use. This is made precise in theorem 2.5 that is implied by a simple analysis of continuous distributions. We treat these first and discuss the discrete case later, as all of our upcoming findings remain valid under the discrete setting.

The Continuous Case:

Lemma 2.4 *For any two probability distributions F_1, F_2 and associated random variables $R_1 \sim F_1, R_2 \sim F_2$ that satisfy assumption 1.3, there is a $K \in \mathbb{N}$ so that either $[\forall k \geq K : m_{R_1}(k) \leq m_{R_2}(k)]$ or $[\forall k \geq K : m_{R_1}(k) \geq m_{R_2}(k)]$.*

Proof. Let f_1, f_2 denote the densities of the distributions F_1, F_2. Fix the smallest $b^* > 1$ so that $\Omega := [1, b^*]$ covers both the supports of F_1 and F_2. Consider the difference of the k-th moments, given by

$$\Delta(k) := \mathrm{E}\left(R_1^k\right) - \mathrm{E}\left(R_2^k\right) = \int_\Omega x^k f_1(x)dx - \int_\Omega x^k f_2(x)dx$$
$$= \int_\Omega x^k (f_1 - f_2)(x)dx. \tag{8}$$

Towards a lower bound to (8), we distinguish two cases:

1. If $f_1(x) > f_2(x)$ for all $x \in \Omega$, then $(f_1 - f_2)(x) > 0$ and because f_1, f_2 are continuous, their difference attains a minimum $\lambda_2 > 0$ on the compact set Ω. So, we can lower-bound (8) as $\Delta(k) \geq \lambda_2 \int_\Omega x^k dx \to +\infty$, as $k \to \infty$.

2. Otherwise, we look at the right end of the interval Ω, and define

$$a^* := \inf \{x \geq 1 : f_1(x) > f_2(x)\}.$$

Without loss of generality, we may assume $a^* < b^*$. To see this, note that if $f_1(b^*) \neq f_2(b^*)$, then the continuity of $f_1 - f_2$ implies $f_1(x) \neq f_2(x)$ within a range $(b^* - \varepsilon, b^*]$ for some $\varepsilon > 0$, and a^* is the supremum of all these ε. Otherwise, if $f_1(x) = f_2(x)$ on an entire interval $[b^* - \varepsilon, b^*]$ for some $\varepsilon > 0$, then $f_1 \not> f_2$ on Ω (the opposite of the previous case) implies the existence of some $\xi < b^*$ so that $f_1(x) < f_2(x)$, and a^* is the supremum of all these ξ (see figure 1 for an illustration). In case that $\xi = 0$, we would have $f_1 \geq f_2$ on Ω, which is either trivial (as $\Delta(k) = 0$ for all k if $f_1 = f_2$) or otherwise covered by the previous case.

In either situation, we can fix a compact interval $[a, b] \subset (a^*, b^*) \subset [1, b^*] = \Omega$ and two constants $\lambda_1, \lambda_2 > 0$ (which exist because f_1, f_2 are bounded as being continuous on the compact set Ω), so that the function

$$\ell(k, x) := \begin{cases} -\lambda_1 x^k, & \text{if } 1 \leq x < a; \\ \lambda_2 x^k, & \text{if } a \leq x \leq b. \end{cases}$$

lower-bounds the difference of densities in (8) (see figure 1), and

$$\Delta(k) = \int_1^{b^*} x^k (f_1 - f_2)(x)dx \geq \int_1^b \ell(x, k)dx$$
$$= -\lambda_1 \int_1^a x^k dx + \lambda_2 \int_a^b x^k dx$$
$$= -\frac{a^{k+1}}{k+1}(\lambda_1 + \lambda_2) + \lambda_2 \frac{b^{k+1}}{k+1} \to +\infty,$$

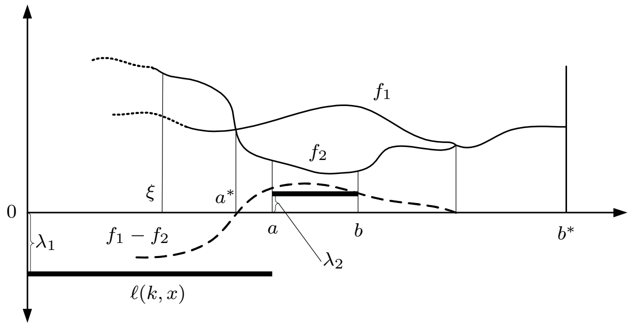

Figure 1: Lower-bounding the difference of densities

as $k \to \infty$ due to $a < b$ and because λ_1, λ_2 are constants that depend only on f_1, f_2. In both cases, we conclude that, unless $f_1 = f_2$, $\Delta(k) > 0$ for sufficiently large $k \geq K$ where K is finite.

□

Theorem 2.5 *Let $\mathfrak{F}$ be the set of distributions that satisfy assumption 1.3. Assume the elements of $\mathfrak{F}$ to be represented by hyperreal numbers in $\mathbb{R}^\infty/\mathcal{U}$, where $\mathcal{U}$ is any free ultrafilter. There exists a total ordering on the set $\mathfrak{F}$ that is independent of $\mathcal{U}$.*

Proof. Let F_1, F_2 be two probability distributions, and let $R_1 \sim F_1, R_2 \sim F_2$. Lemma 2.4 assures the existence of some $K \in \mathbb{N}$ so that, w.l.o.g, we may define the ordering $F_1 \preceq F_2$ iff $m_{R_1}(k) \leq m_{R_2}(k)$ whenever $k \geq K$. Let L be the set of indices where $m_{R_1}(k) \leq m_{R_2}(k)$, then complement set $\mathbb{N} \setminus L$ is finite (it has at most $K-1$ elements). Let $\mathcal{U}$ be an arbitrary ultrafilter. Since $\mathbb{N} \setminus L$ is finite, it cannot be contained in $\mathcal{U}$ as $\mathcal{U}$ is free. And since $\mathcal{U}$ is an ultrafilter, it must contain the complement a set, unless it contains the set itself. Hence, $L \in \mathcal{U}$, and the claim follows. The converse case is treated analogously. □

Now, we can state our preference criterion on distributions on the quotient space $\mathfrak{F} \subset \mathbb{R}^\infty/\mathcal{U}$, in which each probability distribution of interest is represented by its sequence of moments. Thanks to theorem 2.5, there is no need to construct the ultrafilter $\mathcal{U}$ in order to well-define best responses, since two distributions will compare in the same fashion under any admissible choice of $\mathcal{U}$.

Definition 2.6 (Preference Relation over Probability Distributions) *Let R_1, R_2 be two random variables whose distributions F_1, F_2 satisfy assumption 1.3. We* prefer F_1 over F_2 *relative to an ultrafilter $\mathcal{U}$, written as*

$$F_1 \preceq F_2 :\Longleftrightarrow \exists K \in \mathbb{N} \text{ s.t. } \forall k \geq K : m_{R_1}(k) \leq m_{R_2}(k) \tag{9}$$

Strict preference *of* F_1 *over* F_2 *is denoted as*

$$F_1 \prec F_2 :\iff \exists K \in \mathbb{N} \text{ s.t. } \forall k \geq K : m_{R_1}(k) < m_{R_2}(k)$$

Theorem 2.5 establishes this definition to be compatible with (in the sense of being a continuation of) the ordering on the hyperreals $\mathbb{R}^\infty/\mathcal{U}$, being defined as $a \leq b$ iff $\{i : a_i \leq b_i\} \in \mathcal{U}$, when a, b are represented by sequences $(a_i)_{i\in\mathbb{N}}, (b_i)_{i\in\mathbb{N}}$.

By virtue of the $\preceq$-relation, we can define an equivalence $\equiv$ between two distributions in the canonical way as

$$F_1 \equiv F_2 :\iff (F_1 \preceq F_2) \wedge (F_2 \preceq F_1). \tag{10}$$

Within the quotient space $\mathfrak{F} \subset \mathbb{R}^\infty/\mathcal{U}$, we thus consider two distributions as identical, if only a finite set of moments between them mismatch. Observe that this *does not* imply the identity of the distribution functions themselves, unless actually all moments match.

The strict preference relation $\prec$ induces an ordering topology $\mathcal{T}$ on $\mathfrak{F}$, whose open sets are for any two distributions F_1, F_2,

$$(F_1, F_2) := \{F \in \mathfrak{F} : F_1 \prec F \prec F_2\},$$

and the topology is denoted as $\mathcal{T} = \{(F_1, F_2) | F_1, F_2 \in \mathfrak{F} \text{ where } F_1 \prec F_2\}$.

The Discrete Case: In situations where the game's payoffs are better modeled by discrete random variables, say if a nominal scale ("low", "medium", "high") or a scoring scheme is used to express revenue, assumption 1.3 is too strong in the sense of prescribing a continuous density where the model density is actually discrete.

Assumption 1.3 covers discrete distributions that possess a density w.r.t. the counting measure. The line of arguments as used in the proof of Lemma 2.4 remains intact without change, except for the obvious difference that Ω is a finite (and hence discrete) set now. Likewise, all conclusions drawn from lemma 2.4, including theorem 2.5, as well as the definitions of ordering and topology transfer without change.

2.3 Comparing Discrete and Continuous Distributions

The representation (7) of distributions by the sequence of their moments works even without assuming the density to be continuous. Therefore, it elegantly lets us compare distributions of mixed type, i.e., continuous vs. discrete distributions on a common basis.

It follows that we can – without any changes to the framework – compare discrete to continuous distributions, or any two distributions of the same type in terms of the $\preceq$-, $\prec$- and $\equiv$-relations. This comparison is, obviously, only meaningful if the respective random variables live in the same (metric) space. For example, it would be meaningless to compare ordinal to numeric data.

2.4 Comparing Deterministic to Random

In certain occasions, the consequence of an action may result in perfectly foreseeable effects, such as fines or similar. Such deterministic outcomes can be modeled as degenerate distributions (point- or Dirac-masses)[5]. These are singular and thus violate assumption 1.3, since there is no density function associated with them, unless one is willing to resort to generalized functions; which we do not do in this report. Nevertheless, it is possible to work out the representation in terms of moment sequences. If X is a random variable that deterministically takes on the constant value a all the time, then the respective moment sequence has elements $\mathrm{E}(X^k) = \mathrm{E}(a^k) = a^k$ for all $k \in \mathbb{N}$. Given another non-degenerate distribution with density function f, supported on $\Omega = [0, b]$, we can lower- or upper-bound the moments of the respective random variable Y by exponential functions in k, which can straightforwardly $\preceq$-, $\equiv$- or $\prec$-compared to the representative $(a^k)_{k\in\mathbb{N}}$ of the (deterministic) outcome $a \in \mathbb{R}$. Algorithmic details will follow in part two of this research report.

2.5 Extensions: Relaxing Assumption 1.3

Risk management is often required to handle or avoid extreme (catastrophic) events. The respective statistical models are distributions with so-called "heavy", "long" or "fat" tails (exact definitions and distribution models will follow in part two of this report). Extreme-value distributions such as the Gumbel-distribution, or also the Cauchy-distribution (that is not an extreme value model) are two natural examples that fall into the class of distributions that assign unusually high likelihood to large outcomes (that may be considered as catastrophic consequences of an action). In any case, our assumption 1.3 rules out such distributions by requiring compact support. Even worse, the $\prec$-relation based on the representation of a distribution by the sequence of its moments cannot be extended to cover distributions with heavy tails, as those typically do not have finite moments or moment-generating functions. Nevertheless, such distributions are important tools in risk management.

Things are, however, not drastically restricted by assumption 1.3, for at least two reasons: First, compactness of the support is not necessary for all moments to exist, as the Gaussian distribution has moments of all orders and is supported on the entire real line (thus violating even two of the three conditions of assumption 1.3). Still, it is characterized entirely by its first two moments, and thus can easily be compared in terms of the $\prec$-relation.

Second, and more importantly, any distribution with infinite support can be approximated by a truncated distribution. Given a random variable X with distribution function F, then the *truncated distribution* is the distribution of X conditional on X falling into

[5]Note that the canonic embedding of the reals within the hyperreals represents a number $a \in \mathbb{R}$ by the constant sequence $(a, a, \ldots)$. Picking up this idea would be critically flawed in our setting, as any such constant sequence would be preferred over any probability distribution (whose moment sequence diverges and thus overshoots a inevitably and ultimately).

a finite range, i.e., the truncated distribution function $\hat{F}$ gives the conditional likelihood

$$\hat{F}(x) = \Pr(X \leq x | a \leq X \leq b).$$

Provided that F has a density function f, the truncated density function is

$$\hat{f}(x) = \begin{cases} \frac{f(x)}{F(b)-F(a)}, & a \leq x \leq b; \\ 0, & \text{otherwise.} \end{cases}$$

In other words, we simply crop the density f outside the interval $[a, b]$ and re-scale the resulting function to become a probability distribution again.

Since every distribution function F is non-decreasing and satisfies $\lim_{x\to\infty} F(x) = 1$, any choice of $\delta > 0$ admits a value b such that $F(b) > 1-\delta$. Moreover, since our random variables are all non-negative, we have $\lim_{x\to 0^+} F(x) = \lim_{x\to 0^+} \int_0^x f(x)dx = 0$, since F is right-continuous. It follows that the truncated distribution density for variables of interest in our setting simplifies to $\hat{f}(x) = f(x)/F(b)$. Now, let us compare a distribution F to its truncated version $\hat{F}$ in terms of the probabilities that we would compute:

$$\begin{aligned} \left|F(x) - \hat{F}(x)\right| &= \left|\int_0^x f(t)dt - \int_0^x f(t)/F(b)dt\right| \\ &= \Big| \int_0^x f(t) \underbrace{\left(1 - \frac{1}{F(b)}\right)}_{<\varepsilon} dt\Big| < \varepsilon \int_0^\infty f(t)dt = \varepsilon, \end{aligned}$$

for sufficiently large b, which depends on the chosen $\varepsilon > 0$ that determines the quality of approximation. Conversely, can find always find a truncated distribution $\hat{F}$ that approximates F up to an arbitrary precision $\varepsilon > 0$. This shows that restricting ourselves to distributions with compact support, i.e., adopting assumption 1.3, causes no more than a numerical error that can be made as small as we wish.

More interestingly, we could attempt to play the same trick as before, and characterize a distribution with fat, heavy or long tails by a sequence of approximations to it, arising from better and better precisions $\varepsilon \to 0$. In that sense, we could hope to compare approximations rather than the true density in an attempt to extend the preference and equivalence relations $\preceq$ and $\equiv$ to distributions with fat, heavy or long tails.

Unfortunately, such hope is wrong, as a distribution is not uniquely characterized by a general sequence of approximations (i.e., we cannot formulate an equivalent to lemma 2.3), and the outcome of a comparison of approximations is not invariant to how the approximations are chosen (i.e., there is also no alike for lemma 2.4). To see the latter, take the quantile function $F^{-1}(\alpha)$ for a distribution F, and consider the tail quantiles $\overline{F}^{-1}(\alpha) = F^{-1}(1-\alpha)$. Pick any sequence $(\alpha_n)_{n\to\infty}$ with $\alpha_n \to 0$. Since $\lim_{x\to\infty} F(x) = 1$, the tail quantile sequence behaves like $\overline{F}^{-1}(\alpha_n) \to \infty$, where the limit is independent of the particular sequence $(\alpha_n)_{n\to\infty}$, but only the speed of divergence is different for distinct sequences.

Now, let two distributions F_1, F_2 with infinite support be given. Fix two sequences α_n and ω_n, both vanishing as $n \to \infty$, and set

$$a_n := \overline{F}_1^{-1}(\alpha_n) \leq b_n := \overline{F}_2^{-1}(\omega_n). \tag{11}$$

Let us approximate F_1 by a sequences of truncated distributions $\hat{f}_{1,n}$ with supports $[0, a_n]$ and let the sequence $\hat{f}_{2,n}$ approximate f_2 on $[0, b_n]$. Since $a_n \leq b_n$ for all n, the proof of lemma 2.4 then implies that the approximations with support $[0, a_n]$ is always strictly preferable to the distribution with support $[0, b_n]$, thus $\hat{f}_{1,n} \preceq \hat{f}_{2,n}$. However, by replacing the "$\leq$" by a "$\geq$" in (11), we can construct approximations to F_1, F_2 whose supports exceed one another in the reverse way, so that the approximations would always satisfy $\hat{f}_{1,n} \succeq \hat{f}_{2,n}$. It follows that the sequence of approximations *cannot* be used to unambiguously compare distributions with infinite support, unless we impose some constraints on the tails of the distributions and the approximations. The next lemma assumes this situation to simply not occur, which allows to give a *sufficient* condition to unambiguously extend strict preference in the way we wish.

Lemma 2.7 *Let F_1, F_2 be two distributions supported on the entire nonnegative real half-line $\mathbb{R}^+$ with continuous densities f_1, f_2. Let $(a_n)_{n\in\mathbb{N}}$ be an arbitrary sequence with $a_n \to \infty$ as $n \to \infty$, and let $\hat{f}_{i,n}$ for $i = 1, 2$ be the truncated distribution f_i supported on $[0, a_n]$.*

If there is a constant $c < 1$ and a value $x_0 \in \mathbb{R}$ such that $f_1(x) < c \cdot f_2(x)$ for all $x \geq x_0$, then there is a number N such that all approximations $\hat{f}_{1,n}, \hat{f}_{2,n}$ satisfy $\hat{f}_{1,n} \prec \hat{f}_{2,n}$ whenever $n \geq N$.

Proof. Throughout the proof, let $i \in \{1, 2\}$. The truncated distribution density that approximates f_i is $f_i(x)/(F_i(a_n) - F_i(0))$, where $[0, a_n]$ is the common support of n-th approximation to f_1, f_2. By construction, $a_{n,i} \to \infty$ as $n \to \infty$, and therefore $F_i(a_n) - F_i(0) \to 1$ for $i = 1, 2$. Consequently,

$$Q_n = \frac{F_1(a_n) - F_1(0)}{F_2(a_n) - F_2(0)} \to 1, \qquad \text{as } n \to \infty,$$

and there is an index N such that $Q_n > c$ for all $n \geq N$. In turn,

$$f_2(x) \cdot Q_n > f_2(x) \cdot c > f_1(x),$$

and by rearranging terms,

$$\frac{f_1(x)}{F_1(a_n) - F_1(0)} < \frac{f_2(x)}{F_2(a_n) - F_2(0)}, \tag{12}$$

for all $x \geq x_0$ and all $n \geq N$. The last inequality (12) lets us compare the two approximations easily by the same arguments as have been used in the proof of lemma 2.4, and the claim follows. □

By virtue of lemma 2.7, we can extend the strict preference relation to distributions that satisfy the hypothesis of the lemma but need not have compact support anymore. Precisely, we would strictly prefer one distribution over the other, if all truncated approximations are ultimately preferable over one another.

Definition 2.8 (Extended Preference Relation $\prec$) *Let F_1, F_2 be distribution functions of nonnegative random variables that have infinite support and continuous density functions f_1, f_2. We* (strictly) prefer *F_1* over *F_2, denoted as $F_1 \prec F_2$, if for every sequence $a_n \to \infty$ there is an index N so that the approximations $\hat{F}_{i,n}$ for $i = 1, 2$ satisfy $\hat{F}_{1,n} \prec \hat{F}_{2,n}$ whenever $n \geq N$.*

The $\succ$-relation is defined alike, i.e., the ultimate preference of F_2 over F_1 on any sequence of approximations.

Definition 2.8 is motivated by the above arguments on comparability on common supports, and lemma 2.7 provides us with a handy criterion to decide the extended strict preference relation.

Example 2.9 *It is a simple matter to verify that any two out of the three kinds of extreme value distributions (Gumbel, Frechet, Weibull) satisfy the above condition, thus are strictly preferable over one another, depending on their particular parametrization.*

Definition 2.8 can, however, not applied to every pair of distributions, as the following example shows.

Example 2.10 *Take the "Poisson-like" distributions with parameter $\lambda > 0$,*

$$f_1(k) \propto \begin{cases} \frac{\lambda^{k/2}}{(k/2)!} e^{-\lambda}, & \textit{when } k \textit{ is even;} \\ 0, & \textit{otherwise.} \end{cases}, \quad f_2(k) \propto \begin{cases} 0, & \textit{when } k \textit{ is even;} \\ \frac{\lambda^{(k-1)/2}}{((k-1)/2)!} e^{-\lambda}, & \textit{otherwise} \end{cases}$$

It is a simple matter to verify that no constant $c < 1$ can ever make $f_1 < c \cdot f_2$ and that all moments exist. However, neither distribution is preferable over the other, since finite approximations based on the sequence $a_n := n$ will yield alternatingly preferable approximations.

An occasionally simpler condition that implies the hypothesis of definition 2.8 is

$$\lim_{x \to \infty} \frac{f_1(x)}{f_2(x)} = 0. \tag{13}$$

The reason is simple: if the condition of definition 2.8 were violated, then there is an infinite sequence $(x_n)_{n \in \mathbb{N}}$ for which $f_1(x_n) \geq c \cdot f_2(x_n)$ for all $c < 1$. In that case, there is a subsequence $(x_{n_k})_{k \in \mathbb{N}}$ for which $\lim_{k \to \infty} f_1(x_{n_k})/f_2(x_{n_k}) \geq c$. Letting $c \to 1$, we can construct a further subsequence of $(x_{n_k})_{k \in \mathbb{N}}$ to exhibit that $\limsup_{n \to \infty}(f_1(x_n)/f_2(x_n)) = 1$, so that condition (13) would be refuted.

Remark 2.11 *It must be emphasized that the above line of arguments does not provide us with a mean to extend the $\preceq$- or $\equiv$-relations accordingly. For example, an attempt to define $\preceq$ and $\equiv$ as above is obviously doomed to failure, as asking for two densities f_1, f_2 to satisfy $f_1(x) \leq c_1 \cdot f_2(x)$ ultimately (note the intentional relaxation of $<$ towards $\leq$), and $f_2(x) \leq c_2 \cdot f_1(x)$ ultimately for two constants $c_1, c_2 < 1$ is nonsense.*

A straightforward extension of $\preceq$ can be derived from (based on) the conclusion of lemma 2.7:

Definition 2.12 *Let F_1, F_2 be two distributions supported on the entire nonnegative real half-line $\mathbb{R}^+$ with continuous densities f_1, f_2. Let $(a_n)_{n\in\mathbb{N}}$ be a diverging sequence towards ∞, and let $\hat{F}_{i,n}$ for $i = 1, 2$ denote the density F_i truncated to have support $[0, a_n]$. We define $F_1 \preceq F_2$ if and only if for every sequence $(a_n)_{n\in\mathbb{N}}$ there is some index N so that $\hat{F}_{1,n} \preceq \hat{F}_{2,n}$ for every $n \geq N$.*

More compactly and informally spoken, definition 2.12 demands preference on all approximations with finite support except for at most finitely many exceptions near the origin.

Obviously, preference among distributions with finite support implies the extended preference relation to hold in exactly the same way (since the sequence of approximations will ultimately become constant when a_n overshoots the bound of the support), so definition 2.12 extends the $\preceq$-relation in this sense. This observation justifies our choice of definition 2.12 as a valid extension of $\preceq$ from distributions with compact support to those with infinite support.

Unfortunately, the extended $\preceq$-relation is not as easy to decide as (extended) strict preference and usually calls for computing the moment sequences analytically to be able to compare them in the long run.

Nevertheless, example 2.9 substantiates the expectation that practically relevant distributions over $\mathbb{R}^+$ may indeed compare w.r.t. $\prec$ or $\succ$ by definition 2.8 or criterion (13). While it is easy to exhibit distributions with infinite support that $\prec$-compare in the sense of definition 2.12, their practical relevance or even occurrence is not guaranteed. Example 2.13, however, shows that preference relations are indeed non-empty if they are defined like (9) for distributions with infinite support.

Example 2.13 *Let $X \sim F_1$ be a Gaussian random variable. It is well-known that any Gaussian distribution has finite moments of all orders, so let us call the resulting sequence $(a_n)_{n\in\mathbb{N}}$. Furthermore, let us construct another sequence $(b_n)_{n\in\mathbb{N}}$ being identical to $(a_n)_{n\in\mathbb{N}}$, except for finitely many indices $I = \{i_1, i_2, \ldots, i_k\}$ for which we choose $b_j < a_j$ whenever $j \in I$ and $b_j := a_j$ otherwise. It is easy to see that the existence of the moment-generating function μ_X for F_1 implies the existence of a moment-generating function μ_Y for a random variable $Y \sim F_2$ that has moment sequence $(b_n)_{n\in\mathbb{N}}$ (since the power-series μ_X dominates the series μ_Y). However, it is equally clear that $\mu_X \neq \mu_Y$. Thus, $F_1 \neq F_2$ although $F_1 \equiv F_2$, since the mismatch is only on finitely many indices, and the complement set of these must be in the ultrafilter.*

2.6 Interpretation and Implications of Preferences

Having defined preferences among probability distributions, we now look at what $F_1 \preceq F_2$ actually means. A simple first impression is gained by considering cases in which the first few moments match. For that sake, let F_1, F_2 be two distributions for which no preference has been determined so far.

- If $\mu_1 = \mathrm{E}(F_1) < \mu_2 = \mathrm{E}(F_2)$, then we prefer the distribution with smaller mean. That is, decisions that yield to less average risk would be $\preceq$-preferred.
- If the means are equal, then we prefer whatever distribution has smaller second moment (by virtue of Steiner's theorem). In other words, the preferred among F_1, F_2 would be the one with smaller variance, or otherwise said, the distribution whose outcome is "more predictable" in the sense of varying less around its mean.
- Upon equal mean and variance, the third moment would make the difference. This moment is the *skewness*, and we would prefer the distribution for which $\mathrm{E}\big(X^3\big)$ is smaller, i.e., the distribution that "leans more to the left". The respective distribution would assign more likelihood to smaller outcomes, thus giving less risk.

We refrain here from extending the above intuitions to cover cases when kurtosis tips the scale, as the physical meaning of this quantity is debatable and no consensus among statisticians exists so far. Instead, we give the following result that makes the above intuitions more explicit in the sense of saying that:

> *If $F_1 \preceq F_2$, then "extreme events" are less likely to occur under F_1 than under F_2.*

The rigorous version of this, which especially clarifies the adjective "extreme", is the following theorem:

Theorem 2.14 *Let $X_1 \sim F_1, X_2 \sim F_2$, where F_1, F_2 satisfy assumption 1.3. If $F_1 \preceq F_2$, then there exists a threshold $x_0 \in supp(F_1) \cup supp(F_2)$ so that for every $x \geq x_0$, we have $\Pr(X_1 > x) \leq \Pr(X_2 > x)$.*

Proof. Let f_1, f_2 be the density functions of F_1, F_2. Call $\Omega = \mathrm{supp}(F_1) \cup \mathrm{supp}(F_2) = [0, a]$ the common support of both densities, and take $\xi = \inf\{x \in \Omega : f_1(x) = f_2(x) = 0\}$. Suppose there were an $\varepsilon > 0$ so that $f_1 > f_2$ on every interval $[\xi - \delta, \xi]$ whenever $\delta < \varepsilon$, i.e., f_1 would be larger than f_2 until both densities vanish (notice that $f_1 = f_2 = 0$ on the right of ξ). Then the proof of lemma 2.4 delivers the argument by which we would find a $K \in \mathbb{N}$ so that $\mathrm{E}\big(X_1^k\big) > \mathrm{E}\big(X_2^k\big)$ for every $k \geq K$, which would contradict $F_1 \preceq F_2$. Therefore, there must be a neighborhood $[\xi - \delta, \xi]$ on which $f_1(x) \leq f_2(x)$ for all $x \in [\xi - \delta, \xi]$. The claim follows immediately by setting $x_0 = \xi - \delta$, since taking $x \geq x_0$, we end up with $\int_x^\xi f_1(t)dt \leq \int_x^\xi f_2(t)dt$, and for $i = 1, 2$ we have $\int_x^\xi f_i(t)dt = \int_x^a f_i(t)dt = \Pr(X_i > x)$. □

Observe that this is compatible with the common goals of statistical risk management [7] in other sectors, such as financial business: the preference-relation $\preceq$ compares the tails of distributions, and optimization w.r.t. $\preceq$ seeks to "push" the mass assigned by a distribution towards lower damages. Essentially, we thus focus on large deviations (damages), which intuitively makes sense, as small deviations from the expected behavior may (most likely) be taken by the system's (designed) natural resilience against distortions.

We close this section by giving a few examples showing graphically how different distributions compare against each other.

Example 2.15 (different mean, same variance) *Consider two Gumbel-distributions* $X \sim F_1 = \mathit{Gumbel}(31.0063, 1.74346)$ *and* $Y \sim F_2 = \mathit{Gumbel}(32.0063, 1.74346)$*, where a density for* $\mathit{Gumbel}(a, b)$ *is given by*

$$f(x|a,b) = \frac{1}{b} e^{\frac{x-a}{b} - e^{\frac{x-a}{b}}},$$

where $a \in \mathbb{R}$ *and* $b > 0$ *are the location and scale parameter.*

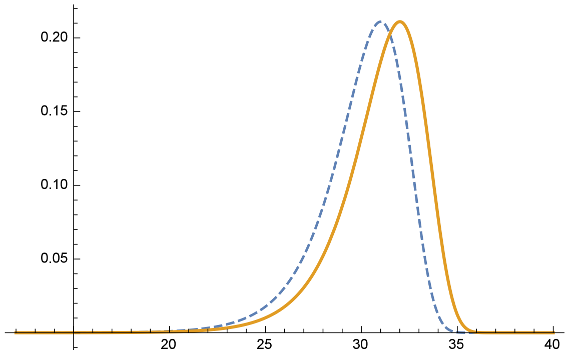

Figure 2: Comparing distributions with different means

Computations reveal that under the given parameters, the means are $\mathrm{E}(X) = 30, \mathrm{E}(Y) = 31$ *and* $\mathrm{Var}\,(X) = \mathrm{Var}\,(Y) = 5$*. Figure 2 plots the respective densities of* F_1 *(dashed) and* F_2 *(solid line). The respective moment sequences evaluate to*

$$\mathrm{E}\left(X^k\right) = (30, 905, 27437.3, 835606, 2.55545 \times 10^7, \ldots),$$
$$\mathrm{E}\left(Y^k\right) = (31, 966, 30243.3, 950906, 3.00162 \times 10^7, \ldots),$$

thus showing that $F_1 \preceq F_2$*. This is consistent with the intuition that the preferred distribution gives* less expected damage.

Example 2.16 (same mean, different variance) *Let us now consider two Gumbel-distributions* $X \sim F_1 = \mathit{Gumbel}(6.27294, 2.20532)$ *and* $Y \sim F_2 = \mathit{Gumbel}(6.19073, 2.06288)$*, for which* $\mathrm{E}(X) = \mathrm{E}(Y) = 5$ *but* $\mathrm{Var}\,(X) = 8 > \mathrm{Var}\,(Y) = 7$*.*

Figure 3 plots the respective densities of F_1 *(dashed) and* F_2 *(solid line). The respective moment sequences evaluate to*

$$\mathrm{E}\left(X^k\right) = (5, 33, 219.215, 1654.9, 11957.8, \ldots),$$
$$\mathrm{E}\left(Y^k\right) = (5, 32, 208.895, 1517.51, 10806.8, \ldots),$$

thus showing that $F_2 \preceq F_1$*. This is consistent with the intuition that among two actions leading to the same expected loss, the preferred one would be one for which the variation around the mean is smaller; thus the loss prediction is "more stable".*

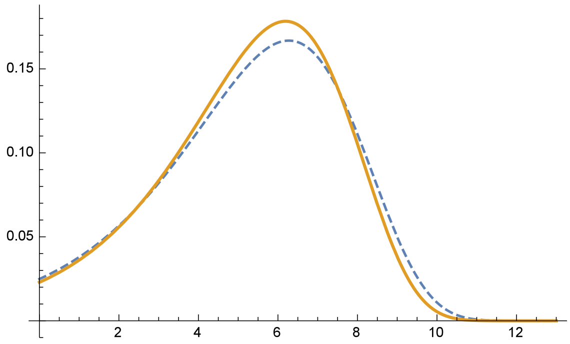

Figure 3: Comparing distributions with equal means but different variance

Example 2.17 (different distributions, same mean and variance) *Let us now consider a situation in which the expected loss (first moment) and variation around the mean (second moment) are equal, but the distributions are different in terms of their shape. Specifically, let* $X \sim F_1 = Gamma(260.345, 0.0373929)$ *and* $Y \sim Weibull(20, 10)$*, with densities as follows:*

$$f_{Gamma}(x|a,b) = \begin{cases} \frac{b^{-a}x^{a-1}e^{-\frac{x}{b}}}{\Gamma(a)}, & x > 0; \\ 0, & otherwise \end{cases}$$

$$f_{Weibull}(x|a,b) = \begin{cases} \frac{ae^{-\left(\frac{x}{b}\right)^a}\left(\frac{x}{b}\right)^{a-1}}{b}, & x > 0; \\ 0, & otherwise \end{cases}$$

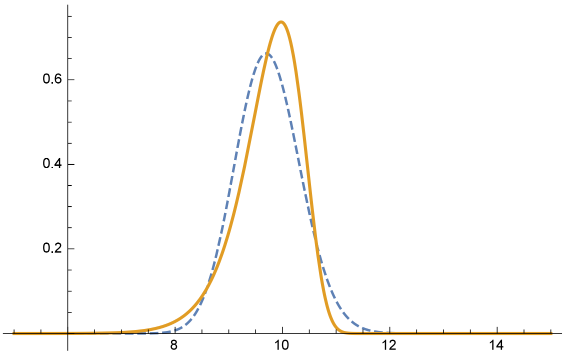

Figure 4: Comparing distributions with matching first two moments but different shapes

Figure 4 plots the respective densities of F_1 *(dashed) and* F_2 *(solid line). The respective moment sequences evaluate to*

$$E\left(X^k\right) = (9.73504, 95.1351, 933.259, 9190.01, 90839.7, \ldots),$$
$$E\left(Y^k\right) = (9.73504, 95.1351, 933.041, 9181.69, 90640.2, \ldots),$$

thus showing that $F_2 \preceq F_1$. In this case, going with the distribution that visually "'leans more towards lower damages" would be flawed, since F_1 nonetheless assigns larger likelihood to larger damages. The moment sequence, on the contrary, unambiguously points out F_2 as the preferred distribution. This illustrates Theorem 2.14.

3 Games with Uncertain Payoffs

Given a total ordering $\preceq$ on the set of actions as defined in section 2, we can go on lifting the remaining concepts and results of game theory to our new setting. In particular, we will have to investigate zero-sum competitions and Nash-equilibria in games whose payoffs are probability distributions. Before, however, it pays to look at arithmetics in our chosen subset of hyperreal numbers that represent our payoff distributions. It turns out that things cannot be straightforwardly be carried over, as we will illustrate in the next section.

3.1 Arithmetic in $\mathfrak{F} \subset \mathbb{R}^\infty/\mathcal{U}$

The space $\mathbb{R}^\infty/\mathcal{U}$ is elsewhere known as the set of *hyperreal numbers*. Together with the ordering relation defined in the same way as (9), and because $\mathcal{U}$ is an ultrafilter, $\mathfrak{F}$ is actually a field, and in many ways behaves like the real numbers. This is due to the ultrafilter acts in much the same way as a maximal ideal, when the quotient structure is formed. For example, we can soundly define min- and max-operators on $\mathfrak{F}$. Furthermore, we can add and subtract elements from $\mathfrak{F}$ in the canonical way by applying the respective operation pairwise on the sequences' elements. Likewise, we can define an absolute-value function $|x| = (|x_i|)_{i\in\mathbb{N}}$ on the sequences, which naturally satisfies the triangle inequality because the sequence's elements are from $\mathbb{R}$. However, we stress that the absolute value does not induce a metric on $\mathfrak{F}$ (even though it satisfies the necessary conditions), as the absolute value under this definition is not real-valued. This is one difference to the field $\mathbb{R}$.

A more important difference is the observation that any probability distribution satisfying assumption 1.3 can be represented by an element in $\mathfrak{F}$, but the converse is not true! For instance, given $F \in \mathfrak{F}$ as a representative of some probability distribution, the element $(-F)$ as being the sequence of moments of F, only with negative signs, does not represent a distribution (in general, and specifically under assumption 1.3). Neither is the sum of two moments necessarily the moment of some other probability distribution. Finally, observe that the zero element $0 = (0, 0, \ldots)$ does not define a proper probability distribution. Hence, the concept of a "zero-sum game" must be replaced by the (strategically equivalent) concept of a constant-sum game, to properly define things. This issue will not be of any particular importance in the following.

Our proofs will nevertheless heavily rely on the existence of a well-defined ordering and arithmetic on the subset $\mathfrak{F}$ of the hyperreals. The fact that in lack of an explicit representation of $\mathcal{U}$ we cannot do arbitrary arithmetic somewhat limits the candidate algorithms to analyze the games and compute equilibria and security strategies, however, this limitation is not severe and can be overcome in our context of application.

3.2 Continuity of $F(\boldsymbol{p},\boldsymbol{q})(r)$ in $(\boldsymbol{p},\boldsymbol{q})$

The existence of Nash-equilibria crucially hinges on the continuity of payoff functionals (in the classical setting). The existence of a topology on the hyperreal set that we consider lets us soundly define continuity in terms of the topology, but proving our payoff distribution function (3) to be continuous is so far an open issue, and this gap shall be closed now.

To establish continuity of the distribution-valued utility function $u(\boldsymbol{p},\boldsymbol{q}) := F(\boldsymbol{p},\boldsymbol{q})$ we have to show that any set in the topology $\mathcal{T}$, i.e., any open set in $\mathfrak{F}$, has a preimage under u that is open in $S_1 \times S_2$ w.r.t. the product topology. The following lemma establishes the important steps towards this conclusion by exploiting the ordering and arithmetic within $\mathfrak{F}$. Hereafter, we consider $(\mathfrak{F} \subset \mathbb{R}^\infty/\mathcal{U}, \mathcal{T}, \prec)$ as an ordered topological space induced by an arbitrary ultrafilter $\mathcal{U}$.

Lemma 3.1 *Let $r_1,\ldots,r_k \in \mathfrak{F}$ for $k \geq 1$ be a set of fixed elements, and take $\boldsymbol{\alpha} = (\alpha_1,\ldots,\alpha_k) \in \mathbb{R}^k$. If two elements $\ell, u \in \mathfrak{F}$ bound the weighted sum $\ell \prec \sum_{i=1}^k \alpha_i r_i = \boldsymbol{\alpha}^T\boldsymbol{r} \prec u$, then there is some strictly positive $\delta \in \mathbb{R}$ so that $\ell \prec \widetilde{\boldsymbol{\alpha}}^T\boldsymbol{r} \prec u$ for every $\widetilde{\boldsymbol{\alpha}}$ within a δ-neighborhood of $\boldsymbol{\alpha}$ in $\mathbb{R}^k$.*

Proof. Define $\Delta := \min\left\{\boldsymbol{\alpha}^T\boldsymbol{r} - \ell, u - \boldsymbol{\alpha}^T\boldsymbol{r}\right\} \succ 0$ and $r := \max\{r_1,\ldots,r_k\}$. Suppose that we would modify all weights α_i to $\alpha_i + \delta_i = \widetilde{\alpha}_i$. If so, then the so-modified sum differs from the given one by $\left|\widetilde{\boldsymbol{\alpha}}^T\boldsymbol{r} - \boldsymbol{\alpha}^T\boldsymbol{r}\right| \leq \sum_{i=1}^k |\delta_i|\, r_i \leq r \cdot \sum_{i=1}^k |\delta_i|$. Now, suppose that all $|\delta_i| \leq \delta$, then the change alters $\boldsymbol{\alpha}^T\boldsymbol{r}$ by a magnitude of no more than $r \cdot \sum_{i=1}^k \delta_i \leq r \cdot k \cdot \delta$. As k and r are fixed, we can choose δ sufficiently small to satisfy $r \cdot k \cdot \delta \prec \Delta$, in which case we must have $\left|\widetilde{\boldsymbol{\alpha}}^T\boldsymbol{r} - \boldsymbol{\alpha}^T\boldsymbol{r}\right| < \Delta$, and therefore $\ell \prec \widetilde{\boldsymbol{\alpha}}^T\boldsymbol{r} \prec u$ for any choice of $\widetilde{\alpha}$ within an δ-neighborhood of $\boldsymbol{\alpha}$ in the maximum-norm on $\mathbb{R}^k$. □

By virtue of lemma 3.1, continuity of $F(\boldsymbol{p},\boldsymbol{q})$ is easily implied by the continuity of the weights $C_{\boldsymbol{p},\boldsymbol{q}}(i,j)$ in $(\boldsymbol{p},\boldsymbol{q})$.

Proposition 3.2 *Let i,j be integers and define the function $D_{ij} : S_1 \times S_2 \to \mathbb{R}$ as $D_{ij}(\boldsymbol{p},\boldsymbol{q}) = C_{\boldsymbol{p},\boldsymbol{q}}(i,j) = \Pr_{\boldsymbol{p},\boldsymbol{q}}(i,j)$. If D_{ij} is continuous and all F_{ij} satisfy assumption 1.3, then the mapping $F : S_1 \times S_2 \to \mathfrak{F}$; $(\boldsymbol{p},\boldsymbol{q}) \mapsto \sum_{i,j} C_{\boldsymbol{p},\boldsymbol{q}}(i,j) F_{ij}$ is continuous w.r.t. the product topology on $S_1 \times S_2$ and the order topology on $\mathfrak{F}$.*

Proof. Without a metric on $\mathfrak{F}$, we need to show that the preimage of every open set in $\mathfrak{F}$ under F is open to prove that F is continuous. For that sake, let the open set $(\ell,u) \in \mathcal{T}$ be arbitrary and contain some point $F(\boldsymbol{p},\boldsymbol{q})$ (which must exist, for otherwise, the set of preimages would be empty). To ease notation, let us flatten the double-sum $\sum_{i,j}$ into an ordinary sum (say, by introducing a multiindex ν) over $k = n \cdot m$ elements, where n, m are the limits in the original expression. Then, the mapping takes the form $F(\boldsymbol{p},\boldsymbol{q}) = \sum_{\nu=1}^k D_\nu(\boldsymbol{p},\boldsymbol{q}) F_\nu$. With the weights $\boldsymbol{\alpha}$ being defined by the individual values of $D_\nu(\boldsymbol{p},\boldsymbol{q}) = C_{\boldsymbol{p},\boldsymbol{q}}(\nu)$, we can apply lemma 3.1 to establish a bound $\delta > 0$ within which we can arbitrarily alter the weights towards $\widetilde{\boldsymbol{\alpha}}$ without leaving the open set (ℓ,u). Since C is continuous on compact $S_1 \times S_2$ it is also uniformly continuous, and we can fix a

$\delta' > 0$ so that $\|D_\nu(\boldsymbol{p}', \boldsymbol{q}') - D_\nu(\boldsymbol{p}, \boldsymbol{q})\| < \delta$ whenever $\|(\boldsymbol{p}, \boldsymbol{q}) - (\boldsymbol{p}', \boldsymbol{q}')\| < \delta'$, independently of the particular point $(\boldsymbol{p}, \boldsymbol{q})$. The sought pre-image of the open set (ℓ, u) is thus the (infinite) union of open neighborhoods constructed in the way described, and thus itself open. □

3.3 Security Strategies and Zero-Sum Games

Given the continuity and ordering of payoffs in games that reward players with random variables, our next step is the definition of zero-sum games in this context. We rephrase the standard definition of a zero-sum equilibrium using the preference relation $\preceq$ in the straightforward fashion, by defining a two-person game $\Gamma_0 = (\{1, 2\}, \{S_1, S_2\}, \{F, -F\})$ as usual, but keeping the following in mind:

- When $(a_i)_{i=1}^\infty$ defines the probability distribution F, then $-F$ is defined by $(-a_i)_{i=1}^\infty$, but not necessarily defines a valid probability distribution any more. To see this, simply recall that the Taylor-series (6) would upon all negative moments define a negative-valued function $\mu_X(s) < 0$, which cannot be a moment-generating function, since $\mu_X(s) = \mathrm{E}\big(e^{sX}\big) \geq 0$ in any case.
- The sum $F + (-F)$ being computed in $\mathbb{R}^\infty/\mathcal{U}$ is defined as the sequence that is constantly zero. Again, this does not define a probability distribution in the proper sense. However, strategic equivalence (as in the classical theory of games) tells the set of equilibria does not change if the payoffs of both players get a constant value added to them (in that case, the payoffs on either side are changed by the same value, leaving all inequalities intact). By the same token, we may think of constant-sum games, which avoid degenerate cases as above (where two distributions add up to something that is no longer a distribution).

The familiar equilibrium condition in a two-player zero-sum game Γ can be rephrased as follows: a strategy profile $(\boldsymbol{p}^*, \boldsymbol{q}^*) \in S_1 \times S_2$ is a *(Nash-)equilibrium*, if for every $(\boldsymbol{p}, \boldsymbol{q}) \in S_1 \times S_2$,

$$F(\boldsymbol{p}, \boldsymbol{q}^*) \preceq F(\boldsymbol{p}^*, \boldsymbol{q}^*) \preceq F(\boldsymbol{p}^*, \boldsymbol{q}), \tag{14}$$

i.e., any deviation from the optimal profile $(\boldsymbol{p}^*, \boldsymbol{q}^*)$ would worsen the situation of either player (in either a zero- or constant-sum competition).

Before security strategies can be defined properly, we need to assure existence of equilibria profiles in our modified setting. In this regard, Glicksberg's theorem, which generalizes Nash's original theorem, helps out:

Theorem 3.3 (see [4] and [3, Theorem 1.3]) *Consider a strategic-form game whose strategy spaces S_i are nonempty compact subsets of a metric space. If the payoff functions are continuous w.r.t. the metric, then there exists a Nash-equilibrium in mixed strategies.*

It is a simple matter to verify that

- both sets PS_1, PS_2 are finite subsets of $\mathbb{R}$ (or $\mathbb{R}^d$ for $d > 1$) and hence compact w.r.t. all norms on this Euclidian space, and
- the payoff functions are continuous by lemma 3.2,

so that a Nash-equilibrium in the sense of (14) exists by theorem 3.3.

Furthermore, any saddle-point satisfying (14) defines the same payoff distribution in the sense of possibly defining a different representative but in any case pinning down the same equivalence class of distributions in $\mathbb{R}^\infty/\equiv$, where $\equiv$ is defined by (10). The proof is a restatement of Theorem 3.12 in [11].

Lemma 3.4 *Let a continuous function $F : PS_1 \times PS_2 \to \mathfrak{F}$ be given, where $PS_1 \subseteq \mathbb{R}^{n_1}, PS_2 \subseteq \mathbb{R}^{n_2}$. Furthermore, let $(\boldsymbol{p}', \boldsymbol{q}')$ and $(\boldsymbol{p}^*, \boldsymbol{q}^*)$ be two different saddle-points . Then, $(\boldsymbol{p}^*, \boldsymbol{q}')$ and $(\boldsymbol{p}', \boldsymbol{q}^*)$ are also saddle-points, and*

$$F(\boldsymbol{p}', \boldsymbol{q}') \equiv F(\boldsymbol{p}^*, \boldsymbol{q}^*) \equiv F(\boldsymbol{p}^*, \boldsymbol{q}') \equiv F(\boldsymbol{p}', \boldsymbol{q}^*).$$

Proof. The proof is by direct checking of the saddle-point condition, i.e.,

$$\begin{aligned} F(\boldsymbol{p}^*, \boldsymbol{q}') \preceq F(\boldsymbol{p}', \boldsymbol{q}') \preceq F(\boldsymbol{p}', \boldsymbol{q}^*) \preceq F(\boldsymbol{p}^*, \boldsymbol{q}^*) \preceq F(\boldsymbol{p}^*, \boldsymbol{q}') \\ \Rightarrow F(\boldsymbol{p}', \boldsymbol{q}') \equiv F(\boldsymbol{p}^*, \boldsymbol{q}^*) \equiv F(\boldsymbol{p}', \boldsymbol{q}^*) \equiv F(\boldsymbol{p}^*, \boldsymbol{q}'). \end{aligned}$$

(p', q^*) is saddle-point,

$$F(\boldsymbol{p}, \boldsymbol{q}^*) \preceq F(\boldsymbol{p}^*, \boldsymbol{q}^*) \equiv F(\boldsymbol{p}', \boldsymbol{q}^*) \equiv F(\boldsymbol{p}', \boldsymbol{q}') \preceq F(\boldsymbol{p}', \boldsymbol{q}).$$

The fact that $(\boldsymbol{p}^*, \boldsymbol{q}')$ is a saddle-point is proved analogously. □

Lemma 3.4 permits calling $v \equiv F(\boldsymbol{p}^*, \boldsymbol{q}^*)$ *the saddle-point value* of the zero-sum game Γ_0. With this, we are ready to step forward towards defining *security strategies.*

For security strategies in the general case of two-person games with arbitrary payoffs, let us denote the general game by $\Gamma = (\{1,2\}, \{S_1, S_2\}, \{F, G\})$, in which player 1 has payoff structure F, and player 2 has payoff structure G. Let Γ_0 denote the associated zero-sum competition that – adopting a worst-case assumption – substitutes an unknown G by $(-F)$, i.e., $\Gamma_0 = (\{1,2\}, \{S_1, S_2\}, \{F, -F\})$.

Theorem 3.5 *Let Γ be an arbitrary two-person game, and let Γ_0 be its associated zero-sum competition with equilibrium profile $(\boldsymbol{p}^*, \boldsymbol{q}^*)$. Then, for every $(\boldsymbol{p}, \boldsymbol{q}) \in S_1 \times S_2$, we have*

$$v \preceq F(\boldsymbol{p}, \boldsymbol{q}), \tag{15}$$

and the strategy $\boldsymbol{q}^*$ achieves equality in (15).

Proof. Observe that the payoff $F(\boldsymbol{p}, \boldsymbol{q})$ is the same for player 1 in both games Γ and Γ_0. So, if player 1 follows an equilibrium profile $(\boldsymbol{p}^*, \boldsymbol{q}^*)$ of Γ_0, then the saddle-point condition (14) yields

$$v \equiv F(\boldsymbol{p}^*, \boldsymbol{q}^*) \preceq F(\boldsymbol{p}^*, \boldsymbol{q}), \tag{16}$$

for every $\boldsymbol{q}$, with equality being achieved by $\boldsymbol{q}^*$, obviously. Since player 2 will play to the best of its own benefit in Γ, call the equilibrium profile in Γ (which exists by theorem 3.3) $(\boldsymbol{p}, \boldsymbol{q})$. In Γ, however, player 1 deviates by playing $\boldsymbol{p}^*$ thus increasing the payoff for player 2. Thus, we can continue inequality (16) on the right side towards

$$F(\boldsymbol{p}^*, \boldsymbol{q}) \preceq F(\boldsymbol{p}, \boldsymbol{q}). \tag{17}$$

The theorem is now immediate from expressions (16) and (17). □

4 Optimizing Multiple Security Goals

In case of multiple goals to be defended, we turn the two conclusions of theorem 3.5 for scalar-valued games into two axioms on vector-valued games. This leads to the following definition from [9]:

Definition 4.1 (Multi-Goal Security Strategy with Assurance)
A strategy $\boldsymbol{p}^* \in S_1$ in a two-person multi-criteria game with continuous payoff $\boldsymbol{u}_1 : S_1 \times S_2 \to \mathfrak{F}^d$ for the service provider (player 1), is called a Multi-Goal Security Strategy with Assurance *(MGSS) with* assurance $\boldsymbol{v} = (V_1, \ldots, V_d) \in \mathfrak{F}^d$ *if two criteria are met:*

Axiom 1: Assurance *The values in $\boldsymbol{v}$ are the component-wise guaranteed payoff for player 1, i.e. for all components i, we have*

$$V_i \preceq u_1^{(i)}(\boldsymbol{p}^*, \boldsymbol{q}) \qquad \forall \boldsymbol{q} \in S_2, \tag{18}$$

with equality being achieved by at least one choice $\boldsymbol{q}_i \in S_2$.

Axiom 2: Efficiency *At least one assurance becomes void if player 1 deviates from $\boldsymbol{p}^*$ by playing $\boldsymbol{p} \neq \boldsymbol{p}^*$. In that case, some $\boldsymbol{q}_{\boldsymbol{p}} \in S_2$ exists (that depends on $\boldsymbol{p}$) such that*

$$\boldsymbol{u}_1(\boldsymbol{p}, \boldsymbol{q}_{\boldsymbol{p}}) \preceq_1 \boldsymbol{v}. \tag{19}$$

4.1 Characterization and Existence of Security Strategies

The existence of MGSS in the sense of definition 4.1 hinges on a few basic facts about continuous real-valued functions. Fortunately, it turns out that the only ingredient needed is uniform continuity of payoffs on compact strategy spaces. The precise fact used to establish the existence of multi-criteria security strategies is the following [9]:

> Let $u_1 : PS_1 \times PS_2 \to \mathbb{R}^d$ be player 1's payoff function, and let it be continuous. Since $PS_1 \times PS_2$ is compact, given any $\varepsilon > 0$, we can find a $\delta > 0$ such that $\|\boldsymbol{u}_1(\boldsymbol{x}, \boldsymbol{y}) - \boldsymbol{u}_1(\boldsymbol{x}', \boldsymbol{y}')\|_\infty < \varepsilon$, whenever $\|\boldsymbol{x} - \boldsymbol{y}\|_\infty < \delta$.

This argument can be transferred easily to our setting, by a simple inspection of the proofs of lemma 3.1 and proposition 3.2.

Proposition 3.2 tells that $F : S_1 \times S_2 \to \mathfrak{F}$ is continuous w.r.t. the topologies on $\mathbb{R}^{|PS_1|\cdot|PS_2|}$ and the ordering topology on $\mathfrak{F}$. So, let $(-\varepsilon, +\varepsilon)$ for $0 \prec \varepsilon \in \mathfrak{F}$ be an open interval, then we can find some real $\delta > 0$ such that whenever $\|(\boldsymbol{p}, \boldsymbol{q}) - (\boldsymbol{p}', \boldsymbol{q}')\|_\infty < \delta$, we have $-\varepsilon \prec F(\boldsymbol{p}, \boldsymbol{q}) - F(\boldsymbol{p}', \boldsymbol{q}') \prec \varepsilon$ by construction of δ (see the proofs of lemma 3.1 and proposition 3.2). More importantly, the δ is constructed only from ε but is independent of $(\boldsymbol{p}, \boldsymbol{q})$. Hence, F is indeed *uniformly continuous*[6]. For vector-valued payoffs $\boldsymbol{F} : (\boldsymbol{p}, \boldsymbol{q}) \mapsto (F^{(1)}(\boldsymbol{p}, \boldsymbol{q}), \ldots, F^{(d)}(\boldsymbol{p}, \boldsymbol{q}))$, uniform continuity is inherited in the canonical way.

Furthermore, we need a proper replacement for the ∞-norm on $\mathbb{R}^d$, which will work on elements $\boldsymbol{x} \in \mathfrak{F}^d$. This replacement is $[\![\boldsymbol{x}]\!]_\infty = [\![(x_1, \ldots, x_d)]\!]_\infty := \max\{|x_1|, \ldots, |x_d|\}$ for $(x_1, \ldots, x_d) \in \mathfrak{F}^d$, which "resembles" the ∞-norm on the real space. The slight difference in the notation shall highlight the fact that $[\![\cdot]\!]_\infty$ is technically *not* a norm, as it maps onto elements of $\mathfrak{F}$ rather than real numbers.

Lemma 4.2 is proved here for the sake of rigor, but is the only part from [9] that requires a reconsideration. The main result needed here will be theorem 4.4, whose proof will then rest on our version of lemma 4.2.

Lemma 4.2 *Let Γ be a multi-criteria game, and let $\boldsymbol{p}^*$ be a multi-goal security provisioning strategy with assurance $\boldsymbol{v}$, assuming that one exists. Then, no vector $\widetilde{\boldsymbol{v}} \prec \boldsymbol{v}$ is an assurance for $\boldsymbol{p}^*$.*

Proof. Let $\widetilde{\boldsymbol{v}} \prec \boldsymbol{v}$, put $\varepsilon := \min_{1\leq i\leq k}\{v_i - \widetilde{v}_i\}$ and observe that $\varepsilon \succ 0$. Since $\boldsymbol{F}$ is uniformly continuous, a $\delta \succ 0$ exists for which $\|(\boldsymbol{p}, \boldsymbol{q}) - (\boldsymbol{p}', \boldsymbol{q}')\|_\infty \prec \delta$ implies $[\![\boldsymbol{F}(\boldsymbol{p}, \boldsymbol{q}) - \boldsymbol{F}(\boldsymbol{p}', \boldsymbol{q}')]\!]_\infty \prec \frac{\varepsilon}{2}$.

Consider the mapping $\boldsymbol{u}_{\boldsymbol{q}} : S_1 \to \mathbb{R}^k, \boldsymbol{u}_{\boldsymbol{q}}(\boldsymbol{p}) := \boldsymbol{F}(\boldsymbol{p}, \boldsymbol{q})$, which is as well uniformly continuous on S_1 by the same argument. So, $\|(\boldsymbol{p}^*, \boldsymbol{q}) - (\boldsymbol{p}', \boldsymbol{q})\|_\infty = \|\boldsymbol{p}^* - \boldsymbol{p}'\|_\infty \prec \delta$ implies $[\![\boldsymbol{u}_{\boldsymbol{q}}(\boldsymbol{p}^*) - \boldsymbol{u}_{\boldsymbol{q}}(\boldsymbol{p}')]\!]_\infty = \max_{1\leq i\leq k}\left|F^{(i)}(\boldsymbol{p}^*, \boldsymbol{q}) - F^{(i)}(\boldsymbol{p}', \boldsymbol{q})\right| \prec \frac{\varepsilon}{2} \quad \forall \boldsymbol{q} \in S_2$. It follows that $\left|F^{(i)}(\boldsymbol{p}^*, \boldsymbol{q}) - F^{(i)}(\boldsymbol{p}', \boldsymbol{q})\right| \prec \frac{\varepsilon}{2}$ for $i = 1, \ldots, k$ and all $\boldsymbol{q} \in S_2$, and consequently $\max_{\boldsymbol{q}\in S_2}\left|F^{(i)}(\boldsymbol{p}^*, \boldsymbol{q}) - F^{(i)}(\boldsymbol{p}', \boldsymbol{q})\right| \prec \frac{\varepsilon}{2}$. Now, selecting any $\boldsymbol{p}' \neq \boldsymbol{p}^*$ within an δ-neighborhood of $\boldsymbol{p}^*$, we end up asserting $F^{(i)}(\boldsymbol{p}', \boldsymbol{q}) \succeq F^{(i)}(\boldsymbol{p}^*, \boldsymbol{q}) - \frac{\varepsilon}{2}$ for every i and $\boldsymbol{q} \in S_2$.

Using $F^{(i)}(\boldsymbol{p}^*, \boldsymbol{q}) \succeq v_i$, we can continue by saying that $F^{(i)}(\boldsymbol{p}', \boldsymbol{q}) \succeq v_i - \frac{\varepsilon}{2} \succ v_i - \varepsilon$. By definition of ε, we have $v_i - \widetilde{v}_i \succeq \varepsilon$, so that $F^{(i)}(\boldsymbol{p}', \boldsymbol{q}) \succ \widetilde{v}_i$ for all i, contradicting (19) if $\widetilde{\boldsymbol{v}}$ were to be a valid assurance vector. □

To compute MGSS, we apply a simple trick: we cast the two-person game in which player 1 pursuits d goals into a $(d+1)$-person game in which player 1 defends himself against d adversaries, each of which refers to a single security goal. The scenario is a "one-against-all" game, for which numerical solution techniques (e.g., fictitious play) are known. This is subject of upcoming companion work.

[6] The definition on topological spaces (without relying on a metric) is the following: a function $f : X \to Y$ is uniformly continuous, if for any neighborhood B of zero in Y, there is a neighborhood A of zero in X so that $x - y \in A$ implies $f(x) - f(y) \in B$. This definition is satsified by our "distribution-valued" function $F : S_1 \times S_2 \to \mathfrak{F}$.

Definition 4.3 (Auxiliary Game) *Let a multiobjective game*

$$\Gamma = (\{1,2\}, \{S_1, S_2\}, \{\boldsymbol{F}_1, \boldsymbol{F}_2\})$$

be given, where player 1 receives $d \geq 1$ outcomes through the (known) payoff $\boldsymbol{F}_1 = (F_1^{(1)}, \ldots, F_1^{(d)})$. Assume $\boldsymbol{F}_2$ to be unknown. We define the $(d+1)$-player multiobjective game $\overline{\Gamma} = (N, S, H)$ as follows:

- *$N := \{0, 1, \ldots, d\}$, is the player set,*
- *$S := \{S_1, S_2, \ldots, S_2\}$ is the strategy multiset containing d copies of S_2 (one for each opponent in N),*
- *the payoffs are*
  - *vector-valued for player 0, who gets*

$$\overline{\boldsymbol{F}}_0(s_0, \ldots, s_d) := (F_1^{(1)}(s_0, s_1), \ldots, F_1^{(d)}(s_0, s_d)),$$

  - *scalar for all opponents $i = 1, 2, \ldots, d$, receiving*

$$\overline{F}_i(s_0, \ldots, s_d) := -F_1^{(i)}(s_0, s_i).$$

The game $\overline{\Gamma}$ is called the auxiliary game *for Γ.*

Theorem 4.4 *Let Γ be a two-player multi-objective game with $d \geq 1$ distribution-valued payoffs. The situation $\boldsymbol{p}^*$ constitutes a network provisioning strategy with assurance $\boldsymbol{v}$ for player 1 in the game Γ, if and only if it is a Pareto-Nash equilibrium strategy for player 0 in the auxiliary $(d+1)$-player game $\overline{\Gamma}$ according to definition 4.3.*

Proof (Sketch). The proof from [9] transfers with obvious changes to our setting, except for the above version of Lemma 4.2 being used in the last step. □

Theorem 4.4 equates the set of multi-goal security strategies to the set of Pareto-Nash equilibria in a conventional game. Existence of such equilibria is assured by the following theorem:

Theorem 4.5 ([6]) *Let $\Gamma = (\{1, \ldots, p\}, \{S_1, \ldots, S_p\}, \{\boldsymbol{F}_1, \ldots, \boldsymbol{F}_p\})$ be a p-player multiobjective game, where $S_1, \ldots, S_p$ are convex compact sets and $\boldsymbol{F}_1, \ldots, \boldsymbol{F}_p$ represent vector-valued continuous payoff functions (where payoff for player i is composed from $r_i \geq 1$ values). Moreover, let us assume that for every $i \in \{1, 2, \ldots, p\}$ each component $F_i^{(k)}(s_1, s_2, \ldots, s_{i-1}, s_i, s_{i+1}, \ldots, s_p), k \in \{1, 2, \ldots, r_i\}$, of the vector function $\boldsymbol{F}_i$ represents a concave function w.r.t. s_i on S_i for fixed $s_1, \ldots, s_{i-1}, s_{i+1}, \ldots, s_p$. Then the multiobjective game Γ has a Pareto-Nash equilibrium.*

It is *almost* straightforward to apply theorem 4.5, since almost all conditions have been verified already: we have $p = 2$ players, whose (vector-valued) payoffs are continuous by proposition 3.2, transferred canonically to the vector-valued case (which means that player 0 in the auxiliary game has $r_0 = d$ payoffs, and every opponent $i = 1, \ldots, d$ has $r_i = 1$ payoff). Likewise, the action spaces PS_1, PS_2 that we consider are finite subsets of $\mathbb{R}$, and hence the simplex of discrete probability distributions S_1, S_2 are compact and convex sets. However, it remains generally open whether or not the payoff functions are concave. Under independent choices of actions – cf. section 2.1 – this is assured, and theorem 4.5 applies. However, if the actions are chosen interdependently, i.e., we have a nontrivial copula modeling the interplay, concavity of the payoffs must be determined upon the explicit structure of (4).

4.2 Relation to Bayesian Decisions

To embed the minimax-like decision finding that we described in a Bayesian framework, recall that a Bayesian decision is one that is optimal w.r.t. the a-posteriori loss-distribution that incorporates all information. Informally spoken, such decisions naturally give rise to minimax-decisions, if the loss-distribution is the least favourable one. Our minimax approach, on the contrary, has the opponent player 2 look exactly for this least favourable distribution, and the zero-sum assumption then implies that a multi-goal security strategy in the sense of 4.1 can be viewed as a Bayesian decision w.r.t. the Pareto-Nash opponent-strategy in the "zero-sum" auxiliary game associated with our multi-criteria competition (cf. theorem 4.5).

A full fledged treatment of Bayesian decision theory can be found in [10]. We abstain from transferring this framework to our setting, as there appears to be no immediate benefit in doing so, due of the inherent lack of information that risk management here. In other words, while Bayesian decisions heavily rely on data, such data is not usually available in the context of security and defenses against unknown attackers. Attacks like eavesdropping are intrinsically unobservable (in most practically relevant cases), and the consequences may be observed delayed and under fuzzyness.

Summing up, minimax decision as we compute them here may indeed be pessimistic relative to a "more informed" Bayesian decision. Under the expected lack of information that risk management often suffers from, however, it is nevertheless the best that we can do (theoretically).

5 Compiling Quantitative Risk Measures

The outcome of the game-theoretic analysis is in any case two-fold, consisting of:

- An optimal choice rule $\boldsymbol{p}^*$ over the set of actions PS_1, and
- An lower-bound distribution V^* (or vector $\boldsymbol{v}$ if we optimize multiple goals as in section 4) for the random payoff that can be obtained from the game. This payoff is optimal in the sense of not being improvable without risking the existence of

an attack strategy that causes greater damage than predicted by V^*. This bound is valid if and only if actions are drawn at random from PS_1 according to the distribution $\boldsymbol{p}^*$.

While the optimal action choice distribution $\boldsymbol{p}^*$ is easy to interpret, compiling crisp risk figures from the payoff value V^* (or a vector $\boldsymbol{v}$ thereof) requires some more thoughts.

A common approach to risk quantisation is by the well-known "rule-of-thumb"

$$\text{risk} = (\text{incident likelihood}) \times (\text{damage caused by the incident}), \tag{20}$$

The beauty of this formula lies in its compatibility with any nominal or numerical scale of likelihoods and damages, while at the same time, it enjoys a rigorous mathematical fundament, part of which is game theory.

Indeed, formula (20) is essentially the *expected value of the loss-distribution* that is specified by the damage potential of all known incidents, together with their likelihoods. The distribution V^* that we obtain from our analysis of games with distribution-valued payoffs is much more general and thus informative: let v be the optimal distribution, then:

- Formula (20) is merely the first moment of V^*, i.e.,

  $$\text{risk} = \text{likelihood} \times \text{damage} = \mathrm{E}(R)\,, \text{ when } R \sim V^*$$

  where the last quantity is equal to $\mathrm{E}(V^*) = \mathrm{E}\big(R^1\big)\,(\boldsymbol{p}^*, \boldsymbol{q}^*)$ that can be computed from equation (5). The missing value $\boldsymbol{q}^*$ is here exactly the optimal strategy for the attacker in the hypothetical zero-sum competition that is set up to compute the sought security strategy $\boldsymbol{p}^*$. In other words, the value $\boldsymbol{q}^*$ is a natural by-product of the computation of $\boldsymbol{p}^*$ and delivered together with it.

- Beyond the crisp result that formula (20) delivers, the distribution V can be analyzed for higher moments too, such as *variance* of the damage, or quantiles that would provide us with probabilistic risk bounds: for example, computing the 5%- and 95%-quantiles of V gives two bounds within the damage will range with a 90% likelihood. This may be another interesting figure for decision support, which cannot be obtained on the classical way via formula (20).

If the results refer to a MGSS, then the above reasoning holds for every component of the assurance vector $\boldsymbol{v} = (V_1^*, \ldots, V_d^*)$. That is, risk figures can be computed independently for every aspect of interest.

Remark 5.1 *The entries in the optimal attack strategy $\boldsymbol{q}^*$ are an optimal choice rule over the set of attacker's actions PS_2. As such, they can be taken as indicators to neuralgic spots in the infrastructure. However, it must be emphasized that equilibria, and hence also security strategies, are notoriously non-unique. Therefore, the indication by $\boldsymbol{q}^*$ is only one among many other possible ones, and thus must not be used isolated from or as a substitute for other/further information and expertise.*

Remark 5.2 *As an alternative quantity of interest, one may ask for the expected maximum repair costs over a duration of (unchanging) infrastructure provisioning* $\boldsymbol{p}$ *and risk situation* $\boldsymbol{q}$*. In adopting such an approach, we can put* $M_n := \max\{R_1, \ldots, R_n\}$*, but then find*

$$\begin{aligned}\Pr(M_n \leq r) &= \Pr(R_1 \leq r, R_2 \leq r, \ldots, R_n \leq r)\\ &= \Pr(R_1 \leq r)\Pr(R_2 \leq r)\cdots\Pr(R_n \leq r) = [(F(\boldsymbol{p}, \boldsymbol{q}))(r)]^n,\end{aligned}$$

if the repairs induce independent costs. Since $(F(\boldsymbol{p}, \boldsymbol{q}))(r) \leq 1$ *for all* r *by definition of a distribution function, we end up concluding that the long-run maximum is either zero or one, as* $[(F(\boldsymbol{p}, \boldsymbol{q}))(r)]^n \to 0$ *if* $(F(\boldsymbol{p}, \boldsymbol{q}))(r) < 1$*, or remains* $F(\boldsymbol{p}, \boldsymbol{q})(r) = 1$ *otherwise.*

So the maximum is not as informative as we may hope under the assumptions made. Nevertheless, modeling maxima is indeed the proper way to control risk, and theorem 2.14 fits our $\preceq$*-relation and framework quite well into these classical line of approaches.*

6 Outlook

So far, various practical issues have been left untouched, which will be covered in companion work to this report. In particular, future discussions will include:

- Methods and models to capture extreme events (distributions commonly used in quantitative risk management)
- Methods and algorithms to compile payoff distributions from simulation or empirical data
- Algorithms to efficiently decide preference and equivalence among probability distributions
- Algorithms to numerically compute security strategies that account for the limited arithmetic that we can do in lack of an explicit model of the hyperreal structure that represents our distributions.

This report is meant to provide the theoretical fundament to build the practical analysis methods that are described in follow-up work. In that sequel to this research, issues of modeling extreme events and damage distributions for a game-theoretic risk control will be discussed.

Acknowledgment

This work was supported by the European Commission's Project No. 608090, HyRiM (Hybrid Risk Management for Utility Networks) under the 7th Framework Programme (FP7-SEC-2013-1). The author is indepted to Sandra König from AIT for valuable discussions and for spotting some errors.

References

[1] Kurt Endl and Wolfgang Luh. *Analysis III. Funktionentheorie, Differentialgleichungen.* 1995.

[2] Michael J. Evans and Jeffrey S. Rosenthal. *Probability and Statistics – The Science of Uncertainty.* W.H. Freeman and Co., 2004.

[3] Drew Fudenberg and Jean Tirole. *Game Theory.* MIT Press, London, 1991.

[4] Irving Leonard Glicksberg. A further generalization of the Kakutani fixed point theorem, with application to nash equilibrium points. In *Proceedings of the American Mathematical Society*, volume 3, pages 170–174, Feb 1952.

[5] Michael A. Golberg. *An Introduction to Probability Theory with Statistical Applications.* Plenum Press, 1984.

[6] D. Lozovanu, D. Solomon, and A. Zelikovsky. Multiobjective games and determining pareto-nash equilibria. *Buletinul Academiei de Stiinte a Republicii Moldova Matematica*, 3(49):115–122, 2005. ISSN 1024-7696.

[7] A. McNeil, Rüdiger Frey, and P. Embrechts. *Quantitative Risk Management – Concepts, Techniques and Tools.* Princeton Univ. Press, 2005.

[8] Roger B. Nelsen. *An Introduction To Copulas.* Lecture Notes in Statistics 139. Springer, 1999.

[9] Stefan Rass. On game-theoretic network security provisioning. *Springer Journal of Network and Systems Management*, 21(1):47–64, 2013.

[10] Christian P. Robert. *The Bayesian choice.* Springer, New York, 2001.

[11] Walter Schlee. *Einführung in die Spieltheorie.* Vieweg, 2004.
