# Supplementary material for: Correction: Decisions with Uncertain Consequences—A Total Ordering on Loss-Distributions
Source: PLoS One. 2025 Sep 22;20(9):e0333014. doi: 10.1371/journal.pone.0333014 (PMC12453220; doi:10.1371/journal.pone.0333014)
Supplement: S1 File — (ZIP) [file pone.0333014.s001.zip › 1511.08591v2.pdf]

arXiv:1511.08591v2 [econ.GN] 9 Apr 2020

On Game-Theoretic Risk Management (Part Two)

Algorithms to Compute Nash-Equilibria in Games with Distributions as Payoffs

Stefan Rass *
stefan.rass@aau.at

April 10, 2020

Abstract

The game-theoretic risk management framework put forth in the precursor work "Towards a Theory of Games with Payoffs that are Probability-Distributions" (arXiv:1506.07368 [q-fin.EC]) is herein extended by algorithmic details on how to compute equilibria in games where the payoffs are probability distributions. Our approach is "data driven" in the sense that we assume empirical data (measurements, simulation, etc.) to be available that can be compiled into distribution models, which are suitable for efficient decisions about preferences, and setting up and solving games using these as payoffs. While preferences among distributions turn out to be quite simple if nonparametric methods (kernel density estimates) are used, computing Nash-equilibria in games using such models is discovered as inefficient (if not impossible). In fact, we give a counterexample in which fictitious play fails to converge for the (specifically unfortunate) choice of payoff distributions in the game, and introduce a suitable tail approximation of the payoff densities to tackle the issue. The overall procedure is essentially a modified version of fictitious play, and is herein described for standard and multicriteria games, to iteratively deliver an (approximate) Nash-equilibrium. An exact method using linear programming is also given.

*Universität Klagenfurt, Institute of Applied Informatics, System Security Group, Universitätsstrasse 65-67, 9020 Klagenfurt, Austria. This work has been done in the course of consultancy for the EU Project HyRiM (Hybrid Risk Management for Utility Networks; see https://hyrim.net), led by the *Austrian Institute of Technology* (AIT; www.ait.ac.at). See the acknowledgement section.

Contents

1 Introduction **3**

2 Comparing Distributions Efficiently **4**
2.1 Estimating Payoff Distributions from Simulations 4
2.2 Comparing Kernel Density Models 5
2.3 Comparing Deterministic to Random Variables 7
2.4 Distributions with Infinite Support 8
2.5 On Paradoxical Comparisons and Finding Good Approximations 8

3 Computing Multi-Goal Security Strategies (MGSS) **9**
3.1 The case of only one security goal 10
3.2 Assuring Convergence 15
3.3 Numerical Aspects 19
3.4 Remaining Possible Convergence Issues 22
3.5 Exact Solution by Linear Programming 23
3.6 Implementation Hints for Algorithm 1 – Overview 24
3.7 The case of $d > 1$ security goals 25
3.7.1 Prioritization of Security Goals 25
3.7.2 The Full Procedure 26

4 Summary and Outlook **28**

1 Introduction

Having laid the theoretical foundations in part one of this research (see [6]), we now carry on describing the algorithmic aspects and implementation notes for computing risk assurances on concrete games with distributions as payoffs.

We start with a discussion on how to construct distributions from data in a way that is suitable for efficiently deciding $\preceq$-preferences among the empirical distribution estimates. In a nutshell, the $\preceq$-relation as defined in [6] is as follows: assume that R_1, R_2 describe the losses as bounded quantities between 1 and some finite maximum (bounded support), and let them have absolutely continuous measures w.r.t. the Lebesgue or counting measure (assumption 1.3 in [6]).

Definition 1.1 (Preference Relation over Probability Distributions) *Let R_1, R_2 be two bounded random variables ≥ 1, whose distributions are F_1, F_2. Write $m_{R_i}(k)$ for the k-th moment of R_i. We* prefer *F_1* over *F_2, written as*

$$F_1 \preceq F_2 :\iff \exists K \in \mathbb{N} \text{ s.t. } \forall k \geq K : m_{R_1}(k) \leq m_{R_2}(k) \tag{1}$$

Strict preference *of F_1 over F_2 is denoted and defined as*

$$F_1 \prec F_2 :\iff \exists K \in \mathbb{N} \text{ s.t. } \forall k \geq K : m_{R_1}(k) < m_{R_2}(k)$$

Likewise, we define $F_1 \equiv F_2 \iff (F_1 \preceq F_2) \wedge (F_2 \preceq F_1)$.

Observe that $\equiv$ does not mean an identity (in the sense of equality) between F_1 and F_2.

Concrete algorithms are given for estimating continuous distribution models (section 2.1), and on how to compare the following pairs:

- two continuous distributions with finite support (section 2) or infinite support (section 2.4).
- continuous distribution vs. crisp number (section 2.3).

Chapter 3 is devoted to algorithms for computing multi-goal security strategies. It opens with a discussion on how to carry over conventional fictitious play to ${}^*\mathbb{R}$ for one security goal (section 3.1), highlighting several nontrivial pitfalls that must be avoided in a practical implementation. The full algorithm is developed along a sequence of subsections, culminating in the final description for the one-dimensional case in section 3.1. The generalization of the algorithm to multicriteria distributions is derived on these grounds in section 3.7.

The second major aspect of this work is computing security strategies in multi-criteria games. These can be shown to correspond to Nash-equilibria in properly transformed games, however, their computation is somewhat more involved than in the real-valued case. For convenience of the reader, we review the definition of multi-goal security strategies in section 3, after having highlighted the practical and theoretical obstacles in computing (general) Nash-equilibria in the special kind of games that we consider. This discussion is subject of sections 3.2 and 3.3.

2 Comparing Distributions Efficiently

This section is devoted to special cases of distribution models and how to compare them. In many cases, we can avoid computing moment sequences, such as when the distribution can be approximated or has compact support. The latter can be assured in kernel density estimations using the Epanechnikov kernel, which is the first case discussed now.

2.1 Estimating Payoff Distributions from Simulations

In light of assuming bounded supports for all payoff distributions (see the introduction or [8]), it is useful to estimate payoff distributions from data sets in a way that preserves compactness of the support and continuity of the resulting distribution. To this end, we can construct a standard kernel density estimator upon a kernel with compact support, such as the Epanechnikov kernel

$$k(x) := \begin{cases} \frac{3}{4}(1-x^2), & |x| \leq 1 \\ 0, & \text{otherwise,} \end{cases} \tag{2}$$

plotted in figure 1.

Let $N = \{x_1, \ldots, x_n\} \subset \mathbb{R}$ be a sample of n real-valued simulation results, all of which have been harvested under a fixed (i.e., constant) configuration of choices (for example, strategies of all players in the game under consideration).

Nadaraya's theorem then assures uniform convergence of the kernel density estimator

$$\hat{f}(x) = \frac{1}{n \cdot h} \sum_{i=1}^{n} k\left(\frac{x_i - x}{h}\right), \tag{3}$$

towards the unknown payoff distribution density dF/dx, provided that the latter is uniformly continuous, and the bandwidth parameter h is set to $h := c \cdot n^{-\alpha}$ for constants $c > 0$ and $0 < \alpha < \frac{1}{2}$. Practical heuristics (rules of thumb) are available and implemented in various software packages, like `R` [5].

While this choice is convenient for evaluating the $\preceq$-relation, computing equilibria requires a different choice for the kernel function, which will be the Gaussian kernel (or any kernel with infinite support). We go into details later in section 3.2 and afterwards.

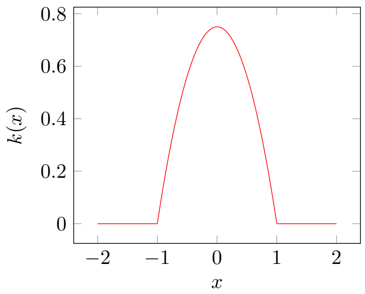

Figure 1: Plot of the Epanechnikov kernel

2.2 Comparing Kernel Density Models

Given two kernel density estimates by using their hyperreal representatives is easy, and made precise in the following procedure (cf. [7]):

Let $\hat{f}_1, \hat{f}_2$ be two kernel density estimates (3), constructed from sample sets of sizes n_1, n_2, with respective bandwidths h_1, h_2. Furthermore, let us assume that the kernel has compact support, which is – for example – automatically satisfied for the Epanechnikov kernel. Let the samples be in ascending order (that can be arbitrary for ties), i.e., the densities $\hat{f}_1, \hat{f}_2$ are constructed from the values $x_1 \leq x_2 \leq \cdots \leq x_{n_1}$ and $y_1 \leq y_2 \leq \cdots \leq y_{n_2}$. To decide which one of $\hat{f}_1, \hat{f}_2$ is preferred over the other, it suffices to look at the largest value in each sample. Here, we distinguish the following cases:

- If $x_{n_1} + h_1 < y_{n_2} + h_2$, then $\hat{f}_1 \prec \hat{f}_2$.
- If $y_{n_2} + h_2 < x_{n_1} + h_1$, then $\hat{f}_2 \prec \hat{f}_1$.
- Otherwise, we must have $x_{n_1} + h_1 = y_{n_2} + h_2$, in which case we go on comparing as follows:
  - If $x_{n_1} < y_{n_2}$, then $\hat{f}_1 \prec \hat{f}_2$
  - If $y_{n_2} < x_{n_1}$, then $\hat{f}_2 \prec \hat{f}_1$
  - Otherwise, we must have $x_{n_1} = y_{n_2}$, and therefore also $h_1 = h_2$. In that case, we discard the pair x_{n_1}, y_{n_2} and repeat the comparison procedure on the next samples x_{n_1-1}, y_{n_2-1} in decreasing order on both lists.

The effort for sorting then makes the above procedure decide the $\prec$-relation with complexity $O(n \log n)$, where $n = \max\{n_1, n_2\}$.

The correctness of this method is immediately evident upon the fact that any two distinct points $z_i \neq z_j$ in a sequence of samples contribute to the (respective) density using bandwidth h on a range $[z-h, z+h]$, where $z \in \{z_i, z_j\}$. A subtle issue arises in the case of categorial data (say, if the outcome is rated as "low", "medium", "high"), for which identical samples may accumulate at identical positions. In this case, the density with less samples in the higher range will be preferred. In the above process, this will cause identical samples to be removed until either…

- …exactly one of the distributions has no further samples at position z, which makes this preferred (since the other distribution assigns a nonzero likelihood to larger damage possibilities), or
- …both distributions have an equal amount of probability mass on z, in which case the respective density functions are identical and the difference between the densities is zero.

In any case, this means that we can ultimately assume $z_i \neq z_j$, so that the respective intervals are not congruent. W.l.o.g., assume $z_i < z_j$, then the density is strictly positive on the range $[z_j - h, z_j + h] \setminus [z_i - h, z_i + h]$, whereas it vanishes outside the interval. Now, apply this reasoning to two densities, having their shapes on the right end of their

support being defined by the kernels centered around the maximum value in either data sample. Depending on the bandwidth in each estimator and the location of the interval, it remains to determine which density reaches farther out and remains positive when the other density vanishes. The arguments in the proof of [6, lemma 2.4] exhibit that the distribution whose support strictly overlaps the other that will have its moments grow faster than the distribution that it is compared to. Hence, the preference relation $\prec$ can be decided upon checking which density estimate has the longer tail.

To improve flexibility in this regard, let us look at mixture distribution models, which are distribution functions whose density is a mix of several (simpler) density functions, i.e.,

$$f_{\text{mix}}(x) = \sum_{i=1}^{n} \omega_i f_i(x), \quad \text{where} \quad \sum_{i=1}^{n} \omega_i = 1.$$

From the mix of densities, the mixed distribution is instantly discovered to be

$$F_{\text{mix}}(x) = \sum_{i=1}^{n} \omega_i F_i(x)$$

Likewise, the moments of $X \sim F_{\text{mix}}$ satisfy

$$\mathrm{E}\left(X^k\right) = \int_0^\infty x^k f_{\text{mix}}(x)dx = \sum_{i=1}^{n} \omega_i \mathrm{E}\left(X_i^k\right), \quad \text{where} \quad X_i \sim F_i \tag{4}$$

From the last identity, we instantly obtain that for any two distributions $F_1 \preceq F_2$ and $G_1 \preceq G_2$, preference holds for the mix in the same way, i.e. (by induction), if $F_i \preceq G_i$ for $i = 1, 2, \ldots, n$, then $F_{\text{mix}} \preceq G_{\text{mix}}$, with the mixtures defined as above.

Mixture distribution models are particularly handy as they can be shown to approximate *any* distribution up to arbitrary precision.

Having mixtures as models, it is interesting to see what happens if the mixture is such that components compare alternatingly. In other words, let $F_1 = \omega_1 G_1 + \omega_2 G_2$ and $F_2 = \omega_1' H_1 + \omega_2' H_2$, where $G_1 \preceq H_1$ but $G_2 \succeq H_2$.

Here, we can take advantage of the hyperreal representation of distributions which allows us to do arithmetics "as usual", whereas it must strictly be emphasized that the result of any such arithmetics no longer represents a valid distribution (nor is the arithmetic done here in any way statistically meaningful; it is a mere vehicle towards the sought conclusion).

More specifically, recall that any of the above distributions is represented by a sequence of moments, and the comparison is based on which sequence grows faster than the other.

This means that by virtue of (4) and by letting the sequences $(g_{1,n})_{n\in\mathbb{N}}$, $(g_{2,n})_{n\in\mathbb{N}}$, $(h_{1,n})_{n\in\mathbb{N}}$ and $(h_{2,n})_{n\in\mathbb{N}}$ represent the distributions G_1, G_2, H_1 and H_2, we end up looking at the limit

$$a = \lim_{n\to\infty} \Big[\underbrace{(\omega_1 g_{1,n} + \omega_2 g_{2,n})}_{\text{represents } F_1} - \underbrace{(\omega_1' h_{1,n} + \omega_2' h_{2,n})}_{\text{represents } F_2} \Big]$$

If $a \leq 0$ then $F_1 \preceq F_2$, with strict preference if $a < 0$. Otherwise, we have $F_1 \succeq F_2$, likewise with strict preference. More detailed criteria call for additional hypotheses, such as the existence of closed-form expressions for the involved moments. Many special cases, however, are easy to decide, such as mixes of distributions with compact support. In the case of finite mixtures, F_{mix} is supported on $\bigcup_{i=1}^{n} \text{supp}(F_i)$, and has itself compact support. Then, all the previously given criteria apply. Hence, the above limit expression comes into play when comparing distributions with infinite support, and calls for a full-fledged analysis only if approximations (by truncating the distributions) are not reasonable.

2.3 Comparing Deterministic to Random Variables

Let $a \in \mathbb{R}$ be a deterministic outcome of an action, and let $Y \sim F$ be a random variable with non-degenerate distribution supported on $\Omega = [0, b]$ whose density is f. We know that a yields a moment sequence $(a^k)_{k\in\mathbb{N}}$. A comparison to Y is easy in every of the three possible cases:

1. If $a < b$, then $a \preceq Y$: to see this, choose $\varepsilon < (b-a)/3$ so that f is strictly positive on a compact set $[b-\varepsilon, b-2\varepsilon]$ (note that such a set must exist as f is continuous and the support ranges until b). We can lower-bound the k-th moment of Y as

$$\begin{aligned}\int_0^b y^k f(y)dy &\geq \left(\inf_{[b-2\varepsilon, b-\varepsilon]} f\right) \cdot \int_{b-2\varepsilon}^{b-\varepsilon} y^k dy \\ &= \frac{1}{k+1}\left[(b-\varepsilon)^{k+1} - (b-2\varepsilon)^{k+1}\right].\end{aligned}$$

   Note that the infimum is positive as f is strictly positive on the compact set $[b-2\varepsilon, b-\varepsilon]$. The lower bound is essentially an exponential function to a base larger than a, since $b - 2\varepsilon > a$, and thus (ultimately) grows faster than a^k.

2. If $a > b$, then $Y \preceq a$, since Y – in any possible realization – leads to strictly less damage than a. The formal argument is now based on an upper bound to the moments, which can be derived as follows:

$$\int_0^b y^k f(y)dy \leq (\sup_{[0,b]} f) \cdot \int_0^b y^k dy = (\sup_{[0,b]} f)\frac{1}{k+1}b^{k+1}.$$

   It is easy to see that for $k \to \infty$, this function grows slower than a^k as $a > b$, which leads to the comparison result.

3. If $a = b$, then we apply the mean-value theorem to the integral occurring in $\mathrm{E}(Y^k) = \int_0^a y^k f(y)dy$ to obtain an $\xi \in [0, a]$ for which

$$\mathrm{E}\left(Y^k\right) = \xi^k \underbrace{\int_0^a f(y)dy}_{=1} = \xi^k \leq a^k$$

for all k. Hence, $Y \preceq a$ in that case. An intuitive explanation is obtained from the fact that Y may assign positive likelihood to events with less damage as a, whereas a deterministic outcome is always larger or equal to anything that Y can deliver.

2.4 Distributions with Infinite Support

This case is difficult in general, but simple special cases may occur, for example, if we compare a distribution with compact support to one with infinite support (such as extreme value distributions or ones with long or fat tails). Then, the compactly supported distribution is always preferred, by the same argument as used above (and in the proof of the invariance of $\preceq$ w.r.t. the ultrafilter used to construct $^*\mathbb{R}$; see [6, lemma 2.4]).

The unfortunate occasions are those where:

- both distributions have infinite support, and
- neither [6, Lemma 2.7] nor any direct criteria (such as eq. (13) in [6]) apply, and
- an approximation cannot be done (for whatever reason).

Then we have to work out the moment sequences explicitly. This situation is indeed problematic, as without assuming bounded supports, we can neither guarantee existence nor divergence of the two moment sequences.

Appropriate examples illustrating this problem can easily be constructed by defining distributions with alternating moments from the representation by the Taylor-series expansion of the characteristic function (see [6] for an example).

As was explained in [6], however, explained that extreme value distributions strictly compare to one other depending depending on their parameterizations, by applying lemma the criteria derived in [6]. Mixes of such distributions can perhaps replace an otherwise unhandy model.

2.5 On Paradoxical Comparisons and Finding Good Approximations

In part one of this report, finite approximations to distributions with infinite support were proposed to fit these into our preference relation. It turns out, however, that even distributions with finite support may lead to paradoxical and unexpected results in terms of $\preceq$-preference. That is, we would in any case prefer the distribution with smaller support, but this is not necessarily the one giving us less damage in most of the cases.

To illustrate the problem, consider the two distributions plotted in figure 2 (where the densities have been scaled only to properly visualize the issue). Observe that F_2 assigns all its mass around larger damage, while F_1 generally gives much lower damages except for rare cases that exceed the events that can occur under F_2. These rare cases, however, extend beyond the support of distribution F_1, which based on the characterization by a sequence of would clearly let us prefer F_2 over F_1 (Figure 2a). Indeed, it is easy to see that such a result is not what we would expect or want in practice.

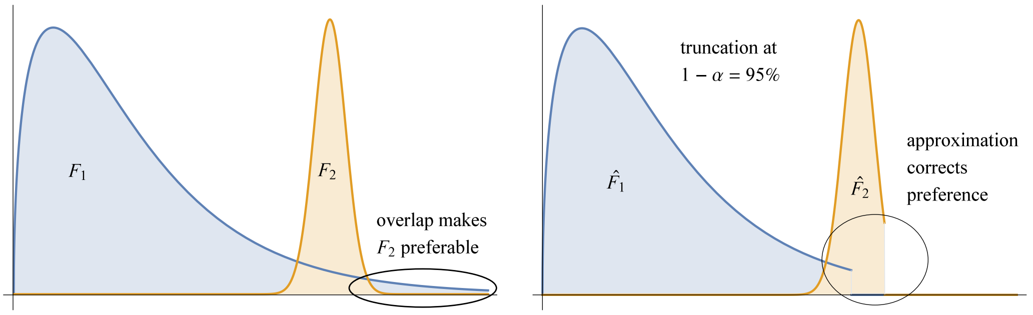

(a) Distributions $F_2 \preceq F_1$ (b) Truncated distributions $\hat{F}_1 \preceq \hat{F}_2$

Figure 2: Correcting Paradoxical Comparisons by Quantile-Based Approximations [7]

Truncating the distributions properly can, however, easily fix the issue. To this end, let $\alpha > 0$ be a *chosen and fixed* threshold and cut off each distribution as soon as it exceeds $1-\alpha$ of its mass. Precisely, we could thus cut off a distribution at its $(1-\alpha)$-quantile, denoted as $q_{1-\alpha} = F^{-1}(1-\alpha)$ (where F^{-1} is the inverse of the distribution function; called the *quantile function*). Now, reconsider the previous situation with F_1 and F_2, now being truncated properly (Figure 2b). Since both distributions' supports extend only to the point when they have assigned 95% of their mass, it is instantly revealed that F_2 does so much earlier than F_1, hence making F_2 clearly the preferred option here. The price of this fix is the *acceptance* of events that are less likely than α under F_2, which can – in a fraction of α among all possible cases – give more damage than F_1 could ever do.

In other words, common approximations based on a *risk acceptance threshold* α, as described above, can avoid paradoxical preferences. The assertion of theorem 2.14 in [6] would then fail in claiming that extreme events are less likely under the preferred distribution. The assertion, however, remains valid in a fraction of $(1-\alpha)\%$ of cases, if we cut off the distributions.

3 Computing Multi-Goal Security Strategies (MGSS)

Roughly speaking, the basic algorithm to compute MGSS in the sense of [6, Def.4.1] (generalizing precursor work in [9]). For the definition, let $\mathfrak{F} \subseteq^* \mathbb{R}$ be a subset of the hyperreals $^*\mathbb{R}$ that represent probability distributions that are supported within a compact subset of $[1, a]$ for some real number a. For two n-dimensional vectors $\boldsymbol{x} = (x_1, \ldots, x_n), \boldsymbol{y} = (y_1, \ldots, y_n)$, we write $\boldsymbol{x} \preceq_1 \boldsymbol{y}$, if an index i exists for which $x_i \preceq y_i$ (irrespectively of what the other entries do).

Definition 3.1 (Multi-Goal Security Strategy with Assurance)
A strategy $\boldsymbol{p}^* \in S_1$ in a two-person multi-criteria game with mixed strategy spaces S_1, S_2. Let player 1 be a maximizer*, and have a continuous payoff function $\boldsymbol{u}_1 : S_1 \times S_2 \to \mathfrak{F}^d$. A* Multi-Goal Security Strategy with Assurance *(MGSS) with* assurance *is a pair $(\boldsymbol{v}, \boldsymbol{x}^*)$,*

where $\boldsymbol{v} = (V_1, \dots, V_d) \in \mathfrak{F}^d$ *is vector of distributions and* $\boldsymbol{x}^* \in S_1$*, if two criteria are met:*

Axiom 1: Assurance *The values in* $\boldsymbol{v}$ *are the component-wise guaranteed payoff for player 1, i.e. for all components* i*, we have*

$$V_i \preceq u_1^{(i)}(\boldsymbol{p}^*, \boldsymbol{q}) \qquad \forall \boldsymbol{q} \in S_2, \tag{5}$$

with $\equiv$*-equality being achieved by at least one choice* $\boldsymbol{q}_i \in S_2$*.*

Axiom 2: Efficiency *At least one assurance becomes void if player 1 deviates from* $\boldsymbol{p}^*$ *by playing* $\boldsymbol{p} \neq \boldsymbol{p}^*$*. In that case, some* $\boldsymbol{q}_{\boldsymbol{p}} \in S_2$ *exists (that depends on* $\boldsymbol{p}$*) such that*

$$\boldsymbol{u}_1(\boldsymbol{p}, \boldsymbol{q}_{\boldsymbol{p}}) \preceq_1 \boldsymbol{v}. \tag{6}$$

To practically compute MGSS, we actually "simulate" a gameplay in which both players (honest defender and the full set of adversaries, each of which corresponds to another security goal), record each others actions and optimize their choices w.r.t. the empirical distributions[1].

3.1 The case of only one security goal

We start with the simpler case in which the MGSS is one-dimensional. This case boils down to an application of regular fictitious play to a "standard" zero-sum game. The general case of $d > 1$ goals can easily be reduced to the one-dimensional case, as we will show in section 3.7.

For the current matter, it suffices to decide upon one's best action, given that the others choose their actions with known probabilities. This is nothing else than a mix of distributions that we need to compare to our own strategy (details of how this can be done have been discussed before).

To exactly specify a suitable fictitious play (FP) algorithm, we will step-by-step transfer a MATLAB implementation of a regular FP algorithm as described in [13] to our modified setting. Towards proving correctness of the algorithm in our setting, the hyperreal numbers will turn out useful.

Listing 1: Fictitious play in MATLAB for a zero-sum game with payoff matrix $\boldsymbol{A} \in \mathbb{R}^{n \times m}$ and with a maximizing player 1/minimizing player 2

```
% standard fictitious play in an two person
% zero sum (mxn)-matrix game with
% payoff matrix A. The computation delivers
% an approximate Nash equilibrium
% over iterationCount steps (must be set in advance)
[m n] = size(A);
x = zeros(1, m);
y = zeros(1, n);
```

[1] in the literature, this is called a *belief sequence*.

```
9  [vmin, r] = max(min(A'));
10 [vmax, c] = min(max(A));
11 U = A(:, r);
12 V = zeros(1, n);
13 y(c) = y(c) + 1;
14 for i = 1:iterationCount-1
15    [bestU, r] = max(U); % best response to player 2's actions
16    x(r) = x(r) + 1;            % update player 1's behavior
17    V = V + A(r, :);            % record player 1's payoff
18    vmax = min(bestU / i, vmax); % update the upper bound to v
19    [bestV, c] = min(V); % best response to player 1's actions
20    y(c) = y(c) + 1;            % update player 2's behavior
21    U = U + A(:, c);            % record player 2's payoff
22    vmin = max(bestV / i, vmin); % update the lower bound to v
23 end
24 x = x / sum(x);    % absolute frequencies -> probabilities
25 y = y / sum(y);
26 v = x*A*y';        % average payoff (estimated)
```

Lines $6, \ldots, 13$ in the algorithm cover matters of initialization, where we determine the size of the payoff structure ($n \times m$ being assumed here), and initialize the solution vectors $\boldsymbol{x}$ and $\boldsymbol{y}$ to all zero. Upon termination of the algorithm, $\boldsymbol{x}$ and $\boldsymbol{y}$ will approximate the MGSS $\boldsymbol{p}^*$ and an optimal adversarial mixed strategy $\boldsymbol{q}^*$.

Remark 3.2 *Note that our problem is to minimize player 1's loss, while player 2 (adversary) attempts to maximize the damage. This is essentially the opposite of what is implemented in listing 1, so that our first modification is to swap all calls to* min *and* max *in the entire algorithm (lines* $9 \ldots 23$*). The rest of the algorithm remains unchanged. Nevertheless, we will continue the explanation letting player 1 be a maximizer here, without loss of generality.*

Now for the for-loop (lines $14 \ldots 23$): during the initialization phase, the FP algorithm chooses an arbitrary initial pure strategy $1 \leq i_1 \leq n$ for player 1 (done in lines 9 and 10 of listing 1). From then on, the players alternatingly choose their next pure strategies as a best reply assuming that the other player selects at random from her/his previous choices. That is, let the history of player 1's (row-player) moves be $r_1, \ldots, r_k$ over the past k iterations of FP, then player 2 (column-player) chooses his strategy c_k so as to minimize the average payoff

$$c_k = \underset{j \in PS_2}{\operatorname{argmin}} \frac{1}{k} \sum_{\ell=1}^{k} a_{i_\ell, j}, \tag{7}$$

when the payoff matrix is $\boldsymbol{A} = (a_{i,j})_{i,j=1}^{n,m}$. Let this choice be c_{k+1}, then likewise, player

1 would in the next round choose r_{k+1} to maximize the average outcome

$$r_{k+1} = \operatorname*{argmax}_{i \in PS_1} \frac{1}{k} \sum_{\ell=1}^{k} a_{i,j_\ell}. \tag{8}$$

These alternating choices are made in lines 15…17 and 19…21 in the MATLAB code. Lines 16 and 20 simply count so-far played moves in a vector, which, after normalization, will be the sought approximation to the Nash-equilibrium $(\boldsymbol{p}^*, \boldsymbol{q}^*)$.

In light of the fact that our games pay the players in terms of entire probability distributions rather than numeric values, line 17 and line 21 deserve a closer look: Indeed, the division by the iteration count in lines 18 and 22 is nothing else than the factor $1/k$ appearing in (7) and (8), so that both expressions

$$\frac{1}{k} \sum_{\ell=1}^{k} F_{i_\ell,j}, \quad \text{and} \quad \frac{1}{k} \sum_{\ell=1}^{k} F_{i,j_\ell} \tag{9}$$

yield valid distribution functions again, when we use our payoff distribution matrix $\boldsymbol{A} = (F_{ij})$ instead of real values. As was previously noticed in equation (4) already, the corresponding hyperreal representation of a weighted sum $\frac{1}{k}\sum_{\ell=1}^{k} F_{i_\ell,j}$ is identical to the correspondingly weighted sum of hyperreal representations of each term (independently of the ultrafilter upon which the hyperreal space $^*\mathbb{R}$ was constructed). In other words, working with distributions in (7) and (8) amounts to leaving the algorithm exactly unchanged, except for letting it operate on hyperreal numbers instead of real numbers to represent the payoffs. By the transfer principle [10], convergence of the algorithm identically holds in the space of hyperreals as it does in the real numbers (since the respective statements are all in first-order logic). It follows that the resulting algorithm pointwise adds distribution or density functions in lines 17 and 21, and it divides by the iteration count as it would divide a standard real number (only, it is a distribution function in our case). This is permitted, since the pointwise scaling of a distribution function by a real value amounts to scaling the respective hyperreal number (moment sequence) element-wise by the same factor.

Convergence of the algorithm then implies that the approximations $\boldsymbol{x}$ and $\boldsymbol{y}$ will eventually approximate an equilibrium in the $\preceq$-sense (asymptotically). Since the outcome of the algorithm over the reals is an approximation to a Nash-equilibrium $(\boldsymbol{x}^*, \boldsymbol{y}^*)$ w.r.t. the payoff being $\boldsymbol{x}^T \boldsymbol{A} \boldsymbol{y}$, the outcome of our algorithm is a hyperreal pair $(\boldsymbol{x}^*, \boldsymbol{y}^*)$ that forms an equilibrium for the payoff function $\boldsymbol{x}^T \boldsymbol{A} \boldsymbol{y}$, which, however, equals the sought overall distribution of the damage R,

$$\Pr(R \leq r) = (F(\boldsymbol{p}, \boldsymbol{q}))(r) = \sum_{i,j} F_{ij}(r) \cdot p_i \cdot q_j, \tag{10}$$

with independent choices by both players.

It is important to notice the difference of this outcome to that of standard game-theory, where a "repeated game" means something essentially different than here:

- In standard game-theory, the players would choose their payoffs to maximize their long-run average over *infinitely many repetitions* of the game.

- In our setting, as implied by (10), the Nash-equilibrium optimizes the game's overall payoff distribution *for a single round.* That is, this consideration is a single-shot, as opposed to conventional games, where we benefit only in the long-run from playing the equilibrium.

The whole trick is equation (10) resembling a long-run average payoff for a conventional game, when the strategy choices are made independently. Since our distributions boil down to mere hyperreal *numbers* in the space $\mathfrak{F} \subset \mathbb{R}^{\infty}/\mathcal{U}$, convergence there is inherited from the convergence of regular FP over the reals. More importantly, observe that the FP algorithm merely adds and scales distributions (by a real valued factor), which is doable without any explicit ultrafilter $\mathcal{U}$. Thus, regular FP can be applied *seemingly without change* to our setting, except for the min- and max-functions being computed w.r.t. $\preceq$-relation (and the criteria as specified above). Alas, this impression will turn out to be practically flawed (example 3.5).

Let us now turn to the full pseudocode-specification of fictitious play in our modified setting, which is algorithm 1. Note that this algorithm is also adapted towards letting player 1 (the defender) be a minimizer now.

Convergence of algorithm 1 is inherited from the known convergence results in the standard setting of fictitious play, as shown in listing 1. Precisely, we have the following result, originally proven by J.Robinson [11]. Our version here is an adapted compilation of proposition 2.2 and proposition 2.3 in [2, chapter 2]:

Theorem 3.3 *Under fictitious play in* $^*\mathbb{R}$*, the empirical distributions converge (along an unboundedly growing sequence of hyperreal integers), if the game is zero-sum.*

Remark 3.4 *The zero-sum assumption in theorem 3.3 needs some elaboration here: let* $\boldsymbol{A}$ *be the original payoff structure with all distribution-valued entries. Let* $\underline{\boldsymbol{A}}$ *be its hyperreal representation (by replacing each distribution by its representative moment sequence). Then, a zero-sum game – as considered in theorem 3.3 – would assign the payoffs* $(\underline{\boldsymbol{A}}, -\underline{\boldsymbol{A}})$*, in which player 2's structure no longer corresponds to a valid matrix of probability distributions. However, FP merely means the players choosing their moves as a* best response *to the so-far recorded history of strategies. For player 2,* $\preceq$*-maximizing his payoff on* $-\underline{\boldsymbol{A}}$ *is the same as* $\preceq$*-minimizing the payoff on* $\underline{\boldsymbol{A}}$ *by the properties of the ordering on the hyperreals. Thus, his choices are indeed based on a valid probability distribution matrix.*

We stress that theorem 3.3 talks about the convergence of *empirical distributions*, rather than the convergence of the game's *value* only (which is the somewhat weaker statement found in [11]). The importance of this insight for us lies in the convergence criterion that it delivers:

> Convergence criterion for algorithm 1, line 20: let $\boldsymbol{x}_k$ denote the empirical absolute frequencies of strategy choices, as recorded by algorithm 1 in line

Algorithm 1 Fictitious Play (with $\preceq$-minimizing first player)

Require: an $(n \times m)$-matrix $\boldsymbol{A}$ of payoff distributions $\boldsymbol{A} = (F_{ij})$

Ensure: an approximation $(\boldsymbol{x}, \boldsymbol{y})$ of an equilibrium pair $(\boldsymbol{p}^*, \boldsymbol{q}^*)$ and a two distributions v_{low}, v_{up} so that $v_{low} \preceq F(\boldsymbol{p}^*, \boldsymbol{q}^*) \preceq v_{up}$. Here, $F(\boldsymbol{p}^*, q^*)(r) = \Pr(R \leq r) = \sum_{i,j} F_{ij}(r) \cdot p_i^* q_j^*$.

initialize $\boldsymbol{x} \leftarrow \boldsymbol{0} \in \mathbb{R}^n$, and $\boldsymbol{y} \leftarrow \boldsymbol{0} \in \mathbb{R}^m$
$v_{low} \leftarrow$ the $\preceq$-minimum over all column-maxima
$r \leftarrow$the row index giving v_{low}
$v_{up} \leftarrow$ the $\preceq$-maximum over all row-minima
$c \leftarrow$ the column index giving v_{up}
$\boldsymbol{u} \leftarrow (F_{1,c}, \ldots, F_{n,c})$
$y_c \leftarrow y_c + 1$ ▷ $\boldsymbol{y} = (y_1, \ldots, y_m)$
$\boldsymbol{v} \leftarrow \boldsymbol{0}$ ▷ initialize $\boldsymbol{v}$ with m functions that are zero everywhere
for $k = 1, 2, \ldots$ **do**
  $u^* \leftarrow$ the $\preceq$-minimum of $\boldsymbol{u}$
  $r \leftarrow$ the index of u^* in $\boldsymbol{u}$
  $v_{up} \leftarrow$ the $\preceq$-maximum of $\{u^*/k, v_{up}\}$ ▷ pointwise scaling of the distribution u^*
  $\boldsymbol{v} \leftarrow \boldsymbol{v} + (F_{r,1}, \ldots, F_{r,m})$ ▷ pointwise addition of functions
  $x_r \leftarrow x_r + 1$ ▷ $\boldsymbol{x} = (x_1, \ldots, x_n)$
  $v_* \leftarrow$ the $\preceq$-maximum of $\boldsymbol{v}$
  $c \leftarrow$ the index of v_* in $\boldsymbol{v}$
  $v_{low} \leftarrow$ the $\preceq$-minimum of $\{v_*/k, v_{low}\}$ ▷ pointwise scaling of the distribution v_*
  $\boldsymbol{u} \leftarrow \boldsymbol{u} + (F_{1,c}, \ldots, F_{n,c})$ ▷ pointwise addition of functions
  $y_c \leftarrow y_c + 1$ ▷ $\boldsymbol{y} = (y_1, \ldots, y_m)$
  exit the loop upon convergence ▷ concrete condition given below
end for
Normalize $\boldsymbol{x}, \boldsymbol{y}$ to unit total sum ▷ turn $\boldsymbol{x}, \boldsymbol{y}$ into probability distributions.
return $\boldsymbol{p}^* \leftarrow \boldsymbol{x}, \boldsymbol{q}^* \leftarrow \boldsymbol{y}$, and $F(\boldsymbol{p}^*, \boldsymbol{q}^*) \leftarrow \sum_{i,j} F_{ij}(r) \cdot x_i \cdot y_j$ ▷ $\approx (\boldsymbol{p}^*)^T \boldsymbol{A} \boldsymbol{q}^*$

14. Fix any $\varepsilon > 0$ and some vector-norm $\|\cdot\|$ on $\mathbb{R}^n$, and terminate the algorithm as soon as $\frac{1}{k}\|\boldsymbol{x}_{k+1} - \boldsymbol{x}_k\| < \varepsilon$.

Alternative convergence criteria, say on the difference $|v_{up} - v_{low}|$ are also found in the literature, but are not applicable to our setting here: to see why, recall that both, v_{up} and v_{low} are probability distributions, and convergence $|v_{up} - v_{low}| \to 0$ holds in a hyperreal sense (by an application of the transfer principle, or more generally, Łoś's theorem [1]). Precisely, the statement would be the following: for every hyperreal $\varepsilon > 0$ there is a number K such that $\left|v_{up}^{(k)} - v_{low}^{(k)}\right| < \varepsilon$ for all hyperreal $k \geq K$, where $v_{up}^{(k)}, v_{low}^{(k)}$ denote the values in the k-th iteration of the algorithm.

An inspection of Jane Robinson's original convergence proof [11] shows that despite all statements transfer to the hyperreal setting (consistently with Łoś's theorem), convergence kicks in beyond an integer limit that depends on the *largest* entry in the matrix. More specifically, given $\varepsilon > 0$ and letting $t \in \mathbb{N}$ be an iteration counter, Robinson's proof (cf. lemma 4 in [11]) establishes at some point that $\max \boldsymbol{v}(t) - \min \boldsymbol{u}(t) < \varepsilon$ conditional on

$$t \geq 8 \cdot a \cdot t^*/\varepsilon, \tag{11}$$

based on an induction argument that delivers t^* through the induction hypothesis. The crucial point here is the number a, which is the absolute value of the largest element in the payoff-matrix. Now, even if t were (at some point in the induction) a finite integer, (11) asserts a hyperreal lower bound for t. By our construction, a represents a probability distribution with a diverging moment-sequence (that is a) and thus an infinite hyperreal number. Consequently, the number of iterations until convergence is a hyperreal and infinite integer. In other words, convergence of algorithm (1), when implemented in plain form over the hyperreals, cannot converge for a standard integer for-loop (in line 9).

3.2 Assuring Convergence

The problem can be made visible even on a less abstract level, by considering an intermediate step in the algorithm that has established mixtures u and v as multimodal distributions. Consider the intermediate values computed by algorithm 1, using the following concrete example for illustration.

Example 3.5 (Convergence failure of plain FP implementations) *We construct a 2×2-game with payoffs being Epanechnikov density functions centered around the values*

$$\boldsymbol{A} = \begin{pmatrix} 2 & 5 \\ 3 & 1 \end{pmatrix}$$

The payoff structure in our game is thus a 2×2-matrix of functions,

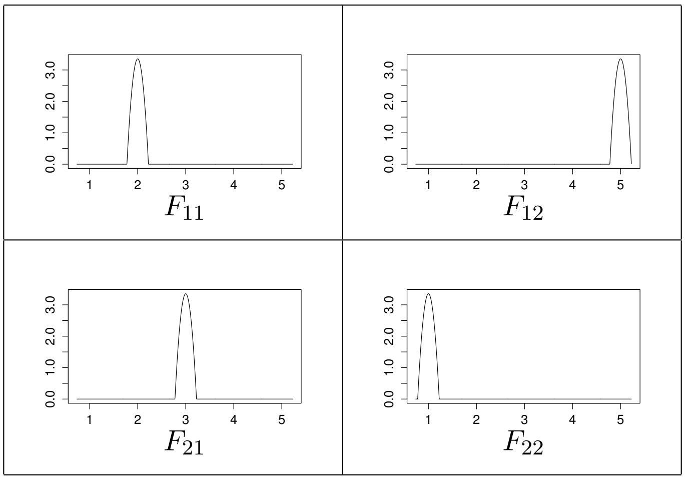

It is easy to compute the Nash-equilibrium for the exact matrix $\boldsymbol{A}$ *as* $v(\boldsymbol{A}) = 2.6$, *returned by the mixed equilibrium strategies* $\boldsymbol{p}^* = (0.4, 0.6)$ *and* $\boldsymbol{q}^* = (0.8, 0.2)$ *for both players. Since our setting shall merely capture our uncertainty about the payoffs, we would thus naturally expect a somewhat similar result when working on the payoff* distributions. *Unfortunately, however, algorithm 1 will not produce this expected answer, if it is implemented without care.*

After a few iterations, the algorithm gets stuck in always choosing the first row for player 1, since v_{up} *is always* $\preceq$*-preferable over* u^*/k*, which is immediately obvious from plotting the two distributions:*

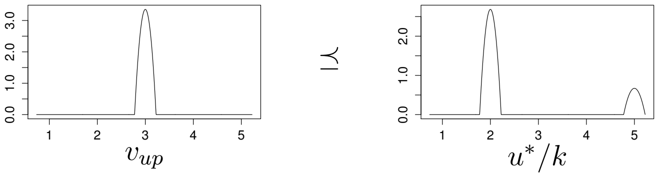

Observe that choosing the upper row in the payoff structure adds probability mass to lower damages, but leaves the tail of the distribution unchanged. Thus, although the overall damage accumulates, this effect is not noticeable by the $\preceq$*-relation. Consequently, the algorithm will counterintuitively come to the conclusion that* $\boldsymbol{p} = (0, 1)$ *is a pure equilibrium, which is definitely not meaningful.*

Let us now make the intuition developed in example 3.5 more rigorous to find the solution for the problem.

Recall that the bounds are updated (lines 12 and 17 in algorithm 1) when u^*/k or v_*/k are preferable over the current bounds. Now, given choices of actions whose payoff distribution has vanishing support at the right end of the relevant interval (choices u^* and v_*), the pointwise scale and accumulation of distributions will make $1/k \cdot u^*$ always larger than v_{up}, simply because v_{up} assigns no mass to regions where u^* has a strictly positive density. This strict positivity is clearly retained upon any scaling by $1/k$, no

matter how large k is. Thus, the algorithm gets stuck with the same action being chosen once and forever, since adding $\preceq$-smaller payoff distributions to the long-run average u^*/k will never make this payoff less than v_{up}, and thus never cause an update to this value. The analogous effect prevents updates to v_{low}, and the algorithm will end up with pure equilibria and thus never converge to a practically meaningful result.

A closer look at the example, however, also reveals the way out of the dilemma: the algorithm hangs because the cumulative sum adds up probability densities pointwise and the comparison is done at the tails of the distribution only. Thus, any change outside the tail region is not noticed when the $\preceq$-relation is evaluated, thus never triggering any updates to v_{up} and v_{low}. Obviously, the effect disappears if all distributions in the payoff structure have full support over the interval of interest, so that any change to the cumulative payoff is "noticeable everywhere", especially at the tail region of the distribution.

This is easily accomplished by replacing the Epanechnikov-kernels by Gaussian kernels in the estimator (3), and truncating the kernel density estimates $\hat{f}$ at a fixed (chosen) point; as recommended already in section 2.5.

To establish the correctness of this fix, we must assure convergence of fictitious play under these new payoff structures. To this end, we will resort to the original version of fictitious play being done over $\mathbb{R}$. In fact, we will construct a real-valued approximation of the hyperreal representation of the payoff structure, so that we can work with standard FP again. The transition from the hyperreal representation back to a real-valued representation is approximative in the sense that we will represent a hyperreal value/distribution F by a real value $y(F)$, to be defined later, in an $\preceq$-order-preserving fashion.

Let $\hat{f}$ be a Gaussian kernel density estimator for a payoff distribution F (more precisely, its density function), which is strictly positive on the entire interval $\Omega = [0, a]$. Since all payoff densities are strictly positive at a, we first observe that for any two densities f_1, f_2, we have

$$\hat{f}_1(a) < \hat{f}_2(a) \text{ implies } \hat{f}_1 \preceq \hat{f}_2.$$

Otherwise, if $f_1(a) = f_2(a)$ (the $>$-case is analogous as before), then the first order derivative may tip the scale in the sense that under equality of the densities at $x = a$,

$$\hat{f}_1'(a) > \hat{f}_2'(a) \text{ implies } \hat{f}_1 \preceq \hat{f}_2.$$

Upon equality of values and slopes, the second order derivative makes the decision, since in that case,

$$\hat{f}_1''(a) < \hat{f}_2''(a) \text{ implies } \hat{f}_1 \preceq \hat{f}_2,$$

and so on. All these three concrete cases are most easily visualized graphically (see Figure 3 for the first two cases), and our next step is making this so far intuitive criterion rigorous. For that matter, recall that the *lexicographical order* on two (infinite) sequences $\boldsymbol{a} = (a_n)_{n\in\mathbb{N}}$ and $\boldsymbol{b} = (b_n)_{n\in\mathbb{N}}$. This ordering is denoted as $<_{lex}$ and calls $\boldsymbol{a} <_{lex} \boldsymbol{b}$ if $a_1 < b_1$. If $a_1 = b_1$, then $\boldsymbol{a} <_{lex} \boldsymbol{b}$ if $a_2 < b_2$, and so on. For strings (whether finite or not), the lexicographical order is simply the alphabetical order. The lexicographical less-or-equal $\leq_{lex}$ order is defined in the obvious way.

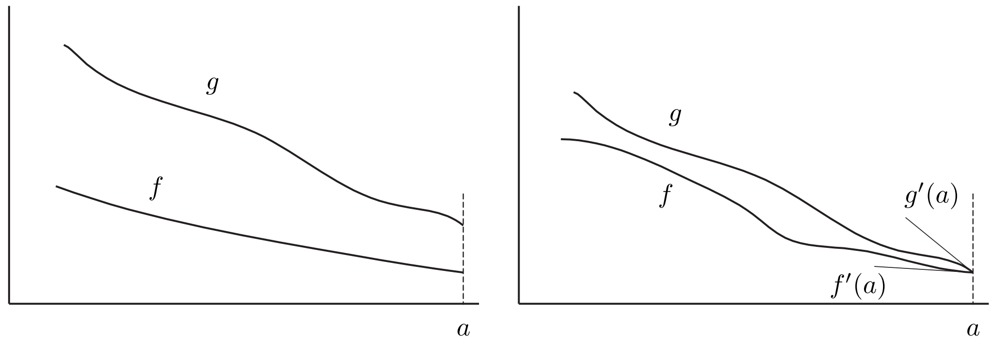

(a) $f(a) < g(a) \Rightarrow f \preceq g$ (b) $(f(a) = g(a) \wedge -f'(a) < -g'(a)) \Rightarrow f \preceq g$

Figure 3: Comparing tails using function values and derivatives

Lemma 3.6 *Let $f, g \in C^\infty([0,a])$ for a real value $a > 0$ be probability density functions. If*

$$((-1)^k \cdot f^{(k)}(a))_{k\in\mathbb{N}} <_{lex} ((-1)^k \cdot g^{(k)}(a))_{k\in\mathbb{N}},$$

then $f \preceq g$.

Thus, it suffices to lexicographically compare the sequence of derivatives with alternating sign-change to decide which distribution is the preferred one.

Proof (of lemma 3.6). The argument is based on our intuition above, and will look for which distribution is below the other in a right neighborhood of a. To simplify matters, however, let us "mirror" the functions around the vertical line at $x = a$ and look for which of $f(x), g(x)$ grows faster when x becomes larger than a, using an induction argument on the derivative order k. Clearly, whichever function grows slower for $x \geq a$ in the mirrored view is the $\preceq$-preferable one. Furthermore, we may assume $a = 0$ without loss of generality (as this is only a shift along the horizontal line). For $k = 0$, we have $f(0) < g(0)$ clearly implying that $f \preceq g$, since the continuity implies that the relation holds in an entire neighborhood $[0, \varepsilon)$ for some $\varepsilon > 0$. Thus, the induction start is accomplished.

For the induction step, assume that $f^{(i)}(0) = g^{(i)}(0)$ for all $i < k$, $f^{(k)}(0) < g^{(k)}(0)$, and that there is some $\varepsilon > 0$ so that $f^{(k)}(x) < g^{(k)}(x)$ is satisfied for all $0 \leq x < \varepsilon$. Take any such x and observe that

$$\begin{aligned} 0 > \int_0^x \left(f^{(k)}(t) - g^{(k)}(t)\right) dt &= f^{(k-1)}(x) - f^{(k-1)}(0) - \left[g^{(k-1)}(x) - g^{(k-1)}(0)\right] \\ &= f^{(k-1)}(x) - g^{(k-1)}(x), \end{aligned}$$

since $f^{(k-1)}(0) = g^{(k-1)}(0)$ by the induction hypothesis. Thus, $f^{(k-1)}(x) < g^{(k-1)}(x)$, and we can repeat the argument until $k = 0$ to conclude that $f(x) < g(x)$ for all $x \in [0, \varepsilon)$.

For returning to the original problem, we must only revert our so-far mirrored view by considering $f(-x), g(-x)$ in the above argument. The derivatives accordingly change into $\frac{d^k}{dx^k} f(-x) = (-1)^k f^{(k)}(x)$, and the proof is complete. $\square$

Lemma 3.6 has a useful corollary, which establishes the sought order-preserving property of the new sequences.

Corollary 3.7 *For $f \in C^\infty([0,a])$ being a probability distribution supported on $[0,a]$, define $\boldsymbol{y}(f) := ((-1)^k f^{(k)}(a))_{n\in\mathbb{N}}$. Then, for any two such distributions f, g, we have*

$$f \preceq g \iff \boldsymbol{y}(f) \leq_{lex} \boldsymbol{y}(g).$$

Proof. Let us consider the case when $\boldsymbol{y}(f) \neq \boldsymbol{y}(g)$ first. If so, then $f \preceq g$ must necessarily make $\boldsymbol{y}(f) \leq_{lex} \boldsymbol{y}(g)$; for otherwise, if $\boldsymbol{y}(f) >_{lex} \boldsymbol{y}(g)$, then lemma 3.6 would imply $g \prec f$, a contradiction.

Conversely, if $\boldsymbol{y}(f) <_{lex} \boldsymbol{y}(g)$, then lemma 3.6 implies $f \preceq g$ directly.

When $\boldsymbol{y}(f) = \boldsymbol{y}(g)$, then all derivatives are identical. This also means that f, g have identical Taylor-series expansions, and are therefore identical functions. Thus, $f \equiv g$ consequently. $\square$

The benefit of replacing an infinite sequence (a hyperreal number) by another infinite sequence of derivatives lies in possibility to approximate the density at its tail in a way that gets more and more accurate, depending on how many terms we include in the sequence. That is, we can easily resort to a Taylor-polynomial that approximates the tail-behavior of the density functions up to any precision we like. Moreover, the Gaussian density has the particular appeal of having all its distributions computable in closed form expressions by Hermite-polynomials. The n-th such polynomial can be constructed recursively as

$$H_{n+1}(x) := 2xH_n(x) - 2nH_{n-1}(x), \quad \text{with } H_0(x) = 1, H_1(x) = 2x.$$

These are related to the n-th order derivative of the Gaussian density via the identity

$$(-1)^n e^{\frac{x^2}{2}} \frac{d^n}{dx^n} e^{-\frac{x^2}{2}} = 2^{-\frac{n}{2}} H_n\left(\frac{x}{\sqrt{2}}\right).$$

In `R`, the Hermite-polynomials can be computed by the `orthopolynom` package [4].

3.3 Numerical Aspects

Now, to ultimately fix the convergence issue of FP over the hyperreals, we apply corollary 3.7 on the *truncated* series of moments, or equivalently, to a t-th order Taylor-polynomial fitted to the kernel densities under investigation.

However, two more aspects deserve attention:

- any floating point representation of the derivatives is inevitably subject of a rounding errors, say the machine precision $\varepsilon_M > 0$.

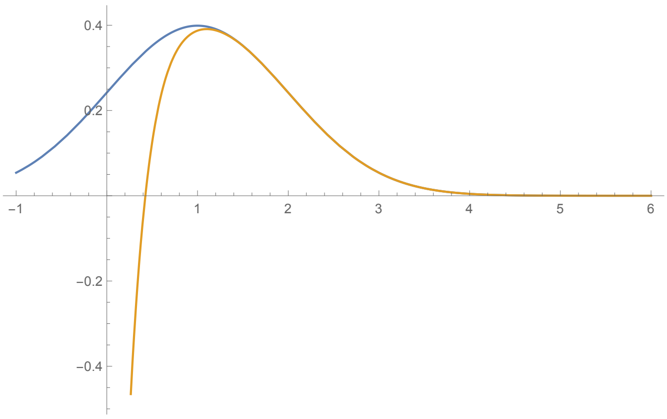

Figure 4: Approximating a $N(1,1)$-density at $x = 4$ by a Taylor polynomial of order 20

- the number of necessary terms in the Taylor-series expansion at $x = a$,

$$\hat{f}(x) = \hat{f}(a) + \sum_{k=1}^{\infty} \frac{\hat{f}^{(k)}(a)}{k!}(x-a)^k \tag{12}$$

  may grow too large to be practically feasible (see figure 4 for an approximation that includes derivatives up to order 20 but is quite inaccurate near the origin $x = 0$). However, given that we only need a good tail-approximation, the quality of the approximation far from the end of the support may be practically negligible. In other words, the result of the comparison remains somewhat insensitive against approximation errors far from the tail.

The roundoff error can directly be computed from (12), since the approximate version $\hat{f}_\varepsilon$ differs from $\hat{f}$ by at most

$$\max_{x\in[0,a]} \left|\hat{f}(x) - \hat{f}_\varepsilon(x)\right| \leq \varepsilon_M + \sum_{k=1}^{\infty} \frac{\varepsilon_M}{k!} a^k = \varepsilon_M \cdot e^a,$$

and can thus be kept under control by keeping the roundoff error small, according to the (known) value a (where all distributions are jointly truncated).

Given that all derivatives can (numerically) be represented with ℓ-bit floating point words in a suitable excess representation[2], we can cast the (now finite) sequence $\boldsymbol{y}(f) := ((-1)^k f^{(k)}(a))_{n\in\mathbb{N}}$ from corollary 3.7 into a real number by concatenating the bit representations of the numbers into a new value

$$y(f) := f(a) \| - f'(a) \| f''(a) \| - f'''(a) \| \ldots \| (-1)^t f^{(t)}(a), \tag{13}$$

[2]There will be no need to implement such a representation in reality, as we require it only for our theoretical arguments; however, an excess representation makes the numerical order identical to the lexicographic order, provided that the sign bits are chosen suitably (i.e., a 0 indicating a negative number, and 1 marking a positive number).

where t is the number of derivatives used, $\|$ denotes the bitstring concatenation, and all entries are rounded floating point values (in excess representation). The representation of a density function f by a real number of the form (13) has its numerical order (say between $y(f), y(g)$) being identical to the lexicographical order on the respective sequences/strings (as the highest order bits determine the order, and upon equality, the lower order bits come into play, etc.). Thus – in theory – we can safely use $y(f)$ as an approximate and order-preserving replacement for the density function f.

More importantly, the representation (13) is somewhat "retained" for mixed distributions like (9), as algorithm 1 constructs: assume that in (13), all entries $(-1)^i f^{(i)}(a)$ range within $0 \leq \left|f^{(i)}(a)\right| \leq M$ for some constant M. This constant must exist, as it is merely the maximum among all suprema that the derivatives up to order t attain on the (compact) interval $[0, a]$ (recall that our Gaussian kernel density estimator is continuously differentiable). Since the convex combination (9) of distributions by linearity of the differentiation amounts to a convex combination of the respective derivatives, the results must all stay within the same numeric range $[-M, +M]$, thus adding up and averaging the payoffs does not create a "carry" from the part $(-1)^i f^{(i)}(a)$ into the left neighboring part $(-1)^{i-1} f^{(i-1)}(a)$ within the bitpattern of expression (13). Thus, the representation remains valid also for the mixes (9) maintained over the execution of FP.

Matters of implementation can be further simplified: Since the numeric order of the number $y(f)$ given by (13) is the same as the lexicographic order on the truncated sequence $\widetilde{\boldsymbol{y}}(f) = ((-1)^i f^{(i)}(a))_{i=0}^t$, we can more conveniently work in practice with the vector representation $\boldsymbol{y}(f)$, although the formal convergence argument for the fictitious play algorithm is established in the standard setting over the reals, a we can use $y(f)$ in theory.

In other words, we use the real value $y(f)$ and $\leq$-relation on the payoffs in theory, while working with (the truncated) vector-version $\widetilde{\boldsymbol{y}}(f)$ and $\leq_{lex}$ relation in practice, when running FP. Convergence then follows from Robinson's results [11].

Having assured convergence of fictitious play by switching back to numeric approximate representations $y(f)$ of distribution functions f, it remains to establish the *correctness* of the approximate equilibrium returned by the so-modified algorithm. This easily follows from what we have obtained so far:

> *Informally*: A strategy profile is an "approximate" equilibrium in the game with hyperreal (distributional) payoffs F_{ij}, if and only if it is an equilibrium in the game over the reals, using $y(f_{ij}) \in \mathbb{R}$ as given by (13) in its payoff structure.

The following reasoning assumes a minimizing first player:

"$\Rightarrow$": Let $\boldsymbol{p}^* = (\boldsymbol{p}_1^*, \ldots, \boldsymbol{p}_n^*)$ be a Nash-equilibrium (in mixed strategies) in the n-person game with distribution-valued payoff distribution functions $F_i(\boldsymbol{p}_i, \boldsymbol{p}_{-i}^*)$. To ease notation, let us write $f_i(\boldsymbol{p}_i, \boldsymbol{p}_{-i}^*)$ to mean the respective density function. Since $\boldsymbol{p}_i^*$ is an equilibrium strategy for player i, we have $\forall i \forall \boldsymbol{p} : F_i(\boldsymbol{p}_i^*, \boldsymbol{p}_{-i}^*) \preceq F_i(\boldsymbol{p}, \boldsymbol{p}_{-i}^*)$. Since both sides are themselves distribution functions, corollary 3.7, tells that their approximate representatives satisfy $y(f_i(\boldsymbol{p}_i^*, \boldsymbol{p}_{-i}^*)) \leq y(f_i(\boldsymbol{p}, \boldsymbol{p}_{-i}^*))$.

"$\Leftarrow$": Conversely, if $y(f_i(\boldsymbol{p}_i^*, \boldsymbol{p}_{-i}^*)) \leq y(f_i(\boldsymbol{p}, \boldsymbol{p}_{-i}))$, then we may distinguish two cases:

1. An identity $y(f_i(\boldsymbol{p}_i^*, \boldsymbol{p}_{-i}^*)) = y(f_i(\boldsymbol{p}, \boldsymbol{p}_{-i}^*))$ implies that the respective Taylor-polynomial (approximations) are also identical, and thus the density $f_i(\boldsymbol{p}_i^*, \boldsymbol{p}_{-i}^*)$ is identical to the density $f_i(\boldsymbol{p}, \boldsymbol{p}_{-i}^*)$. Hence, $F_i(\boldsymbol{p}_i^*, \boldsymbol{p}_{-i}^*) \preceq F_i(\boldsymbol{p}, \boldsymbol{p}_{-i}^*)$ in particular.

2. Strict inequality $y(f_i(\boldsymbol{p}_i^*, \boldsymbol{p}_{-i}^*)) < y(f_i(\boldsymbol{p}, \boldsymbol{p}_{-i}^*))$, however, implies $F_i(\boldsymbol{p}_i^*, \boldsymbol{p}_{-i}^*) \preceq F_i(\boldsymbol{p}, \boldsymbol{p}_{-i}^*)$ by corollary 3.7, as before.

We may thus conclude that an approximate Nash-equilibrium can be computed by fictitious play through a double approximation:

1. We approximate the unknown (real) payoff distribution by a Gaussian kernel density estimator and truncate all the payoff estimators at the same value a.

2. We fit a Taylor-polynomial of order t at $x = a$ to enable the decision of the $\prec$-relation on the approximation by use the the vector of derivatives $(-1)^i \cdot f^{(i)}(a)$ for $i = 0, 1, \ldots, t$.

Remark 3.8 *We stress that the above arguments* are not *a theorem so far, as the quality of approximation of the resulting Nash equilibrium compared to the theoretically exact result remains undetermined. Indeed, both, the kernel density estimate and the Taylor polynomial get more and more accurate the more points we sample (for the kernel density) or the more derivatives we take into account (for the Taylor approximation). A precise error estimate, however, is involved and beyond the scope of this current article (subject of future investigations). Hence, we refrain from calling the last findings a "theorem" or similar here. Still, it must be emphasized that this double approximation can be made arbitrarily accurate, where $\preceq$ is decided.*

Discrete Distributions To handle discrete distributions, we can "abuse" the kernel density estimators in a straightforward fashion, as the effect of incorporating more and more samples at few fixed positions leads to a set of Dirac-masses accumulating at exactly the points of the support. Somewhat roughly speaking, this causes no immediate harm, since the likelihood $\Pr(X \leq k) = \sum_{i=0}^{k} \Pr(X = i)$ can quite well be approximated by integrating Gaussian densities from 0 to k, provided that the bell curve has its mass centred slightly left of the integer point (say, half-spaced between the integers), so that almost all the probability mass concentrates between two integers (this is somewhat similar to an empirical histogram, with the exception that it creates Dirac-masses instead of bars in the diagram).

The so-far sketched method thus remains applicable.

3.4 Remaining Possible Convergence Issues

Although fictitious play generally will converge, it may not do so in reasonable time, as experimental findings showed. Let us view the computation from a different angle: by Lemma 3.6 and also Theorem 3 in [14], we can replace a loss distribution F by some

(possibly infinite) vector $\boldsymbol{f} = (f_1, f_2, \ldots)$ so that $F \preceq G$ if and only if $\boldsymbol{f} \leq_{lex} \boldsymbol{g}$. Let us in the following consider these vectors as representatives of our loss distributions, and look at their coordinates. Fictitious play, in the initial stage, is merely a game being played on the first coordinate f_1 of the payoff structures until it converges. By then, it breaks the tie and moves on to the second coordinate f_2, to continue the game from there, further adapting the strategies for both players. This, however, may invalidate the optimum found this far, thus causing FP to return to the last coordinate (f_1) again to fix the issue. This means that, given any accuracy by which FP would stop iterating, we would end up with cycling between the first and the second coordinate, until both have been optimized. Not until these two converged, we would move further to the third coordinate in the lexicographic order, and so on. This can practically mean that FP may still take an infeasibly large number of iterations, unless the accuracy is set sufficiently coarse to accept ties within a certain proximity of the actual optimum.

The view of lexicographic optimization as a stack of games, however, also leads to an exact method to compute the optimum, using linear programming in the next section.

3.5 Exact Solution by Linear Programming

It is a standard exercise in game theory to convert the saddle point problem

$$\min_{\boldsymbol{x}} \max_{\boldsymbol{y}} \boldsymbol{x}^T \boldsymbol{A} \boldsymbol{y}$$

with $\boldsymbol{A} \in \mathbb{R}^{n \times m}$ into the linear program

$$\begin{aligned} \text{maximize} \quad & v \\ \text{subject to} \quad & v \leq \boldsymbol{x}^T \boldsymbol{A} \boldsymbol{e}_j && \text{for all } j = 1, 2, \ldots, m; \\ & x_1 + x_2 + \ldots x_n = 1; \\ & x_i \geq 0 && \text{for all } i = 1, 2, \ldots n. \end{aligned} \tag{14}$$

using $\boldsymbol{e}_i$ as the i-th unit vector in $\mathbb{R}^n$.

Now, taking into account that $\preceq$-optimization is actually a matter of optimization respecting the lexicographic order, we actually have a stack of games with payoff matrices $\boldsymbol{A}_1, \boldsymbol{A}_2, \boldsymbol{A}_3, \ldots$, in which $\boldsymbol{A}_i$ is the matrix game using only the i-th coordinate in the vector-representation of the payoff distributions. For the example of Lemma 3.7, we would have $\boldsymbol{A}_k$ with entries $(-1)^k \cdot f_{ij}^{(k)}(a)$ for the strategy pair $(i, j) \in PS_1 \times PS_2$; in the discrete case, the entries would directly be the probability masses for the respective category.

Like for FP, we can then exactly compute an MGSS by solving (14) on the game $\boldsymbol{A}_1$ containing only the last coordinates in the payoff vectors. This delivers the saddle-point value v_1, and is the same is if FP would have taken infinitely many iterations to convergence.

Now, to avoid the tie breaking issue described in Section 3.4, we move on to optimize the next coordinate, but *preserving* what we have achieved so far. That is, the next

linear program will use the matrix $\boldsymbol{A}_2$, but demand any feasible solution $\boldsymbol{x}$, and hence also the optimum, to still satisfy the further constraint

$$\boldsymbol{x}^T \cdot \boldsymbol{A}_1 \leq v_1, \tag{15}$$

where the $\leq$ is w.r.t. to all rows in the vector-matrix product. This prevents invalidating our previous optimum if we go on minimizing the mass on the butlast loss category, or second coordinate in the vector representation.

From that point onwards, the process continues likewise, i.e., if the payoff distributions F_{ij} are represented by vectors $\boldsymbol{f}_{ij} \in \mathbb{R}^k$, we repeat the following steps, starting from $i = 1$:

1. Construct the payoff structure $\boldsymbol{A}_i$ by putting the i-th coordinate of $\boldsymbol{f}_{ij}$ into the ij-position of the matrix $\boldsymbol{A}_i$.
2. Construct the linear program (14) using $\boldsymbol{A}_i$.

   If $i > 1$, add the constraints $\boldsymbol{x} \cdot \boldsymbol{A}_j \leq v_j$ for $j = 1, \ldots, i-1$ to (14).
3. Solve the resulting optimization problem to obtain v_i. Put $i \leftarrow i+1$ and return to step 1 until all coordinates have been processed.

The vector $\boldsymbol{x}^*$ coming out of the final optimization is the sought multi-goal security strategy. The same procedure, only with the respective dual linear programs with the respectively changed constraint (15) applies for each (hypothetical) opponent to lex-order optimize its own goal in the zero-sum game against the defending player 0.

3.6 Implementation Hints for Algorithm 1 – Overview

As we noticed above, the implementation of algorithm 1 *cannot* be done plainly and using the decision method sketched in section 2. Instead, the following adaptations require a careful implementation:

1. Use Gaussian kernel density estimators to approximate the actual payoff distribution functions.
2. Fix a common cutoff point a and truncate all distributions in the entire payoff matrix at the value a.
3. Fix an order t and compute Taylor-polynomial approximations for all payoff distributions at the point $x = a$. To represent an (approximate) distribution in the payoff matrix, use the vector of derivatives with alternating signs (as defined before).
4. Implement algorithm 1 as it stands, using corollary 3.7 to perform the $\preceq$-comparisons (on the approximate representatives of the true payoff structure). The pointwise additions of densities herein can be applied just as they are to the vectors of derivatives instead (thanks to the linearity of the differentiation operators).

3.7 The case of $d > 1$ security goals

Our presentation here is directly adapted from [8]: given a two-player multi-objective Γ and its auxiliary game $\overline{\Gamma}$ as specified in [6], we first need to cast $\overline{\Gamma}$ into zero-sum form to make FP converge. To this end, recall that player 1 in Γ, who is player 0 in $\overline{\Gamma}$, has d goals to optimize, each of which is represented as another opponent in the auxiliary game $\overline{\Gamma}$. We define the payoffs in a *compound game* ("one-against-all", where the "one" is the defender, against "all" (hypothetical) adversaries) from the payoffs in $\overline{\Gamma}$, while making the scalar payoffs vector-valued to achieve the zero-sum property:

- player 0:

$$\begin{aligned}
&\overline{\boldsymbol{u}}_0 : PS_1 \times \prod_{i=1}^{d} PS_2 \to \mathbb{R}^d, \\
&\boldsymbol{u}_0(s_0, \dots, s_d) := (u_1^{(1)}(s_0, s_1), u_1^{(2)}(s_0, s_2), \dots, u_1^{(d)}(s_0, s_d))
\end{aligned}$$

- i-th opponent for $i = 1, 2, \dots, d$:

$$\overline{\boldsymbol{u}}_i = (0, 0, \dots, 0, -u_1^{(i)}, 0, \dots, 0). \tag{16}$$

Obviously, the "vectorization" of the opponents payoffs leaves the set of equilibria unchanged.

3.7.1 Prioritization of Security Goals

To numerically compute (one of) the MGSS, [3] prescribes to scalarize the multiobjective game into a single-objective game. Indeed, this scalarization is primarily for technical reasons and can be made arbitrary (under suitable constraints). However, it this degree of freedom in the choice on the scalarization allows us to *prioritize* the security goals of player 0: To each of his d goals, we assign a *weight* $\alpha_{01}, \dots, \alpha_{0d} > 0$ subject to the condition that $\sum_{i=1}^{d} \alpha_{0i} = 1$.

With these weights, the payoffs after scalarization are:

- for player 0: $f_0 = \alpha_{01}\overline{\boldsymbol{u}}_1 + \alpha_{02}\overline{\boldsymbol{u}}_2 + \cdots + \alpha_{0d}\overline{\boldsymbol{u}}_d$,
- for the i-th opponent, where $i = 1, 2, \dots, d$

$$\begin{aligned}
f_i &= \alpha_{01} \cdot 0 + \alpha_{02} \cdot 0 + \cdots + \alpha_{0,i-1} \cdot 0 + \alpha_{0i} \cdot (-u_1^{(i)}) + \alpha_{0,i+1} \cdot 0 + \alpha_{0d} \cdot 0 \\
&= -\alpha_{0i} \cdot u_1^{(i)}
\end{aligned} \tag{17}$$

Concluding the transformation, we obtain a scalar compound game

$$\overline{\Gamma}_{sc} = (\{0, 1, \dots, d\}, \{PS_1, \underbrace{PS_2, \dots, PS_2}_{d \text{ times}}\}, \{f_0, \dots, f_d\}) \tag{18}$$

from the original two-person multiobjective Γ with payoffs $u_1^{(1)}, \dots, u_1^{(d)}$ that can directly be plugged into expressions (16) and (17).

Towards a numerical computation of equilibria in $\overline{\Gamma}_{sc}$, we need yet another transformation due to [12]: for the moment, let us consider a general compound game Γ_c as a collection of d two-person games $\Gamma_c^{(1)}, \dots, \Gamma_c^{(d)}$, each of which is played independently between player 0 and one of its d opponents. With Γ_c, we associate a two-person game Γ_{cr} that we call the *reduced game*. The strategy sets and payoffs of player 0 in Γ_{cr} are the same as in Γ_c. Player 2's payoff in the reduced game is given as the *sum* of payoffs of all opponents of player 0 in the compound game Γ_c.

Lemma 3.9 ([12]) *A fictitious play process approaches equilibrium in a compound game Γ_c, if and only if it approaches equilibrium in its reduced game Γ_{cr}.*

So, it suffices to consider the reduced game $\overline{\Gamma}_{scr}$ belonging to $\overline{\Gamma}_{sc}$. It is a trivial matter to verify the following fact (by substitution).

Lemma 3.10 *The reduced game $\overline{\Gamma}_{scr}$ of the scalarized compound game $\overline{\Gamma}_{sc}$ defined by* (18) *is zero-sum.*

So by the convergence of FP in any zero-sum game (theorem 3.3), we obtain the final correctness result on FP when being applied to our $(d+1)$-person game $\overline{\Gamma}_{sc}$ that will deliver the sought MGSS:

Theorem 3.11 *The scalarized compound game $\overline{\Gamma}_{sc}$ defined by* (18) *has the fictitious play property.*

3.7.2 The Full Procedure

Theorem 3.11 induces the following procedure to compute MGSS according to definition 3.1:

Given a two-player multi-goal Γ with d payoffs $u_1^{(1)}, \dots, u_1^{(d)}$ for player 1 (and possibly unknown payoffs for player 2), we obtain an equilibrium in an MGSS along the following steps:

1. Assign strictly positive weights $\alpha_{01}, \dots, \alpha_{0d}$, satisfying $\sum_{i=1}^{d} \alpha_{0i} = 1$, to each goal, and set up the scalarized auxiliary compound game $\overline{\Gamma}_{sc}$ by virtue of expressions (16), (17) and (18).

2. Run fictitious play (algorithm 1) in $\overline{\Gamma}_{sc}$, stopping when the desirable precision of the equilibrium approximation is reached. Preferably for a non-approximate solution, solve a sequence of linear programs as described in Section 3.5.

3. The result vector $\boldsymbol{x}$ is directly the sought multi-criteria security strategy, whose assurances are given by the respective expected payoffs of the opponents (equation (10) in connection with the output $\boldsymbol{y}$ of the algorithm).

A few remarks about this method appear in order:

- The reduced scalarized game Γ_{scr} is a purely theoretical vehicle to establish convergence of FP in the scalarized (formerly multiobjective) multiplayer game $\overline{\Gamma}_{scr}$. Thus, FP is to be executed on $\overline{\Gamma}_{scr}$ by copying and adapting lines 15 until 19 (incl.) in algorithm 1 for each hypothetical adversary.

- Convergence of the game is guaranteed only on the Taylor-polynomial approximations for the densities $\boldsymbol{y}(f_{ij})$, but not on the true densities f_{ij} as such (for these, the arguments about convergence failure discussed around equation (11) and illustrated in example 3.5 remain intact).

- The obtained bounds v_{up} and v_{low} also refer to the Taylor-polynomial approximations for the respective bounds on the actual value v of the game over the distribution-valued payoffs. Thus, the actual assurance limits (distributions V_i given by equation (5) in definition 3.1) must be computed from the resulting mixed strategies (as specified in algorithm 1, line 23, i.e., the formula should be implemented for all d goals).

- FP and likewise linear programming on the multiplayer game does not deliver a concrete (Pareto-Nash) equilibrium strategy for the (single) physical adversary, but returns worst-case behavior strategy options for every of his d goals. Thus, the result may pessimistically underestimate what happens in reality (as the true adversary is forced to choose a single out of the multiple options, thus necessarily deviating from some of the d equilibrium strategies).

- The assurances obtained also need to be interpreted bearing in mind that here neither player follows the other. That is, the Pareto-Nash equilibrium would gives an optimal strategy for the attacker under the hypothesis that it plays a zero-sum game against the defender as if player 1 would have only this particular goal and no other. The real defender, however, will care about multiple goals at the same time, thus – by symmetry – deviating from the zero-sum equilibrium strategy that the attacker has in its own single-goal game. This means that the assurance obtained in the last step of the above procedure are *not* best replies to the optimal defense, but rather the worst-case that would be possible if the defender were to spend all its resources on this particular goal.

  Asking for a best reply to the defender's optimal multi-criteria strategy is a much simpler issue: for the attacker it merely means to adapt by picking the $\preceq$-maximum from the vector $(\boldsymbol{x}^*)^T\boldsymbol{A}$, when $\boldsymbol{A}$ is the weighted sum of all payoff structures for all goals (doing the scalarization), i.e., the payoff structure in the scalarized reduced game constructed in step 2 above. This is the case of a *leading-defender and following-attacker*, always giving a pure worst-case attack scenario, whose payoff is then (trivially) a bound to the defender's possible damage.

4 Summary and Outlook

Implementing the theoretical concepts introduced in [6] requires care to avoid a variety of subtle difficulties. While using Epanechnikov-kernel based density estimates greatly eases matters of plain payoff distribution $\preceq$-comparisons, such models are not useful at all for computing multi-goal security strategies. For these, it is practically advisable to use Gaussian kernels, being truncated at a common point. At first, this avoids paradoxical results (as outlined in section 2.5), but furthermore enables the application of conventional fictitious play. With example 3.5 demonstrating the failure of FP on $^*\mathbb{R}$ when implemented plainly, the algorithm can nevertheless be successfully applied to proper approximations of the game matrices. In particular, a double-approximation is applied here, where the first stage approximates the unknown true distribution by a kernel density estimate, and the second stage is a Taylor-polynomial expansion at the tails of the distribution. Despite this approximation performing possibly badly in areas of low damage (cf. figure 4), it provides a good account for the tail-dependent comparison that $\preceq$ is based on. In other words, even though the Taylor-polynomial is not accurate everywhere on the relevant interval of possible losses, it nevertheless produces correct $\preceq$-preferences as these depend on the tails of the distribution, where the Taylor-approximation is indeed quite accurate. The practical benefit lies in the ability of running conventional fictitious play on the so-approximated distribution models, after casting the game-matrix of distributions into a regular game matrix over the reals.

With the algorithmic aspects being covered in this report, companion (follow up) work will discuss the modeling and treatment of *advanced persistent threats*, and how to apply our algorithms to distributions with fat, heavy or long tails (all of which can be fed back into our algorithms after truncation, to mention one quick-and-easy solution).

Acknowledgment

This work was supported by the European Commission's Project No. 608090, HyRiM (Hybrid Risk Management for Utility Networks) under the 7th Framework Programme (FP7-SEC-2013-1). The author thanks Sandra König from AIT very much for valuable discussions and for spotting some errors, and also for providing the demonstration implementation of algorithm 1.

The author is also deeply indebted to Ali Alshawish and Vincent Brgin for having provided deep insights about the convergence of fictitious play along intensive experiments. The convergence issue described in Section 3.4 has been reported by them, and here repeated in the authors own words. The new computational method using linear programming was invented in response to this issue.

References

[1] J. L. Bell and A. B. Slomson. *Models and Ultraproducts: an Introduction.* North-Holland, 1971.

[2] Drew Fudenberg and David K. Levine. *The Theory of Learning in Games.* MIT Press, London, 1998.

[3] D. Lozovanu, D. Solomon, and A. Zelikovsky. Multiobjective games and determining pareto-nash equilibria. *Buletinul Academiei de Stiinte a Republicii Moldova Matematica*, 3(49):115–122, 2005. ISSN 1024-7696.

[4] Frederick Novomestky. orthopolynom: Collection of functions for orthogonal and orthonormal polynomials. https://cran.r-project.org/web/packages/orthopolynom/index.html, 2013.

[5] R Development Core Team. *R: A Language and Environment for Statistical Computing.* R Foundation for Statistical Computing, Vienna, Austria, 2011. ISBN 3-900051-07-0.

[6] S. Rass. On Game-Theoretic Risk Management (Part One) – Towards a Theory of Games with Payoffs that are Probability-Distributions. *ArXiv e-prints*, June 2015. http://arxiv.org/abs/1506.07368.

[7] S. Rass, S. König, and S. Schauer. Uncertainty in games: Using probability distributions as payoffs. In MHR Khouzani, Emmanouil Panaousis, and George Theodorakopoulos, editors, *Decision and Game Theory for Security, 6th International Conference, GameSec 2015*, LNCS 9406. Springer, 2015.

[8] S. Rass and B. Rainer. Numerical computation of multi-goal security strategies. In Radha Poovendran and Walid Saad, editors, *Decision and Game Theory for Security*, LNCS 8840, pages 118–133. Springer, 2014.

[9] Stefan Rass. On game-theoretic network security provisioning. *Springer Journal of Network and Systems Management*, 21(1):47–64, 2013.

[10] Abraham Robinson. *Nonstandard Analysis.* Studies in Logic and the Foundations of Mathematics. North-Holland, Amsterdam, 1966.

[11] J. Robinson. An iterative method for solving a game. *Annals of Mathematics*, 54:296–301, 1951.

[12] Aner Sela. Fictitious play in 'one-against-all' multi-player games. *Economic Theory*, 14:635–651, 1999.

[13] Alan Washburn. A new kind of fictitious play. Technical report, Operations Research Department, Naval Postgraduate School, Monterey, California 93943, 2001. Copyright by John Wiley & Sons, Inc.

[14] Stefan Rass, Sandra Konig, and Stefan Schauer. Decisions with Uncertain Consequences-A Total Ordering on Loss-Distributions. *PLoS ONE*, 11(12):e0168583, 2016.
